# Cancer and Aging Biomarkers: Classification, Early Detection Technologies and Emerging Research Trends

**DOI:** 10.3390/bios15110737

**Published:** 2025-11-04

**Authors:** Mi-Ran Ki, Dong Hyun Kim, Mohamed A. A. Abdelhamid, Seung Pil Pack

**Affiliations:** 1 Department of Biotechnology and Bioinformatics, Korea University, Sejong-Ro 2511, Sejong 30019, Republic of Korea; allheart@korea.ac.kr (M.-R.K.); jklehdgus@korea.ac.kr (D.H.K.); mohamed42@korea.ac.kr (M.A.A.A.); 2 Institute of Industrial Technology, Korea University, Sejong-Ro 2511, Sejong 30019, Republic of Korea; 3 Biology Department, Faculty of Education and Arts, Sohar University, Sohar 311, Oman

**Keywords:** biomarkers, biosensors, aging, cancer, Alzheimer’s disease, early detection, biological aging, liquid biopsy, artificial intelligence algorithms, Point-of-Care

## Abstract

Cancer and aging are two distinct biological processes with shared cellular pathways, such as cellular senescence, DNA damage repair, and metabolic reprogramming. However, the outcomes of these processes differ in terms of proliferation. Understanding biomarkers related to aging and cancer opens a pathway for therapeutic interventions and more effective prevention, detection, and treatment strategies. Biomarkers, ranging from molecular to phenotypic indicators, play an important role in early detection, risk assessment, and prognosis in this endeavor. This review comprehensively examines key biomarkers associated with cancer and aging, highlighting their importance in early diagnostic strategies. The review discusses recent advances in biomarker-based diagnostic technologies, such as liquid biopsy, multi-omics integration, and artificial intelligence, and emphasizes their novel potential for early detection, accurate risk assessment, and personalized therapeutic interventions in cancer and aging science. We also explore the current state of biosensor development and clinical application cases. Finally, we discuss the limitations of current early diagnostic methods and propose future research directions to enhance biomarker-based diagnostic technologies.

## 1. Introduction

The global population is undergoing an unprecedented structural shift, with people aged 65 and over representing the fastest-growing segment [1,2,3]. This shift poses an urgent public health challenge, as aging is the most significant risk factor for chronic diseases, particularly cancer [1,4,5].

The cancer incidence in people over 65 is 11 times higher than in younger people. More than 60% of newly diagnosed cancer patients belong to this age group, highlining the inextricably linked between aging and cancer [6,7]. Both processes share common hallmarks, including genomic instability [8,9], cellular senescence [10,11], telomere dysfunction [12,13], chronic inflammation [14,15], autophagy [16,17], and dysregulated metabolic pathways [18,19]. Yet they diverge in their ultimate cellular fate and tissue-level outcomes, offering crucial insights into how aging and cancer overlap and differ [20].

Traditionally, cancer research has focused on chronological age. However, biological age is now recognized as a more meaningful measure of health and disease risk [21]. Biological age reflects the accumulation of molecular and physiological changes over time, providing a better indicator of an individual’s true vulnerability [22,23].

The heterogeneity of aging processes within age cohorts is a critical consideration. The concept of “ageotypes” highlights distinct aging patterns influenced by genetic predispositions and environmental factors [24]. This leads to varied health outcomes, even among people of similar ages. This inherent variability underscores the need for biomarkers that distinguish individuals of the same chronological age but with different biological aging profiles, enabling more precise and personalized interventions [24].

Biomarker discovery has become central to modern precision medicine [25]. Imaging technologies such as magnetic resonance imaging (MRI), positron emission tomography (PET), and computed tomography (CT) are increasingly being integrated with molecular biomarkers to refine diagnosis and treatment decisions [25]. Recent advances in high-throughput technologies, including genomics, proteomics, metabolomics, and multi-omics integration, as well as artificial intelligence (AI) have accelerated the discovery of novel biomarkers that can distinguish normal aging from disease-related changes [26].

Early cancer detection remains one of the most effective strategies for improved treatment outcomes, extended survival rates, and reduced patient suffering and economic burden [27]. Approximately 50% of cancers are still diagnosed at advanced stages, when treatment success is limited [27]. For example, when breast cancer (BC) is diagnosed at its earliest stage, the 5-year survival rate is approximately 100%. In contrast, late-stage diagnosis reduces survival to about 30% [28]. A similar trend is seen in colorectal cancer (CRC), where early detection ensures survival rates above 90%, but late detection drops survival to 10% [28]. These significant contrasts underscore the urgent global need for safe, cost-effective, accessible early diagnostic methods [29,30,31].

Throughout the entire cancer treatment process, biomarkers play indispensable roles. They support risk assessment, early detection, treatment selection, therapy monitoring, and recurrence prediction [25]. By aligning treatment with each patient’s molecular profile, biomarkers form the foundation of personalized medicine [32].

Importantly, cancer disproportionately affects older adults. The majority of cases occur in those over 50 [33]. However, this population often faces additional challenges, including multiple comorbidities, immune changes, and increased inflammation, all of which can obscure or complicate diagnosis [34]. Such factors can also affect the interpretation of biomarkers, making age-specific validation studies essential to maintain diagnostic sensitivity (SN) and specificity (SP).

**Figure 1:**
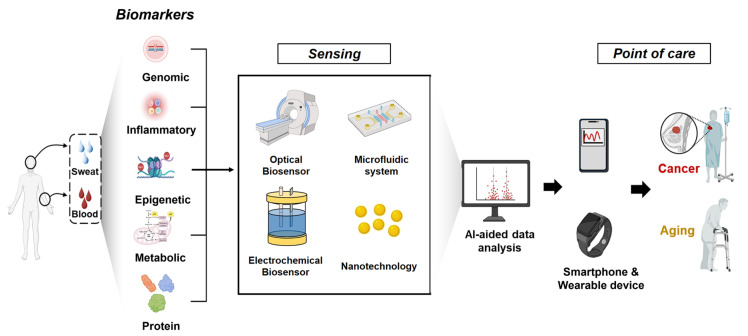
Schematic illustration of the integrated non-invasive biomarker detection and AI-assisted biosensing workflow. The process begins with non-invasive sampling, such as liquid biopsy (blood, saliva, or urine collection), followed by biomarker sensing and signal transduction through advanced bio-sensing platforms. The detected signals are processed using AI-driven data analysis and transmitted to smartphones or wearable devices, enabling individuals to monitor disease biomarkers in real time and receive personalized medical guidance or early intervention.

This review provides a comprehensive overview of the shared and distinct biological mechanisms underlying aging and cancer, explores key biomarkers, details recent advancements in diagnostic technologies leveraging these biomarkers, and discusses current limitations and future research directions (Figure 1).

## 2. Shared Biological Mechanisms Between Aging and Cancer

**Figure 2:**
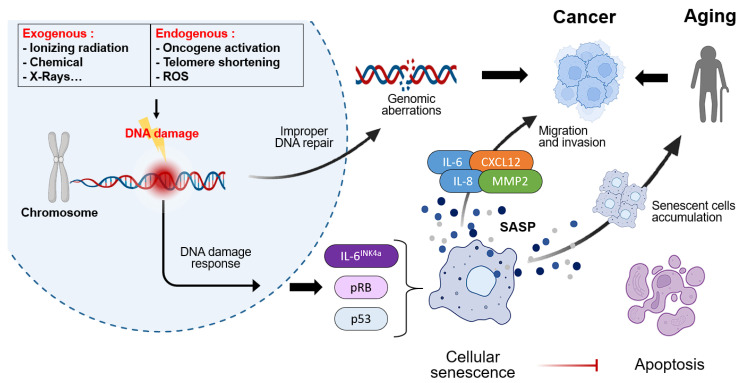
Cancer and aging shared biological mechanism. Exogenous factors such as ionizing radiation, chemicals, and X-rays, as well as endogenous factors including oncogene activation, telomere shortening, and ROS, can induce DNA damage. The DNA damage response is activated to maintain genomic integrity; however, improper or incomplete DNA repair results in genomic aberrations that promote cancer. Persistent DNA damage response signaling also induces the expression of IL-6INK4a, pRB, and p53, leading to cellular senescence. Senescent cells (SnCs) secrete SASP fac-tors—including IL-6, IL-8, CXCL12, and MMP2—which contribute to chronic inflammation, enhanced cell migration and invasion, and tissue microenvironment remodeling. These processes collectively accelerate both cancer development and the aging process through the accumulation of SnCs and reduced apoptosis.

Cancer and aging are closely related to health and disease, being two of the most significant biological processes in the human body. While aging and cancer may appear to be independent processes, recent research suggests that there is a clear interaction between the two (Figure 2). From a mechanistic perspective, the processes of aging and cancer exhibit numerous similarities, including genomic instability, accumulation of DNA damage, cellular senescence, telomere dysfunction, and chronic inflammation. These factors act as links between the two processes [20,35,36].

### 2.1. Genomic Instability

In aging, genomic instability is known to cause functional decline in tissues and organs through a variety of mechanisms, including DNA damage, replication errors, and epigenetic changes [9]. The term “DNA damage” encompasses a variety of alterations, including base substitutions, deletions, chromosomal abnormalities, and mitochondrial DNA mutations [37,38]. These alterations can compromise genome integrity and contribute to the development of age-related diseases. Specifically, DNA damage induced by oxidative stress has been demonstrated to expedite telomere shortening and mitochondrial dysfunction, thereby exacerbating cellular aging [39,40].

In the context of cancer, genomic instability exhibits a paradoxical role. The process under scrutiny has been shown to promote tumor initiation through mutations in oncogenes and tumor suppressor genes [41]. Furthermore, it has been demonstrated that this process provides the genetic diversity necessary for tumor evolution and treatment resistance. In contrast to the process of aging, where cellular senescence functions as a protective mechanism to prevent the propagation of damaged DNA, cancer cells evade cellular senescence and apoptosis through dysregulation of checkpoint pathways such as p53 and retinoblastoma (RB) protein [42]. This evasion enables genetically unstable cells to proliferate uninhibitedly, ultimately resulting in tumor formation [43].

A notable finding is the observation that the mechanisms of genomic instability observed in aging and cancer often overlap [44]. Key elements in both processes include impaired DNA repair capacity, telomere dysfunction, replication stress, and mitochondrial-derived reactive oxygen species (ROS) production [45]. However, while genomic instability in aging primarily leads to functional decline and organismal frailty, in cancer it promotes clonal expansion and malignant formation [46].

#### 2.1.1. DNA Damage Accumulation

Genomic DNA and mitochondrial DNA are both susceptible to damage from continuous endogenous factors, such as reactive oxygen species and aldehydes, as well as exogenous factors, including chemicals in the environment, ultraviolet (UV) rays, and X-rays. Prolonged exposure to this type of damage can result in genomic instability, which, in turn, can trigger a cascade of events leading to cell apoptosis or senescence [47]. Normal cells possess a highly efficient DNA damage repair system that prevents damaged DNA from being replicated; however, this ability declines during the aging process. In the context of cancer, the impairment of repair mechanisms and the subsequent damage to the genome are exploited, resulting in mutations in critical genes that trigger uncontrolled cell proliferation [48].

Oxidative stress, a primary endogenous factor in the development of DNA damage, is a state primarily caused by the accumulation of ROS originating from mitochondria. ROS oxidizes the guanine base of DNA, forming 8-hydroxydeoxyguanosine (8-OHdG). The occurrence of DNA sequence mutations is attributed to the pairing of 8-OHdG with adenine rather than cytosine during the process of DNA replication [49,50]. Furthermore, ribosomal DNA (rDNA) loci are susceptible to damage by internal and external factors, including ROS and UV [51]. To prevent a decrease in the number of copies or the accumulation of extrachromosomal ribosomal DNA circles (ERCs), which can be toxic, during the normal aging process, rDNA loci are strictly regulated [52]. However, it is well-documented that rDNA locus damage can induce cellular aging by acting as a genomic trigger [53].

Furthermore, genomic instability at the base level, including base substitutions and small base insertions/deletions, has been observed in various cancers. At the chromosomal level, large-scale chromosomal losses and gains occur [45]. These alterations ultimately result in malignant evolution of tumor cells. With advancing age, the efficacy of the DNA repair system is compromised, resulting in the accumulation of DNA damage within cells and an elevated risk of disease onset [54]. For instance, breast epithelial cells in women with genetic defects in breast cancer type 1 (BRCA1) or BRCA2 genes exhibit reduced DNA repair capacity, resulting in an increased risk of breast and ovarian cancer, as well as accelerated aging [55,56]. Furthermore, alterations in the DNA repair process have been implicated in the aging process in mice and the etiology of various progeroid diseases in humans. It has been demonstrated that particular genetic syndromes, including Cockayne syndrome [57,58], xeroderma pigmentosum [59,60], Bloom syndrome [61], ataxia telangiectasia [62], Werner syndrome [63], and Fanconi anemia [64], have been associated with an elevated risk of developing cancer.

Mitochondrial DNA (mtDNA) is also susceptible to mutations and deletions caused by internal and external factors, which are linked to cancer and aging [65]. Studies of mouse and human cells have shown that mitochondrial dysfunction caused by ROS damage is a major cause of age-related mitochondrial dysfunction. For instance, mice deficient in DNA polymerase γ exhibit mtDNA deletions that lead to rapid aging and a shortened lifespan [66,67]. Additionally, human mtDNA damage diseases exhibit phenotypes that partially mimic the aging process. Mitochondrial damage is an important step in cancer development [68]. It leads to genetic mutations and the production of oncometabolites. Mitochondrial damage also promotes cancer development through metabolic reprogramming and changes in mitochondrial dynamics [68].

#### 2.1.2. Chromosomal Aberrations

Chromosomal aberrations can be classified as either structural or numerical abnormalities. Numerical abnormalities refer to changes in the normal number of chromosomes, a condition called aneuploidy. The most common chromosomal number changes are monosomy, in which only one chromosome is present in a pair, and trisomy, in which three chromosomes are present instead of one pair. Structural abnormalities can alter chromosome structure through four types of abnormalities: deletion, duplication, inversion, and translocation [69,70,71,72]. These abnormalities can lead to dysfunction and disorders of the affected chromosome.

In aging, numerical abnormalities cause a decline in cell and tissue function, leading to cellular aging. Highly aneuploid cells enter a state of cellular aging characterized by permanent cell cycle arrest at the G1/S or G2/M phase and enhanced secretory properties [73,74]. Studies on yeast and human cells indicate that imbalanced chromosome numbers alter protein homeostasis and impair the function of large protein complexes involved in replication, mitosis, and metabolism. This metabolic stress generates ROS. Ultimately, highly aneuploid cells, involving the acquisition or loss of multiple chromosomes, enter a state of cellular senescence, which is characterized by permanent cell cycle arrest and enhanced secretory properties [75,76,77]. In many pathological situations, mutations that tolerate aneuploidy, such as p53 mutations in mammalian cells or heterozygous deletions of pro-apoptotic genes in Drosophila, allow aneuploid cells to persist within tissues [78]. In such cases, cells undergo c-Jun N-terminal kinase (JNK)-dependent, autonomous, epithelial-to-mesenchymal transition (EMT)-like conversion and proceed to a similar senescent state [79,80].

Chromosomal aberrations, which also occur in cancer, are primarily observed in solid tumors of epithelial origin. Chromosomal aberrations initiate the process of acquiring chromosomes that carry oncogenes and the subsequent loss of chromosomes that contain tumor suppressor genes [81]. This is one of the key mechanisms that promotes tumor cell heterogeneity and malignancy, going beyond mere genetic mutations [82]. Numerical chromosomal abnormalities are common in tumors and disrupt gene expression, inducing protein dysfunction and leading to the loss of tumor suppressor genes and the overexpression of oncogenes. Structural chromosomal abnormalities can also cause various diseases [83]. For instance, chromosomal translocation can result in the formation of a new hybrid gene called the Philadelphia chromosome [84]. This chromosome is formed by the translocation of the Bcr-Abl hybrid gene, and the resulting abnormal protein increases the rate of cell division, which can lead to chronic myeloid leukemia [85]. Wilms’ tumor and RB are other examples of cancers caused by chromosomal deletions [86,87]. Therefore, many types of cancer are closely related to deletions and mutations of chromosomes where tumor suppressor genes are located.

### 2.2. Cellular Senescence

Cellular senescence can be broadly categorized into two distinct types: replicative senescence and stress-induced premature senescence [88]. Replicative senescence is a form of cellular aging induced by telomere shortening, observed in various cell types (e.g., infant cells, glial cells, keratinocytes, endothelial cells, lymphocytes, etc.), leading to defects in cell proliferation and cell cycle arrest [89]. Concurrently, stress-induced premature senescence is triggered by stress-inducing factors such as oxidative stressors, mitochondrial dysfunction, DNA damage, and the senescence-associated secretory phenotype (SASP) produced by immature cells that activates the p16 or p53 pathway [90,91]. Cellular senescence is not characterized by a singular or uniform biomarker. Rather, it is delineated by a multitude of discrete, non-overlapping indicators [92].

#### 2.2.1. Senescence-Associated Secretory Phenotype (SASP)

The role and mechanisms of SASP, a representative biomarker of cellular senescence, are gaining attention for their potential association with various diseases [93]. SASP is distinguished by a distinctive secretory profile, comprising a variety of secreted proteins, cytokines, chemokines, growth factors, and proteases [94]. SASP factors exert their effects through two distinct secretion pathways. The initial secretion mode is autocrine, wherein SASP factors promote the senescence of the senescent cell itself. The second mode of action is paracrine, whereby it acts as a side-secreted senescent factor that induces senescence in neighboring cells rather than directly causing cellular senescence [95]. A number of studies have been carried out in an attempt to identify the components of SASP; however, its precise composition remains incompletely understood [96]. The composition and levels of SASP can vary significantly depending on the aging-inducing stimulus, the type of aging cells involved, and the duration of aging [97]. A substantial body of research has demonstrated that SASP plays a pivotal role not only in the aging process but also in the development of cancer. Various cytokines within SASP have been reported to exert distinct effects on tumor cells and the tumor microenvironment (TME) [98]. The following section will introduce the several components of the SASP.

Interleukin-1 (IL-1) is a pro-inflammatory cytokine that plays a pivotal role in regulating the expression of senescence-associated secretory phenotype (SASP) factors, although it is not essential for the induction of cellular senescence itself. Both IL-1α and IL-1β activate SASP through the same IL-1 receptor (IL-1R) signaling pathway. Notably, IL-1β has been identified as a key regulator capable of independently driving SASP expression [99]. Recent studies have shown that the expression of the disruptor of telomeric silencing 1-like (DOT1L) gene is required for SASP gene activation. This may occur through enhanced methylation of histone H3 at lysine 79 (H3K79me2/3) specifically at the IL-1α gene locus [100]. Furthermore, studies have demonstrated that non-adaptive proximal tubule epithelial cells exposed to hypoxic conditions and IL-1β treatment exhibit hallmark features of cellular senescence [101]. These include increased expression of p21, elevated activity of senescence-associated β-galactosidase (SA-β-gal), and enhanced secretion of inflammatory and fibrotic SASP factors.

IL-6, another type of pro-inflammatory cytokine, is known to be a component of the SASP. IL-6 has been demonstrated to amplify cellular senescence in SASP-producing cells through autocrine action. The IL-6 protein is amplified through intracellular mechanisms, interacting with cytoplasmic DNA, the cyclic GMP-AMP synthase/stimulator of interferon genes (cGAS/STING) pathway, and nuclear factor kappa-light-chain-enhancer of activated B cells (NF-κB) activation to induce aging within the cell [96]. The cellular aging mechanism of IL-6 was discovered to induce aging in hepatocellular carcinoma cells by inhibiting the IL-6/signal transducer and activator of transcription (STAT) pathway via atorvastatin [102]. In addition, it was demonstrated that IL-6 deficiency in mice results in the exacerbation of liver cancer, which is associated with a substantial impairment of the SASP and aging [103].

Galectin-9, a protein that binds to various cell receptors, performs multiple functions, including immune regulation, inflammatory responses, and cell survival and death [104]. Tarallo et al. initially reported that Galectin-9 is a component of SASP secreted by SnCs in melanoma [105]. A body of research has emerged on Galectin-9, indicating its capacity to exert immunosuppressive effects within the TME. This research suggests that Galectin-9 promotes apoptosis in T cells and monocytes, enhances the regulation of T cells, helper T cells, and M2 macrophages, and suppresses antitumor immune responses [106,107].

Extracellular vesicles (EVs) are a type of membrane-bound structure that are released by all cells and are known for their role in removing cellular waste [108]. However, subsequent studies on EVs revealed their ability to transmit DNA, RNA, proteins, mitochondria, lipids, and other components between cells, performing diverse functions including cellular signaling [109]. In recent years, EVs have gained recognition as pivotal mediators within the SASP [98]. Research has documented their multifaceted functions in the aging process. Inhibition of small EV secretion has been demonstrated to accumulate DNA damage in SnCs and induce cell death-like phenomena [110]. Consequently, the vesicular secretome exerts dual-edged effects, contingent upon the cellular milieu. This effect is mediated by various EV compounds, including proteins, nucleic acids, and lipids, thereby supporting the functional role EVs perform in aging [111].

#### 2.2.2. Tumor-Suppressive vs. Tumor-Promoting Roles

Various SASP factors have multiple biological activities, and the SASP has paradoxical functions within the TME. The SASP can promote or suppress tumor growth; these contradictory functions arise due to pathway regulation driven by the dominant expression of SASP factors [112].

SASP factors primarily exhibit tumor-promoting effects in the TME. In particular, they play an initial role in promoting tumor cell proliferation. Aged human fibroblasts can accelerate tumorigenesis in neoplastic epithelial cells by stimulating the proliferation and progression of preneoplastic epithelial cells via SASP factors. Early studies demonstrating the tumorigenic role of SASP in cellular senescence originated with human dermal fibroblasts (HDFs) [113]. A study by Di et al. suggested that aged mesenchymal stem cells (MSCs) accelerate the hyperproliferation and migration of breast cancer cells via IL-6/STAT-3-dependent signaling more than young cells do [114]. Aged breast luminal cells secrete SASP, including IL-6 and IL -8, which activate stromal fibroblasts via the STAT3 pathway. This induced tumor formation through a paracrine mechanism [115]. Other study has identified EVs, a type of SASP factors, as a key mediator of tumor promotion. Aging cells promote cancer cell proliferation by increasing Ephrin receptor A2 (EphA2) in small EVs, thereby activating the mitogen-activated protein kinase (MAPK) pathway [116]. Thus, SASP induces cellular senescence and directly promote4s tumorigenesis and the proliferation of precancerous cells.

It has been established that SASP contributes to tumor progression by inducing EMT, among its various functions in the TME. For instance, in the context of breast cancer, aged fibroblasts secrete factors from the SASP, including IL-6 and IL-8. These factors have been observed to increase vimentin expression, decrease E-cadherin, and induce a reduction in cytokeratin (CK) in non-invasive breast cancer cells [117]. Normal human epidermal keratinocytes undergo the process of aging during in vitro culture, exhibiting low rates of spontaneous escape and immortalization. However, when exposed to SASP factors secreted by aged HDFs, these cells exhibit enhanced escape frequency and display EMT and matrix metalloproteinase (MMP)-mediated motility [118]. This phenomenon has also been observed in pancreatic epithelial cells. When these cells were exposed to aged condition medium, spindle-shaped cells were observed, exhibiting enhanced invasion potential [119]. SASP factors such as MMP-3 secreted by senescent fibroblasts inhibited the normal differentiation of non-malignant breast epithelial cells while promoting the complete malignant transformation of precancerous epithelial cells, suggesting that SASP contributes to the loss of tissue function and organization, leading to aging-related diseases and cancer [120].

SASP is a key component of the TME, attracting immunosuppressive immune cells that facilitate the immune clearance of tumor cells while simultaneously inducing immune cell-mediated inflammatory responses that can promote tumor progression. For instance, study by Eggert et al. demonstrated that chemokines secreted by aged hepatocytes exhibit an inhibitory effect during the early stages of hepatocellular carcinoma (HCC), but conversely accelerate the growth rate of fully developed HCC cells [121]. Furthermore, Lau et al. indicated that IL-1 within SASP attracts pancreatic macrophages, suggesting that tissue-resident macrophages may promote pancreatic cancer progression [122].

SASP can function to promote tumors, but conversely, it also performs tumor-suppressing roles [123]. SASP has been demonstrated to exert its anti-tumor function by maintaining tumor cell cycle arrest [124]. While cell cycle arrest in cellular senescence is generally considered irreversible, tumor SnCs can re-enter the cell cycle [125]. In cellular senescence, cell cycle arrest is predominantly triggered at the G1 phase through the activation of one or both of the p53/p21^WAF1/CIP1^ and p16^INK4A^/pRB tumor suppressor pathways [126]. For instance, when Plasminogen Activator Inhibitor-1 (PAI-1), a p53 target gene that contributes to tumor suppression, is suppressed by RNAi, both early embryonic mouse fibroblasts and early human BJ fibroblasts can escape the state of replicative senescence [127,128]. SASP factors such as IL-6 and C-X-C chemokine receptor type 2 (CXCR2) activate p53 inducing cell cycle arrest and thereby suppressing tumor cell growth and proliferation [129,130,131]. p21 is a type of cyclin-dependent kinase (CDK) inhibitor that functions in the G1 cell cycle arrest process. It is a transcriptional target of *TP53*, a tumor suppressor gene that plays a pivotal role in regulating the cell cycle [132]. The nuclear form of p21 has been shown to possess tumor-suppressing functions, while the cytoplasmic form has been demonstrated to promote tumor development [133]. Among these, nuclear form of p21, responsible for tumor suppression, is one of the CDK inhibitors that suppress cell proliferation, thereby enabling tumor suppression through its activation [134].

The protein known as p16 has been observed to bind to a specific protein, cyclin, and to regulate the phosphorylation level of another protein, RB. The RB protein family, which includes Rb1, p107, and p130, has been identified as a critical factor in the process of inducing senescence and suppressing tumors. These proteins undergo a process known as phosphorylation, catalyzed by CDKs [135]. This process has the effect of reducing the ability of the RB protein family proteins to inhibit the activity of the E2F family of transcription factors. These factors are essential for the progression of the cell cycle [136]. Increased p16 expression has been observed to be consistently associated with SASP expression, suggesting a potential link between these phenomena. This association may extend beyond the context of aging induction, potentially extending to tumor suppression in conjunction with the RB protein family [137,138].

SASP can suppress tumors not only by maintaining cell cycle arrest but also through immune surveillance. SnCs can produce SASP that modulates the microenvironment of surrounding tissues, particularly affecting adjacent normal endothelial cells to mediate NF-κB signaling. These factors activate the cluster of differentiation 4 (CD4)+ T cells via STAT1 and inducible co-stimulator/ligand signaling, recruiting surrounding T cells to induce immune-mediated surveillance and senescent cell clearance [139]. Ruscetti, M. et al. provided evidence that the suppression of tumors can be achieved via SASP in a mouse model of pancreatic ductal adenocarcinoma. The present study induced tumor cell senescence via MAPK/extracellular signal-regulated kinase (ERK) and CDK4/6 inhibition, thereby activating SASP. The activated SASP induced T cell infiltration around the tumor, enhancing the immune response and confirming tumor suppression [140]. Similarly, aged hematopoietic stem cells (HSCs) were found to induce immune-mediated fibrosis by attracting the natural killer (NK) cells and other immune cells to fibrotic lesions, thereby limiting fibrosis [141]. Furthermore, recent studies reveal that tumor cell senescence can shift from immune evasion to immune surveillance. This occurs as Interferon-γ (IFN-γ) synergizes with SASP to induce tumor cell rejection through enhanced antigen presentation and immune surveillance [142].

#### 2.2.3. Senescent Cell Clearance Mechanisms

T cells, a type of lymphocyte, are characterized by their ability to express a high-affinity T-cell receptor (TCR) that is specific to a single epitope. For activation, the recognition of naturally processed antigens presented by antigen-presenting cells is essential, as is the maintenance of memory capacity as part of adaptive immunity. T cells comprise two major subsets: CD4^+^ T cells (Th1, Th2), which regulate immune responses, and the cluster of differentiation 8 (CD8)+ T cells, which trigger cell death, exhibiting functional parallels with NK cells [143]. CD4^+^ T cells play a key role in halting tumor development by eradicating precancerous, oncogene-induced senescent hepatocytes through immunological mechanisms [144]. For instance, the presence of senescent fibroblasts in aged skin has been linked to an influx of CD4^+^ T cells. CD4^+^ T cells have been shown to eliminate senescent fibroblasts by targeting human leukocyte antigen class II (HLA-II) and human cytomegalovirus glycoprotein B (HCMV-gB), which are secreted by senescent skin cells [145]. CD8^+^ T cells have been identified as playing a vital role in the surveillance of SnCs in the context of aging and cancer [146]. The activation of cytotoxic CD8^+^ T cell immunity by SnCs occurs through mechanisms involving the secretion of pro-inflammatory cytokines and intrinsic antigen presentation [147]. For example, type I IFN signaling has been shown to promote positive regulation of the major histocompatibility complex I (MHC-I) antigen processing machinery in SnCs. The process of aging was accompanied by the recognition of senescence-associated antigens by the immune system, leading to an augmentation in the capacity of CD8^+^ T cells to eliminate SnCs [148].

### 2.3. Telomere Dysfunction

Telomeres are repetitive DNA sequences located at the ends of chromosomes. They are composed of DNA ends and the protective protein complex shelterin [149]. During the process of cell division, telomeres undergo a reduction in length. Telomerase, telomerase reverse transcriptase (TERT) and telomerase RNA component (TERC)) facilitates the maintenance of telomere length by using an RNA template to replicate the repetitive sequences, thereby extending cell lifespan [150].

Telomere shortening is a progressive and inherent aspect of the typical cellular lifespan. In the event of continuous division, the telomere region that serves to protect the chromosome ends can reach a critical length. At this point, the shelterin complex is unable to protect the ends, which results in the activation of the DNA damage response [150]. The activated DNA damage response has been shown to induce cellular senescence, which in turn reproduces the hallmarks of age-related diseases [9]. A recent human study observed age-related telomere shortening in 21 out of 24 tissues examined [151]. Moreover, complications with terminal replication during cell division can be exacerbated by oxidative stress. The process of accelerating telomere shortening ultimately leads to the exposure of telomere ends. This, in turn, activates the ataxia-telangiectasia mutated (ATM) and the ATM and Rad3-Related (ATR) kinases via the formation of DNA damage foci [152]. The ATM/ATR pathway has been demonstrated to induce aging through p53 activation [153]. Herbig et al. suggested that telomere damage in aged human cells activates p53 and p21 via ATM, inducing aging through cell cycle arrest at the G1 phase [154].

The capacity of cancer cells to proliferate indefinitely is associated with the reactivation of telomerase and the stabilization of telomere length [155]. Shortened telomeres have been identified as a contributing factor to age-related diseases in humans, including chromosomal instability and cancer [156]. Telomerase is activated in up to 90% of all human tumor cells, whereas it remains epigenetically silenced in most adult somatic cells [157]. The development of cancer is initiated when cells undergo a series of carcinogenic changes, resulting in the evasion of aging and the acquisition of a state of immortalization characterized by shortened telomeres [158].

Telomerase activity has been demonstrated to correlate with telomere length, thus serving as a significant indicator in diagnostic analyses for certain diseases [159]. For instance, it has been reported as a reliable diagnostic marker in pancreatic fluid samples for distinguishing pancreatic ductal adenocarcinoma (PDAC) from pancreatitis [160]. Additionally, an association has been confirmed between elevated survivin expression levels in the serum of pancreatic cancer patients and reduced survival rates. Elevated levels of survivin expression or increased serum levels result in increased telomerase activity through the process of transcriptional activation of human TERT [161]. Consequently, the assessment of serum survivin concentration or the identification of its expression in tissues has the potential to serve as a clinical tool for identifying patients who are at elevated risk for progression to PDAC [162].

### 2.4. Chronic Inflammation

Inflammation increases progressively during the aging process, a phenomenon termed “inflammaging”. Inflammaging has been demonstrated to be a contributing factor to the development of various age-related diseases, including neuroinflammation, atherosclerosis, osteoarthritis, and sarcopenia [163]. The process of inflammation exhibits a marked increase resulting from a combination of damage at the cellular, molecular, and organismal levels, which itself is an outcome of other significant hallmarks of aging [164].

A distinguishing feature of aged HSCs is an elevated propensity for myeloid/megakaryocytic differentiation [165]. A variety of pro-inflammatory cytokines and growth factors have been identified, including granulocyte colony-stimulating factor (G-CSF), macrophage colony-stimulating factor (M-CSF), granulocyte-macrophage colony-stimulating factor (GM-CSF), IL-1, IL-3, IL-6, and tumor necrosis factor-α (TNF-α). It has been reported that IFNs and fms-like tyrosine kinase 3 (Flt3) ligands promote the differentiation of hematopoietic stem/progenitor cells toward myeloid cells rather than lymphoid cells, thereby inducing an imbalance between myelopoiesis and lymphopoiesis [166,167,168].

Meanwhile, CXCR 4 binds to the C-X-C motif chemokine ligand (CXCL) 12, acting as a key mediator in various physiological and pathological processes, including immune inflammatory responses, hematopoietic regulation, angiogenesis induction, and tumor invasion and metastasis [169]. In the presence of inflammation, HSCs transition from an energy metabolism centered on anaerobic glycolysis to an oxidative respiration. Increased endogenous ROS via oxidative respiration accelerates inflammaging through p38 MAPK activation, increased DNA double-strand breaks, and enhanced apoptosis [170].

Dysregulation of the nutrient-sensing mechanism has also been demonstrated to exacerbate inflammaging. An increased activation of the growth hormone (GH)/insulin-like growth factor 1 (IGF-1)/phosphatidylinositol 3-kinase (PI3K)/protein kinase B (AKT)/mammalian target of rapamycin complex 1 (mTORC1) pathway serves as a prime example of this phenomenon, as it disrupts the regulation of nutrient sensing, which is an antagonistic hallmark of aging [20]. Moreover, the synthesis of proinflammatory cytokines specific to the SASP increases with the accumulation of cellular aging, thus exacerbating chronic inflammation. Hematopoietic and lymphoid progenitor cells decrease, weakening immune responses targeting new antigens with aging. Changes in T cell populations can result in three major outcomes: the amplification of pro-inflammatory T-helper (Th)1 and Th17 cells; the diminished immune surveillance function; and an increased incidence of autoimmune diseases [171].

These mechanisms similarly operate in chronic inflammation associated with cancer. Inflammatory cells within the TME secrete various factors that promote cancer initiation and progression, including proliferation, genetic instability, angiogenesis, metabolic reprogramming, invasion and metastasis, extracellular matrix (ECM) remodeling, cancer stem cell maintenance, and immune surveillance evasion [172]. In addition, strategies to modulate inflammation are being investigated as a potential cancer therapeutic modality. A range of approaches have been explored, including the obstruction of tumor-associated inflammatory cells or the suppression of their infiltration into tumors. Additionally, the pharmacologic inhibition of cytokines such as IL-1β has been investigated as a therapeutic strategy [173]. For instance, studies have demonstrated that IL-1β inhibition suppresses the progression of non-small cell lung cancer (NSLC) in elderly patients, suggesting a potential for combination with immunotherapy and chemotherapy [174].

Inflammation is a dual phenomenon within the TME. Initially, it plays a protective role by aiding in the removal of damaged cells and tissues. However, the process of chronic inflammation is linked to the promotion of cell growth, angiogenesis, and metastasis, thereby driving tumor formation. The TME is comprised of various cell types, including cancer cells, immune cells, fibroblasts, endothelial cells, ECM, and soluble factors [175].

### 2.5. Autophagy Dysregulation

Autophagy is a complex catabolic process that is a fundamental requirement for maintaining cellular homeostasis. Autophagy is essential for maintaining cellular health and contributes to extending lifespan and delaying aging [176]. The function of autophagy exhibits a decline during the typical aging process, resulting in the accumulation of harmful substances and cellular damage. Autophagy dysregulation have been the focus of extensive research in a variety of physiological and pathological conditions, including aging and cancer [177]. This finding demonstrates the necessity of comprehending the context-dependent characteristics of the autophagy process [178,179,180].

Autophagy, a process that facilitates cellular quality control and metabolic adaptation to stress, has emerged as a pivotal focus of numerous studies examining the regulation of aging phenotypes [176]. Aging-induced autophagy dysregulation is regulated through (1) reduced autophagy due to altered signaling pathways [181], (2) defects in autophagosome formation [182], (3) impaired lysosomal function [183], and (4) alterations in selective autophagy and mitophagy [184].

Autophagy has been linked to mTOR activity, and the process of aging may be associated with this pathway. Under nutrient-rich conditions, mTOR not only suppresses the early stages of autophagy but also inhibits its late stages by phosphorylating the UV radiation resistance associated gene (UVRAG) to promote Rubicon binding, thereby suppressing UVRAG-mediated autophagosome maturation [185]. However, aging has been observed to exert an influence on the pathways responsible for sensing nutrients and energy, thereby inducing alterations in mTOR. These alterations, in turn, have been shown to contribute to a decline in autophagy by diminishing the initiation phase of the process [177].

Age-related alterations in the autophagy flux may also stem from functional changes in components of the autophagy-lysosome pathway. For instance, a reduction in autophagy body formation rate was reported in mouse sensory neurons in an age-dependent manner, manifested as delayed recruitment of LC3B to autophagy-related protein 13 (ATG13)-positive phagophore precursor structures. Overexpression of phosphoryl-resistant WIPI2B restored this autophagosome formation defect, suggesting that age-related changes in phosphorylation regulation may inhibit autophagy in post-mitotic cells [182].

Lysosomal function is traditionally known to decline with age, suggesting that aging is associated with the final stages of autophagy. Recent studies have demonstrated a reduction in lysosomal acidification and protease activity in aged cultured primary neurons. This reduction has been shown to affect decreased v-ATPase subunit expression in aged mouse brains [186].

While aging has been demonstrated to affect cellular quality control via autophagy, it is also known to be associated with changes in selective autophagy, particularly mitophagy, the autophagy of mitochondria [187]. This induces morphological changes by regulating mitochondrial fusion controlled by dynamin-related protein 1 (DRP1) and mitochondrial fission controlled by mitofusin (MFN) 1, MFN2, and optic atrophy 1 (OPA1) [188,189]. Mitochondrial fission is a crucial process for segregating damaged mitochondria. Conversely, impaired mitophagy due to aging is incapable of eliminating damaged mitochondria, resulting in an accumulation of ROS and subsequent induction of cellular senescence [190]. Impaired mitophagy has been reported as a mediator of aging phenotypes, including the induction of cellular senescence [191].

The function of autophagy in cancer is complex and multifactorial, exhibiting variations based on cancer type, stage, and genetic profile [192]. In early-stage cancer, autophagy functions as a protective mechanism by mitigating genetic instability and reducing carcinogenic potential through the removal of damaged organelles, misfolded proteins, and ROS [193]. However, in advanced stages, tumor cells use autophagy as a stress adaptation and means to meet high metabolic demands for rapid growth and survival [194]. Impaired autophagy function has been demonstrated to be closely associated with tumorigenesis and malignant transformation. In particular, among the various autophagy-related genes, the function of Beclin-1 has been demonstrated to induce spontaneous tumor formation. Allele loss of Beclin-1 has been detected in 40–75% of various cancers, including prostate, ovarian, and breast cancers [195,196]. This finding suggests that Beclin-1 may perform tumor suppressor. Furthermore, Beclin-1, a key protein initiating autophagy, is regulated by proteins such as Bax-interacting factor-1 (Bif-1) and UVRAG. It has been demonstrated that this process promotes autophagy by forming a complex with the highly conserved phosphatidylinositol 3-kinase VPS34 domain [197,198]. The tumor suppressor protein phosphatase and tensin homolog (PTEN) has been shown to promote autophagy by inhibiting the AKT survival pathway through phosphoinositide phosphatase activity. The disruption of essential components within the PI3K/AKT signaling pathway exerts a substantial influence on tumorigenesis and autophagy dysfunction [199]. Autophagy inhibition has been demonstrated to result in the accumulation of p62 aggregates, which in turn can induce DNA damage, oxidative stress, and cellular toxicity [200]. Impaired autophagy function has been observed to be consistently associated with poor prognosis in various cancers, and the presence of PTEN deficiency or PI3K/AKT signaling abnormalities has been shown to reduce autophagy, thereby promoting tumorigenesis [201].

In established cancer, autophagy serves to address the elevated metabolic demands characteristic of rapidly proliferating cancer cells. It has been demonstrated that this process protects tumor cells from metabolic stress-induced necrosis through macromolecular degradation and helps maintain high metabolic states by recycling essential components [202]. Furthermore, in hypoxic environments, both hypoxia-inducible factor (HIF)-1-dependent and -independent autophagy are promoted, enhancing tumor cell survival under hypoxic conditions [203]. Thus, autophagy could serve as a biomarker for cancer treatment. For instance, the deletion of the autophagy-related gene FAK family kinase-interacting protein of 200 kDa (FIP200) in mouse models led to a substantial suppression of breast tumor growth. Research in pancreatic cancer xenografts and mouse models demonstrated that genetic or pharmacological inhibition of autophagy resulted in significant tumor regression [202,204].

## 3. Distinct Pathways: Where Aging and Cancer Diverge

**Figure 3:**
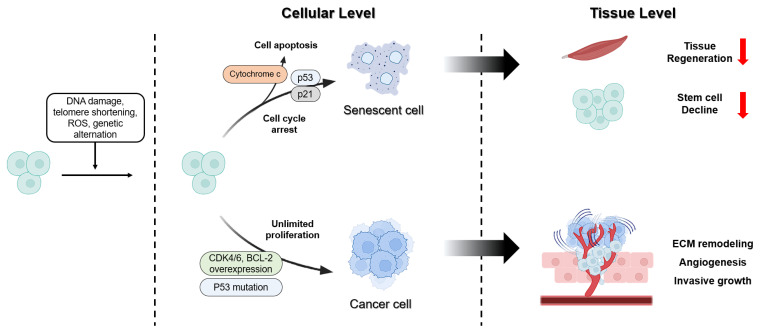
Distinct pathway of cancer and aging in cellular level and tissue level. Cellular stress responses are induced by DNA damage, telomere shortening, ROS, and genetic alterations. In response to these stimuli, some cells undergo cell cycle arrest and apoptosis via the p53/p21 pathway, resulting in the formation of SnCs. Conversely, alterations such as p53 mutations and the overexpression of CDK4/6 and BCL-2 promote unlimited proliferation and drive the transition toward cancer cells. These dis-tinct cellular outcomes result in different tissue level. The accumulation of SnCs reduces tissue re-generation and stem cell decline. In contrast, the proliferation of cancer cells promotes ECM re-modeling, angiogenesis, and invasive growth, thereby enhancing cancer progression.

Although aging and cancer share common mechanisms, such as genomic instability, chronic inflammation, and metabolic changes, they affect the fate of cells and tissues differently. Aging involves suppressing the proliferation of damaged cells and maintaining homeostasis. In contrast, cancer evades these inhibitory mechanisms, inducing unlimited proliferation and tissue destruction [205,206]. This section addresses the divergence points between aging and cancer, from the cellular to the tissue level (Figure 3).

### 3.1. Cellular Fate Decisions

Cells do not divide indefinitely; they cease dividing once they reach a certain number of divisions. As they approach this limit, their division rate slows and they enter a quiescent state, halting division [207]. The process of aging is primarily induced by three distinct pathways. The initial damage to DNA and subsequent shortening of telomeres serves as crucial catalysts for the activation of the DNA damage response. This response prompts the relocation of the p53 transcription factor into the nucleus, where it facilitates the transcription of the cyclin-dependent kinase inhibitor 1A (CDKN1A) gene. This results in the production of p21 [206]. p21 binds to specific CDK proteins, preventing them from binding to cyclin proteins, and ultimately inducing cell cycle arrest. Thereafter, the cell cycle is arrested due to the presence of ROS. Increased internal ROS production activates the p38/MAPK signaling pathway, promoting TP53/p53 transcription. This upregulation of TP53/p53 transcription subsequently induces p21 and CDK activity, resulting in the arrest of the cell cycle [208]. Finally, the cell cycle is arrested through age-related CDKN2A depression. CDKN2A depression activates alternative splicing of CDKN2A mRNA, generating ARF tumor suppressor and p16 proteins. The resulting ARF activates the preceding p53 pathway, inducing p21 expression to arrest the cell cycle. Concurrently, p16 directly binds to specific CDK proteins, forming a complex that inhibits CDK-cyclin complex formation, thereby inducing cell cycle arrest [209].

Cancer cells possess the destiny of unlimited proliferation, unlike SnCs which undergo limited proliferation. Cancer forms through pathways involving the tumor suppressor protein p53, encoded by the p53 gene, and the tumor suppressor protein RB [210]. Both pathways induce arrest at checkpoints in the cell cycle [211,212]. However, when problems occur, such as p53 mutations or loss of the RB pathway, these checkpoints fail to function, allowing growth signals to be ignored. This can lead to cells becoming cancerous [213]. The disruption of cell arrest is a consequence of CDK4/6 expression. In cases of excessive CDK4/6 activation, the G1/S transition accelerates, causing cells entering the S phase prematurely. This occurs before the completion of DNA damage repair or replication stress checkpoint responses. This process can result in the accumulation of replication errors and DNA breaks. Furthermore, CDK4/6 has been shown to phosphorylate non-canonical substrates, thereby disrupting mitotic spindle assembly and increasing the risk of chromosome mis-separation [214,215].

When cells sustain damage from factors such as DNA damage, oxidative stress, and telomere shortening that is reparable, they induce cell cycle arrest. However, when the extent of cellular damage is irreparable, the cell undergoes cell death, such as apoptosis or necrosis [216,217]. Cell cycle arrest or apoptosis functions as a tumor suppression mechanism by halting the proliferation or eliminating damaged cells. The process of apoptosis can occur via two distinct pathways: the intrinsic and the extrinsic pathways [218]. The intrinsic pathway is induced by various stimuli such as oxidative stress, DNA damage, and kinase inhibition, and primarily involves mitochondria and the apoptosome. When apoptosis is induced, mitochondria release cytochrome c into the intermembrane space. Cytochrome c binds to the apoptotic peptidase activating factor 1 (Apaf-1) and procaspase-9 in the cytoplasm, forming a multiprotein complex called the apoptosome. This complex activates caspase-9 via a proximity-mediated mechanism in the presence of ATP or dATP. Activated caspase-9 then activates caspase-3, caspase-6, and caspase-7 [219]. The extrinsic pathway is initiated by the binding of Fas ligand to its receptor, thereby inducing apoptosis. Fas ligand binding to the Fas receptor (CD95) results in the trimerization of the Fas receptor. The cytoplasmic tail of the receptor has been observed to recruit FADD (Fas-associated protein with death domain) through interaction between the death domain of FADD and the Fas receptor [219]. Subsequently, FADD assembles the procaspase-8 molecule, and caspase-8 is activated via proximity-mediated activation. Activated caspase-8 can then activate caspase-3 and -7, which in turn initiate apoptosis [220].

However, in cancer, this process does not exert sufficient effects to kill cells through apoptosis. Cancer cells evade apoptosis through survival strategies, enabling continued proliferation [221]. The p53 mutation, a cause of cancer cell proliferation, is known to be a mechanism for evading apoptosis [222]. Additionally, in cancer, overexpression of B-cell lymphoma-2 (BCL-2) and BCL-XL inhibits apoptosis by blocking the mitochondrial regulator pathway, thereby increasing survival rates [223,224,225]. Furthermore, increased expression of inhibitors of apoptosis proteins suppresses caspase activity, thereby evading apoptosis.

The process of aging is characterized by a gradual decrease in the body’s ability to repair and replace itself. This decline is attributed to cell cycle arrest and a decrease in proliferative stem cells (SCs), which collectively result in a weakened capacity to maintain tissue integrity and function. However, it has been observed that tissue structure can maintain relative homeostasis until the terminal degenerative stage [226]. In contrast, cancer destroys normal tissue structure through invasive growth, angiogenesis, and ECM remodeling driven by unlimited proliferation, causing loss of normal function and replacement by cancerous tissue [221].

### 3.2. Tissue-Level Manifestations

The effects of aging manifest changes not only in cells but also in tissues. Aging is the most significant factor in age-related diseases such as neurodegenerative diseases, cardiovascular diseases (CVDs), and metabolic diseases [227]. These diseases can result in a loss of normal function, disability, or death in severe cases [228]. Aging is associated with a progressive decline in physiological functionality, which arises from diminished tissue regenerative capacity and SC depletion. This process has been shown to lead to a reduction in immune system function and an enhancement in inflammatory responses [229]. For instance, the process of aging is associated with a series of changes in the structure and function of skeletal muscle tissue. These changes are the result of a reduced energy metabolism rate and they lead to a loss of muscle function. In healthy, well-nourished individuals, this process typically progresses slowly. Among the numerous changes associated with aging, the reduction in muscle mass and function is a well-known and significant change, termed sarcopenia [230,231]. A notable finding is that heterochronic parabiosis can restore the proliferation and regenerative capacity of SCs in aged mice, suggesting that extrinsic factors in the blood, such as sex hormones, α-Klotho, and fibroblast growth factor (FGF), can reverse SC aging [232]. Kim et al. reported that SC depletion in induced paired box 7 (Pax7) knockout mice induces neuromuscular junction degeneration at a young age and exacerbates muscle fiber atrophy following neuromuscular deterioration [233]. The process of aging has been demonstrated to induce neurodegenerative diseases through the formation of misfolded proteins and reduced neuronal function. For instance, the process of aging is associated with the aberrant accumulation of amyloid β-proteins (Aβ) within neurons, resulting in the formation of senile plaques due to their progressive loss [234]. Aβ self-aggregates accumulate on neuronal membranes, generating ROS and causing membrane lipid peroxidation, which produces 4-hydroxy-2-nonenal. This results in impaired function of membrane ion pumps (ATPases), glucose and glutamate transporters, and disruption of neuronal Ca^2+^ homeostasis. Consequently, this induces neuronal hyperexcitability, excitotoxicity susceptibility, and metabolic depletion, ultimately leading to Aβ neurotoxicity [235,236]. Moreover, an increase in misfolded proteins and tau protein aggregation has been observed to induce Aβ oligomerization, which may contribute to the development of Alzheimer’s disease (AD) by impeding synaptic plasticity and signaling. The aggregation of tau protein can also result in the formation of neurofibrillary tangles. These phenomena progressively accumulate in the brain and can cause AD [237].

In cancer, cells evade cell cycle arrest by inhibiting the p53 and RB pathways, thereby repeating cell proliferation. This phenomenon can be considered a characteristic of cancer cells that survive beyond their normal lifespan [238]. The process by which normal cells transform into cancer cells occurs not only during early carcinogenesis but also during tumor metastasis. Cancer development manifests through multiple stages. Changes in signaling pathways due to epigenetic alterations—caused by mutations in tumor suppressor genes and oncogenes, DNA methylation (DNAm), or histone modifications—lead to cells becoming resistant to regulation by growth factors and hormones. This enables them to survive independently, resulting in malignant transformation [239,240,241]. Additionally, malignant transformation occurs due to decreased E-cadherin expression. This phenomenon is associated with changes in cellular phenotype, including EMT and cell migration [242]. Through metabolic reprogramming, cells shift their energy production and metabolic pathways, transitioning to a metabolic state prioritizing growth and proliferation [243]. Despite sufficient oxygen availability, cells maintain glycolysis-dependent pathways via the Warburg effect instead of mitochondrial oxidative phosphorylation [244]. This creates a hypoxic environment within the tumor, activating HIF-1α, promoting the survival and growth of tissue SCs, and even inducing angiogenesis [245].

## 4. Biomarkers in Aging and Cancer

**Figure 4:**
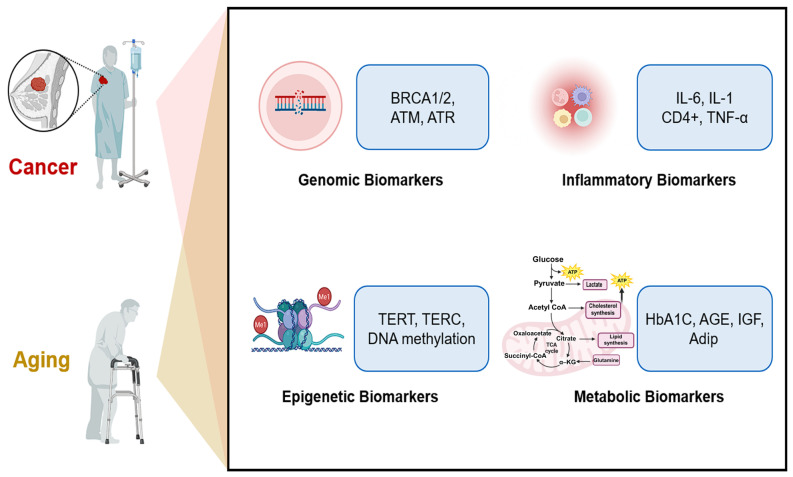
Biomarkers in aging and cancer. Aging and cancer are interconnected through various mechanisms, including genomic instability, chronic inflammation, epigenetic alterations, and metabolic dysregulation. Along these pathways, specific biomarkers are generated—genomic biomarkers (e.g., BRCA 1/2, ATM, and ATR), inflammatory biomarkers (e.g., IL-6, IL-1, and TNF-α), epigenetic biomarkers (e.g., TERT, TERC, and DNAm), and metabolic biomarkers (e.g., HbA1C, AGE, and IGF).

Aging and cancer share common biological pathways as well as distinct ones. Along these pathways, various biomarkers are generated, which can serve as potential tools for early diagnosis (Figure 4).

### 4.1. Genomic Biomarkers

The genomes of organisms are continuously exposed to diverse external environments and endogenous stress, leading to molecular-level changes that induce aging and cancer [39,246]. Furthermore, many cancers exhibit genomic instability, which can lead to the loss or gain of large chromosomal regions. This instability can also result in base-level substitutions, insertions, and deletions, which can ultimately induce malignant transformation in cancer cells [247]. Ultimately, the continuous accumulation of genomic damage increases the incidence of age-related diseases and cancer [248].

Recent findings have identified mutations driven by genomic instability as a critical enabling characteristic that facilitates cancer development in most aging and cancers [39,246]. For example, mouse models with DNA repair defects exhibited an aged vascular phenotype. In smooth muscle cell-selective ERCC1 Knockout (SMC-KO) mice, the loss of the DNA repair protein ERCC1 in vascular smooth muscle cells (VSMCs) acts as a key aging factor. ERCC1 deficiency causes DNA damage accumulation, promoting increased p16 and p21 expression and inflammatory responses. These accelerated non-atherosclerotic vascular aging in SMC-KO mice [249]. Administration of the phosphodiesterase 1 (PDE1) inhibitor ITI-214 showed temporary improvement in vascular function [249,250]. In human, DNA repair defects are the underlying cause of progeria syndromes [251]. In contrast, breast epithelial tissue from women with germline mutations in the BRCA1 or BRCA2 genes exhibits DNA repair defects, leading to an elevated risk of breast and ovarian cancer, as well as premature aging [252]. Both ATM and ATR are indispensable kinases in the DNA damage response, each with specific roles in sensing and responding to different types of DNA lesions [253]. ATM primarily responds to DNA double-strand breaks, while ATR is crucial for replication stress and ssDNA. Their proper function is vital for genomic stability, development, and cancer prevention, and their inhibition or deletion can lead to severe developmental defects and genomic instability [253].

Beyond nuclear DNA, mitochondrial DNA (mtDNA) can also undergo mutations and deletions due to external or internal stressors, potentially contributing to aging and cancer [254]. Mouse models with mtDNA mutations exhibit premature aging phenomena such as osteoporosis, muscle atrophy, and respiratory disorders [255]. In mouse with mtDNA damage due to DNA polymerase γ deficiency, aging and shortened lifespan were observed [256]. Furthermore, human diseases associated with mtDNA damage also exhibit aging-like phenotypes, suggesting that mtDNA mutations could serve as biomarkers for aging [257].

A multitude of studies provide evidence that the process of aging and the development of cancer are associated with genomic instability. Additionally, interventions that target the reduction in DNA damage and the enhancement of repair processes may potentially postpone the onset of cancer, including age-related diseases.

A promising study has been reported that systematically investigated the genetic architecture of brain aging and identified potential drug targets and repurposed drugs capable of extending healthy lifespan, primarily utilizing brain aging gap (BAG) as a core biomarker. This study utilized MRI data from a large cohort of healthy individuals in UK Biobank and established a brain age model and systematically identified genes that could serve as therapeutic and prognostic markers for aging and related diseases such as AD, using the brain age gap (BAG) as an indicator [258]. The BAG is the difference between the predicted age from MRI data and chronological age and is considered a promising indicator of brain health [258]. A positive BAG value indicates accelerated brain aging, whereas a negative BAG value indicates slowed brain aging [258]. The subjects with brain disorders such as AD, dementia, and schizophrenia have higher BAG values than healthy subjects [258]. Identified genetic factors responsible for BAG offer insights into the genetic basis of brain aging and potential avenues for drug development to promote healthy aging [258]. Seven key genes and their relationships with BAP are summarized in Table 1 and genomic biomarkers are summarized in Table 2.

### 4.2. Epigenetic Biomarkers

Epigenetic changes influence gene expression and key cellular functions, contributing to aging and cancer development. These changes include alterations in DNAm patterns, post-translational modifications of histones, and non-coding RNAs (ncRNAs), and are recognized as markers of aging and cancer [259].

DNAm in aging generally exhibits hypomethylation across genes. In contrast, many cancers show the opposite pattern of hypermethylation. Cancer has been observed to manifest epigenetic characteristics, such as the reprogramming of DNAm patterns, including the phenomenon of hypermethylation [260,261]. Thus, epimutations—epigenetic changes—primarily occur in intronic or intergenic regions, driving aging and cancer, but also arise through methylation and silencing of key tumor suppressor genes like p16 and p53 [262]. Notably, DNA (cytosine-5)-methyltransferase 3A (DNMT3A), which functions in de novo methylation, and TET methylcytosine dioxygenase 2 (TET2), which initiates demethylation, are frequently mutated in clonal hematopoiesis of indeterminate potential, known as an indicator for aging-related diseases like coronary heart disease and hematological cancers [263,264,265]. Recently, DNMT inhibitors are being studied for treating blood cancers and solid tumors. DNMT inhibitors feature a modified cytosine ring structurally similar to naturally occurring nucleosides. They are incorporated into DNA during replication in place of cytosine, thereby inactivating DNMTs [266,267]. For example, first-generation DNMT inhibitors include 5-azacytidine and 5-cytidine-2’-decitabine are first-generation DNMT inhibitors. Nucleoside analogs overcoming these limitations were subsequently developed [268]. Guadecitabine (SGI-110) is an improved second-generation form. Clinical studies for solid tumor treatment are underway using combination therapy with immunotherapy and anticancer agents [269,270,271]. Current studies are focused on the lysine (K)-specific demethylase (KDM) family, with a particular emphasis on exploring cancer treatments that involve the inhibition of KDM activity through the use of small molecules such as Iadademstat [272,273]. Promoter methylation is a significant regulatory element for TERT expression, correlating with both TERT mRNA levels and telomerase activity [274]. While the TERT promoter region around the transcription start site typically unmethylated in actively transcribed TERT, hypermethylation of the TERT gene has been shown to correlate with telomerase activity in various cancers [275]. The downregulation of TERT expression via epigenetic modifications such as TERT promoter methylation can significantly influence clinical study design [276].

In aged cells and cancerous tissues, a variety of post-translational modifications of histones, including acetylation, methylation, and phosphorylation, have been observed. These modifications arise in the N-terminal region of histones, leading to abnormalities in DNA binding and dynamically altering chromatin structure, which can disrupt gene transcription, metabolic regulation, and the maintenance of cellular homeostasis [277].

Histone methylation is the process of transferring a methyl group from S-adenosyl methionine to lysine or arginine residues. This process is catalyzed by lysine methyltransferases and arginine methyltransferases. Recent studies have indicated that histone methylation marks, including H3K4me3, H3K27me3, and H3K36me3, demonstrated significant alterations during the aging process [278]. These methylation abnormalities exhibit tissue-specific patterns in aging-related diseases. For example, Sun et al. has found that the level of H3K4me3 is increased in the HSCs of aging mice [279]. In neurodegenerative diseases such as AD, neurons in the prefrontal cortex show a significant increase in H3K9me2 and a decrease in H3K27me3, which leads to the silencing of genes associated with synaptic plasticity [280]. Similarly, in muscle atrophy, aging mouse muscles show elevated levels of H3K27me3, which inhibits the differentiation of muscle stem cells and muscle regeneration [281]. H3K36 methylation in the intestinal epithelium has several practical implications, particularly in understanding and potentially treating diseases related to cell plasticity, such as cancer, and in advancing regenerative medicine [282].

Histone acetylation and deacetylation are processes that add or remove acetyl groups, respectively, from lysine residues protruding from nucleosomes. These processes are closely associated with major cellular functions, including DNA replication, DNA damage repair, and RNA transcription. These reactions are primarily catalyzed by histone acetyltransferases (HATs) or histone deacetylases [283]. Qui et al. revealed that the expression and catalytic activity of major HATs, which is p300/CBP, decrease with aging and their nuclear localization also undergoes alterations [284]. In contrast, reducing p300/CBP, enzymes catalyzing H3K27ac, lowered homeostatic amyloid-reducing genes’ expression and increased secretion of toxic Aβ(1–42) in iPSC-derived neurons from familial AD patients carrying an amyloid precursor protein duplication. These results suggest that H3K27ac-driven transcriptional programs act as compensatory mechanisms to counteract APP-related pathology, indicating a protective role of H3K27ac in mitigating AD progression [285].

Histone phosphorylation is a process that adds negative charges to the side chains of serine, threonine, and tyrosine residues. This modification of the proteins affects the structure of the chromosomes, allowing them to interact with transcription factors. In turn, these factors regulate the expression of genes that are involved in the cell cycle and proliferation [286]. The dysregulation of histone phosphorylation, characterized by the abnormal accumulation of DNA damage-related phosphorylation markers and reduced efficiency of signal-responsive phosphorylation events, exhibits substantial reprogramming properties, emerging as a biomarker of aging. In aged cells, there is a marked increase in basal γ-H2AX levels, indicating heightened genomic instability [287]. Another phenomenon is a reduced ability to form new γ-H2AX foci upon damage, reflecting diminished repair efficiency [288]. The MAPK and Aurora kinase pathways have been identified as critical regulators of aging-associated phosphorylation processes. p38-MAPK has been observed to remain constitutively activated in aged cells, a process that has been shown to promote NF-κB influx and induce SASP production [289]. In contrast, Aurora B kinase activity exhibits a decline with aging, resulting in chromosome segregation errors and karyotype instability [290].

Mutations in chromatin remodeling complexes predict cancer aggressiveness. Various cancers frequently contain mutations in components of the ATP-dependent SWI/SNF chromatin remodeling complex (SWI/SNF complex) [291]. These mutations are found in approximately 20–25% of all human cancers and impact the complex’s ability to regulate gene expression by altering how DNA is packaged [292]. In PTEN-deficient cells, BRG1 (SMARCA4) regulates c-Myc and MAPK signaling, and stabilization of BRG1 maintains tumor cell growth [293]. Elevated BRG1 expression in PTEN-deficient prostate cancer (PCa) cells resulted in chromatin remodeling that facilitated a protumorigenic transcriptome, thereby increasing the cells’ dependency on BRG1 [293]. BRG1 inhibitors (e.g., PFI-3) suppress tumor progression in PTEN-deficient preclinical models, suggesting BRG1 is a promising target for these cancers [294].

Non-coding RNAs are characteristic features observed in aging and cancer. Examples include long ncRNAs (lncRNAs) (greater than 200 nt), microRNAs (miRNAs) (~22 nt), small nucleolar RNAs (sno RNAs), and small interfering RNAs (siRNAs) [295]. These elements do not function as templates for protein synthesis. Instead, they exert their influence on post-transcriptional pathways, with implications for both longevity and carcinogenesis [296]. Studies in cell and animal models demonstrating the acquisition of new functions or the loss of existing functions have proven that ncRNAs, including miRNAs, play a causally important role in aging and cancer. For example, miR-21 is found to be upregulated in T cells from older adults (65–85 years) compared to younger individuals, and in replicative senescent endothelial cells [295]. In age-related CVD, miR-21, miR-34a, miR-92a, and miR-146a are upregulated, whereas miR-125b, miR-126a, miR-142, and the miR-30 family are downregulated [295]. miR-455-3p deficiency causes cognitive decline and shortened lifespan in mice [297], while reduced miR-455-3p expression in human cancer cells increases cell proliferation and invasiveness [297]. Conversely, miR-455-3p overexpression protects neural function and extends lifespan, and this miRNA also inhibits tumor growth in a liver cancer transplant model [298]. In the molecular pathogenesis of PCa, miR-21, miR-221, and miR-1290 facilitate tumor proliferation, invasion, and therapeutic resistance, whereas miR-375 exerts tumor-suppressive effects by constraining EMT [296]. lncRNAs such as MALAT1, NEAT1, PCAT-1, and SCHLAP1 promote oncogenic signaling and metastasis, while PCAT-14 functions as a tumor suppressor [296]. The prostate-specific lncRNA PCA3 has been clinically implemented as a diagnostic biomarker [296]. Epigenetic biomarkers are summarized in Table 2.

**Table 2:** Summary of genomic and epigenetic biomarkers in aging and cancer.

Biomarker Class	Representative Molecules	Sample Type	Detection Platform	Evidence Level
Genomic	ERCC1 defect	Mouse VSMC	SMC-KO	accelerated, nonatherosclerotic vascular aging in mouse model [249]
	PDE1	upregulated in aorta	qRT-PCR	Decreased vasodilation function in aging mice [249,250]
	BRCA1, BRCA2,	Normal breast tissue carrying germline mutations (BRCA1, BRCA2)	IF, Flow Cytometry, RNA-seq	Clinical, accelerated biological aging phenotypes [252], increase susceptibility to breast cancer [252],
	ATM, ATR	Mouse Models	knockout, knockin, transgenic mouse models	Preclinical Data [253]
	mtDNA	mtDNA deletions Human tissues (skeletal muscle, brain, colonic crypts); Mouse tissues	NGS, ddPCR	Fundamental Phenotype of aging in mouse, fly, and worm models [254]
	mtDNA	Mouse tissues, mouse cells, blood	Histochemical Analysis, RT PCR, Oxygen Electrode	Respiration defects, development of B-cell lymphoma, in vivo and in vitro [255]
	Primary Mitochondrial Diseases	Blood, Muscle DNA, Uroepithelial cells	NGS, WGS, and RFLP testing	age-related neurogenetic disorders [257]
	Brain Age Gap	MRI data in UK biobank Tissues, blood	MRI, DL	Genetically Supported Druggable Genes, a large cohort [258]
Epigenetic	DNMT3A mutations, TET2 mutations	Blood	Targeted deep exome sequencing (custom panel), Illumina NovaSeq 6000 platform	Clinical, Associated with higher average age and increased risk of CVD [264]
	DNMT1, DNMT3a, DNMT3b	Cancer tissues	DNA methylation assays	Aberrant expression associated with tumor development [266], in vitro, in vivo
	LINE-1, IL22RA1, PRAME, PAX8, GAGE2A, B2M	Blood, Tumor samples	Pyrosequencing, Illumina array	phase 1 dose-escalation study (NCT02998567) [269]
	KDM1A	HCC, Xenografts in Nude Mice	Western blot, flow cytometry qRT-PCR	role of KDM1A in sorafenib resistance of HCC, in vitro, in vivo [273]
	TERT	TERT Hypermethylated Oncological Region	DNA methylation assays, NGS	Human Tumors, cell lines, normal tissue and cells [275]
	H3K4me3; H3K27me3	Aged hematopoietic stem cells (HSCs)	RNA-seq, ChIP-seq	H3K4me3 levels increase in aged HSCs; In vitro, in vivo [279]
	H3K9me2; H3K27me2 Catalyzed by KDM7A	Brain Regions; Cell Lines	Western blot, ChIP-qPCR qRT-PCR	in vitro, in vivo [280].
	H3K27ac (Catalyzed by EP300/CBP)	AD patient brains; iPSC-derived neurons (AD model)	ChIP-seq; RNA-seq, ELISA	In vitro iPSC-neuron model; Comparison to Human Brain Data [285]
	H3K36me3	Mouse small intestine, Intestinal organoids	RNA-seq	in vitro and in vivo models [282]
	SWI/SNF mutations	Tumor tissue samples	CRISPR screening	in vitro and in vivo models [291]
	BRG1 (component of SWI/SNF)	PTEN-deficient PCa cells (PCa model)	ChIP-Seq; RNA-Seq	In vitro, in vivo [293]
	miR-21	Blood, tissue (breast, colorectal, leukemia, lung, prostate	qRT-PCR or sequencing	Diagnostic and prognostic biomarker for CRC; biomarker for CVD [295,296]
	miR-455-3p	Tumor tissues (Osteosarcoma, HCC, Esophageal Squamous Cell Carcinoma, BC)	qRT-PCR	Functions as a tumor suppressor (HCC); potential target for diagnosis and prognosis in Osteosarcoma (OS) [297,298]
	PCA3 (PCa Antigen 3)	urine	Molecular urine analysis	First FDA-approved ncRNA cancer biomarker test; used for diagnosis of PCa [296,299]

Abbreviations: IF; Immunofluorescence, NGS; Next-generation sequencing, ddPCR; Droplet Digital PCR, RT-PCR; Reverse Transcription-PCR, WGS; whole genome sequencing, RFLP; Restriction Fragment Length Polymorphism, qRT-PCR; quantitative real time PCR, ELISA; Enzyme-Linked Immuno-sorbent Assay, ChIP-seq; Chromatin immunoprecipitation sequencing.

Epigenetic changes and aging share a complex relationship, and the epigenetic clock serves as a powerful tool for quantifying an individual’s biological age and rate of aging. The DNAm clock, often referred to as the epigenetic clock, is a powerful tool designed to estimate biological age by analyzing aging-related DNAm changes [300]. By examining genome-wide methylation profiles, these clocks offer predictive insights into mortality and age-related disease risks, effectively differentiating biological age from chronological age and addressing persistent inquiries in gerontology. Beyond blood, DNAm-based age prediction has been studied across diverse tissues and body fluids, with enhanced accuracy observed when integrating data from multiple sources [301]. The performance of DNAm clocks can vary depending on in vitro conditions, and challenges persist in their broad application and interpretation. Continued refinement is essential to establish these clocks as robust biomarkers of aging and functional decline [300]. DNAm clocks can be broadly categorized into first-generation clocks (primarily predicting chronological age) and second-generation clocks (predicting biological age and health-related factors) [302].

The first-generation clocks, including Hannum, Horvath, and Weidner, can predict chronological age more accurately [303]. Hannum et al. introduced apparent methylomic aging rates (AMARs), influenced by sex and genetic variants, highlighting DNAm as both a marker and potential regulator of human aging processes [304]. Horvath’s clock introduced the first multi-tissue DNAm age estimator applicable to all human tissue sources (except sperm) and the entire lifespan (from fetal samples to individuals over 100 years old) [305]. Weidner et al. showed that blood aging can be trackable using DNAm changes at as few as three specific CpG sites within or near the *ITGA2B*, *ASPA*, and *PDE4C* genes, highlighting the precision and efficiency of these markers [306].

While 1st-generation clocks accurately predict chronological age, they lacked insights into biological age measurement and disease prediction, leading to the development of 2nd -generation clocks such as GrimAge and PhenoAge [307,308]. They have proven valuable in predicting disease risk and mortality [309,310,311]. GrimAge, in particular, strongly predicts lifespan and healthspan [312]. PhenoAge is an algorithm that estimates biological age, utilizing nine blood biomarkers, including albumin, creatinine, glucose, C-reactive protein, Lymphocyte percent, mean cell volume, red cell distribution width, alkaline phosphatase, white blood cell count to measure an individual’s physiological health status, and degree of aging [308]. DunedinPACE was designed to quantify the pace of biological aging by analyzing 20 years of longitudinal data (19 indicators of multi-organ system integrity) [313].

While existing clocks simply select CpGs with the strongest correlation to age, CausAge, AdaptAge, and DamAge clocks represent an attempt to interpret the aging process more mechanistically by identifying CpG sites with causal relationships [314]. CausAge is a causality-enriched clock constructed using CpG sites causally related to aging. CausAge was developed using an ElasticNet regression model with 586 CpG sites. AdaptAge builds upon CausAge by being designed to capture CpG sites associated with adaptive responses to aging. DamAge is a clock constructed using only CpG sites associated with damage. The development of AdaptAge and DamAge focuses on enhancing predictive accuracy and interpretability for aging-related phenotypes by separating aging markers into ‘damage’ markers and ‘adaptation’ markers [314]. Meanwhile, traditional epigenetic clocks, trained on bulk tissues, are influenced by age-related changes in immune cell composition [315]. This makes it difficult to distinguish between true cellular aging and shifts in cell type proportions.

The IntrinClock developed by Tomusiak et al. addresses this by being designed to be unaffected by changes across 10 immune cell types, including CD8+ T-cell subsets [315]. This resistance to immune cell compositional changes makes it a more accurate measure of cell-intrinsic aging processes. A blood-based epigenetic clock for intrinsic capacity (IC) was developed [316]. This is a clock specifically trained to predict an individual’s Intrinsic Capacity (IC), a key indicator from the clinical perspective of aging [316]. The IC represents a composite score across five domains: cognition, mobility, psychological well-being, sensory function, and vitality. When analyzed using Framingham Heart Study data, the IC Clock was found to predict all-cause mortality risk more strongly than 1st- and 2nd-generation clocks (HR = 1.38). Furthermore, the CpG sites included in the IC Clock showed little overlap with existing clocks, indicating that it captures unique aspects of aging biology [316].

Jacques et al. has created an “Aging Atlas” mapping aging across 17 human tissues using DNAm analysis of 15,000 samples from adults aged 18–100 [317]. After mapping methylation changes across 900,000 potential locations within DNA, they created an open-access atlas. Most tissues exhibited age-associated hypermethylation, particularly in regions with low methylation in younger individuals, suggesting a coordinated increase in methylation at previously unmethylated sites. The exceptions are skeletal muscle and the lungs, where methylation loss is greater with aging. The overall global mean methylation across the analyzed tissues ranged from 38% to 63% [317]. The retina displayed the highest value, with a 63% average methylation rate, followed by the stomach (57%), heart (53%), muscles (51%), skin (48%), and cervix (35%), which suggests the retina ages fastest, in contrast to the cervix, which ages slowest [318]. The study identified *PCDHGA1* as one of the key disruptors that exacerbates aging signals in various tissues, alongside *MEST*, *HDAC4*, and *HOX* genes, as well as a resilient NAD+ salvage pathway module [317]. The comparison of DNAm clocks is summarized in Table 3.

### 4.3. Inflammatory Biomarkers

An increase in inflammatory levels has been identified as a contributing factor in both the aging process and the development of cancer. In the process of aging, this phenomenon is known as inflammaging, which, when repeatedly accumulated, results in the development of inflammatory aging-related diseases, including osteoarthritis, sarcopenia, and neuroinflammation [319,320]. Similarly, inflammation establishes a tumor-promoting environment and is considered one of the enabling characteristics of cancer [321].

During aging, the sustained secretion of cytokines associated with SASP contributes to the transition toward inflammaging. Among these, IL-6 and IL-1β are the most representative cytokines [96]. IL-6 regulates immune cell differentiation and proliferation, and its elevated systemic levels contribute to chronic inflammatory states that underlie diseases such as sarcopenia, cardiovascular disorders, and insulin resistance. IL-1β, a major component of SASP, is strongly linked to systemic inflammatory responses [99]. In cancer, both IL-6 and IL-1β promote tumor growth and enhance invasiveness. Specifically, IL-6 activates the STAT3 signaling pathway, leading to anti-apoptotic effects and promoting cell proliferation, thereby enabling sustained tumor growth by evading cell death [102]. IL-1β increases invasiveness and fosters a pro-tumorigenic immune microenvironment, thus facilitating carcinogenesis. Another key cytokine involved in both aging and cancer is TNF-α. In aging, TNF-α promotes tissue damage and neuroinflammation, while in cancer, it activates NF-κB signaling, enhancing angiogenesis and metastasis, ultimately creating a microenvironment that supports tumor survival [168].

Tsukamoto et al. demonstrated that in aged environments, impaired Th1 differentiation of CD4^+^ T cells reduces antitumor immunity, which can be restored by IL-6 blockade or deficiency [322]. IL-6 inhibition further promotes CD8+ T cell-dependent tumor elimination through the restoration of CD4+ T cell function in an IFN-γ-dependent manner. Mechanistically, IL-6 has been shown to induce the transcription factor c-Maf expression, leading to increased production of IL-4 and IL-21, which have been observed to suppress Th1 differentiation. Additionally, IL-6 has been found to enhance IL-10 production by CD4+ T cells, thereby dampening CD8+ T cell responses [323]. Mei et al. demonstrated that pathological levels of TNFα, IL-6, and IL-10 suppress erythroid differentiation and impair hematopoiesis by inducing apoptosis through a ROS-mediated caspase-3 pathway [324]. The recent randomized phase II clinical trial, the NORDIC9 study, C-reactive protein (CRP) demonstrated the strongest prognostic value for overall survival of elderly metastatic CRC patients. CRP indicated it could guide decisions regarding palliative chemotherapy in vulnerable patient populations [325]. Chitinase-3-like protein-1 (CHI3L1) or YKL40 is a secreted glycoprotein involved in inflammation, macrophage polarization, apoptosis, and cancer [326]. Its expression increases in various inflammatory and immune diseases. Acting via cytokines like TNF-α and IL-6, CHI3L1 serves as a diagnostic and prognostic marker and a potential therapeutic target in inflammatory diseases. Inflammatory biomarkers are summarized in Table 4.

### 4.4. Metabolomic Biomarkers

In the body, glucose and its oxidative by-products permanently interact with the amino groups of both intra- and extracellular long-lived proteins, DNA, and lipids. This series of Maillard reactions is referred to as non-enzymatic glycosylation or glycation, resulting in the production of a diverse array of compounds termed advanced glycation end-products (AGEs) [327]. Glycation has emerged as a key metabolic hallmark of aging. It progressively alters the structure and function of long-lived macromolecules in skeletal muscle, skin, arteries, and nerves [328,329]. Structural proteins such as collagen, elastin, and myosin undergo glycation, leading to increased stiffness and loss of elasticity—a characteristic similar to age-related tissue degeneration [330]. Among measurable markers, glycated hemoglobin (HbA1c) is widely used as the diagnostic tool for diabetes and prediabetes [331]. Diagnostic thresholds for prediabetes are 5.7–6.4% (39–46 mmol/mol) and for diabetes are ≥6.5% (48 mmol/mol). To increase the diagnostic SN, 2 h post-load glucose measurement from an oral glucose tolerance test (OGTT) has been recommended for use alongside H1bA1c [331]. Several specific AGE molecules are commonly studied as biomarkers due to their prevalence and pathological significance [327]. Nε-Carboxymethyl-lysine (CML) is formed when methylglyoxal reacts with lysine. It is widely used as a general marker of glycation. Nε-(1-carboxyethyl)lysine (CEL) is found in human lens proteins and is a product of chemical modification by methylglyoxal. Glucosepane is identified as the most common AGE found in type I collagen. N2-Carboxyethyl-2’-deoxyguanosine is found in DNA and is considered a potential biomarker for chronic hyperglycemia [327]. AGEs interact with their receptor (RAGE) to induce oxidative stress and inflammation. The soluble RAGE (sRAGE) isoforms include endogenous secretory sRAGE (esRAGE) and cleaved RAGE (cRAGE) [332]. In healthy controls, cRAGE negatively correlated with age, while AGEs/sRAGE and AGEs/cRAGE ratios positively associated with age. An increase in the AGEs/cRAGE ratio was linked to a higher risk of all-cause mortality in T2D patients. sRAGE was associated with the development of Major Adverse Cardiovascular Events (MACE) in T2D patients [332].

In contrast to the metabolic slowing of aging, cancer cells exhibit metabolic reprogramming characterized by accelerated glucose uptake and glycolysis, even in the presence of oxygen—a phenomenon known as the Warburg effect. This metabolic shift fuels rapid proliferation by supplying ATP and biosynthetic intermediates [333]. These characteristics are particularly utilized as indicators for diagnosing and tracking solid tumors [334]. Key glycolytic enzymes such as hexokinase-2 (HK2) are overexpressed in many cancers. Genetic deletion of HK2 significantly reduced tumor burden and average tumor size in mouse models of LC and BC [335]. Zheng et al. demonstrated the efficacy of reducing glycolysis by selectively binding and inhibiting HK2 using the novel compound Benitrobenrazide (BNBZ) in tumor growth xenograft models [336]. The byproduct of enhanced glycolysis, lactate, accumulates in the TME, promoting cancer progression, angiogenesis, and metastasis, while suppressing antitumor immunity [337,338,339,340]. Lactic acid in the blood or within TME may serve as a predictive and prognostic biomarker for cancer [340]. Lactate dehydrogenase A (LDHA), the enzyme responsible for lactate production, is frequently upregulated in tumors [340]. Its genetic or pharmacologic inhibition suppresses KRAS- or epidermal growth factor receptor (EGFR)-mutant tumor growth [341]. Recent strategies using proteolysis-targeting chimeras (PROTACs) to degrade LDHA have shown promise in PC [342].

Aging and cancer are significantly influenced not only by carbohydrates but also by amino acid metabolism. Among various amino acids, changes in glutamine and tryptophan are particularly important indicators in aging and cancer [343,344]. In aging, glutamine levels decrease, inhibiting the synthesis of the antioxidant glutathione (GSH). This promotes oxidative stress and cellular aging within the body, potentially leading to age-related diseases [345]. Cancer exhibits an excessive dependence on glutamine, often termed glutamine addiction. Glutamine acts as a carbon and nitrogen donor, fueling the tricarboxylic acid (TCA) cycle, nucleotide biosynthesis, and amino acid production. Inhibition of glutaminase-1 (GLS1), the enzyme converting glutamine to glutamate, disrupts these anabolic pathways and attenuates tumor growth [346,347]. Accordingly, GLS1 inhibitors are being investigated as targeted anticancer therapeutics that exploit cancer’s metabolic vulnerabilities [348].

Both aging and cancer are characterized by upregulated tryptophan-degrading enzymes, including indoleamine-2,3-dioxygenase (IDO) and tryptophan-2,3-dioxygenase (TDO) [349]. Tryptophan depletion in the body suppresses T cell function or promotes the regulatory T (Treg) differentiation due to increased kynurenine, thereby inhibiting CD8+ T cells and impairing immune function [349]. In aging, chronic activation of this pathway contributes to immunosenescence, while in cancer it establishes an immunosuppressive microenvironment. For example, Du et al. demonstrated anti-glioma and anti-pancreatic efficacy by targeting IDO/TDO with a novel drug to inhibit the tryptophan/kynurenine pathway [350]. Kim et al. similarly demonstrated that targeting IDO and TDO can overcome checkpoint inhibitor resistance and activate CD8+ T cells [351].

The characteristics of aging and cancer also differ in growth hormone metabolism. GH/IGF-1 axis orchestrates cellular proliferation, metabolism, and lifespan regulation. [352]. Age-related decline in GH and IGF-1 signaling correlates with reduced anabolic activity but enhanced longevity in multiple models [353]. Individuals with growth hormone receptor mutations (Laron syndrome) exhibit markedly reduced risks of cancer, stroke, and T2D [354,355]. Preclinical evidence supports that targeted modulation of this pathway may extend healthspan. Administration of an anti-IGF-1R monoclonal antibody (L2-Cmu) in aged mice reduced tumor incidence, suppressed inflammation, and extended lifespan by approximately 9% [356]. Quipildor et al. reported that chronic IGF-1 overexpression improved motor function and mood in male mice but showed sex-specific metabolic responses, highlighting the complex, tissue-dependent regulation of IGF-1 in aging [357]. In cancer, the same GH/IGF-1 axis functions aberrantly, promoting mitogenesis and resistance to apoptosis. Overactivation of IGF-1R signaling contributes to tumor growth, metastasis, and therapeutic resistance. Thus, inhibitors targeting IGF-1R or downstream effectors are being explored as potential anticancer strategies [358,359].

Adiponectin, an adipocyte-derived hormone, enhances lipid oxidation and insulin SN. Its circulating levels decline under metabolically adverse conditions, correlating with increased oxidative stress [360]; conversely higher levels are associated with frailty in the elderly [361]. Choubey et al. demonstrated that declining adiponectin signaling contributes to age-related testicular dysfunction by impairing insulin SN, metabolism, steroidogenesis, and increasing oxidative stress. Furthermore, it showed that exogenous adiponectin treatment can significantly reverse these regressive changes, suggesting its potential as a therapeutic strategy for improving male reproductive health during aging [362].

Cancer cells reprogram lipid metabolism to sustain rapid proliferation [363]. Enhanced de novo fatty acid synthesis, mediated by fatty acid synthase (FASN), produces palmitate, a vital substrate for membrane synthesis and energy storage [364]. Overexpression of FASN suppresses TNF-α signaling and impairs immune surveillance, promoting tumor progression [365]. Elevated levels of phosphocholine and glycerophosphocholine, intermediates in phospholipid metabolism, have been detected by magnetic resonance spectroscopy (MRS) in BC and PCa serve as diagnostic metabolic signatures [366].

Dysregulated adipokine signaling also contributes to cancer risk. Hypoadiponectinemia is associated with insulin resistance, atherosclerosis, and colorectal tumor development [367]. Mutoh et al. demonstrated that hypoadiponectinemia promotes intestinal polyp formation and CRC, linking metabolic and inflammatory pathways in cancer development [368]. Metabolomic biomarkers are summarized in Table 4.

**Table 4:** Summary of inflammatory and metabolomic biomarkers in aging and cancer.

Biomarker Class	Representative Molecules	Sample Type	Detection Platform	Evidence Level
Inflammatory	IL-6	Blood/Serum/Plasma, Secreted media	Multiplex platforms, ELISA, Western Blot	Preclinical, clinical [96,102,319,322,324]
	IL-1β	Blood/Serum/Plasma, Secreted media	Multiplex platforms, ELISA, Western Blot	Preclinical, clinical [96,99,319]
	TNF-α	Blood/Plasma, Serum	Multiplex platforms, ELISA	Preclinical, clinical [168,319,324]
	NF-κB	senescent cells	Western blot, Luciferase Assay	NF-κB activation has been observed in numerous age-related diseases [96,168]
	CD4+, CD8+	T cell surface markers	RT-PCR, Flow Cytometry	in vivo mouse models, Elevated IL-6 in aged hosts impairs CD8+ T cell function, severely compromising CD4+ T cell-mediated antitumor responses [322]
	IL-10	bone marrow cells, blood from mouse models	RT-PCR, ELISA, Microarray Analysis	age-related ineffective erythropoiesis animal models [324]
	CRP	Blood	ELISA	phase II clinical trial [325]
	CHI3L1(YKL-40)	Serum, CSF, tissues	ELISA	Preclinical, clinical [325,326]
Metabolic	HbA1c	Blood	an immunoassay, HPLC	≥6.5% for diabetes [331]
	AGEs: CML, CEL, Glucosepane, N2-Carboxyethyl-2’-deoxyguanosine	Blood, tissue, urine, cell membranes	Electrophoresis, Spectroscopy, NMR, MS	In vitro, in vivo, clinical [327]
	sRAGE: esRAGE, cRAGE	plasma	ELISA	cohort study [332]
	HK2	Lung and breast from Mouse models, human cancer cell lines	Immunoblot, IHC, PET, LC-MS/MS	In vitro, in vivo [335]
	Lactic acid, LDH	Blood, tumor tissues	Blood lactate test, MRS, MRI	Preclinical, Clinical [340]
	GSH	Blood, RBC, liver, muscle	HPLC, LC-MS	Rodent, human clinical trials [345]
	GLS1	Tumor tissues, patients’ plasma, cell lines	The Cancer Genome Atlas (TCGA) database	Preclinical, bioinformatics analyses [348]
	IDO, TDO	Clinical samples, cell lines, animal models	IHC, HPLC, western blot, MRI	Preclinical, clinical [350]
	IGF-1, IGF-1R	Biopsy tissues, blood	IHC, genetic analysis	Preclinical, clinical [358,359]
	Adiponectin	Blood	ELISA	In vitro, in vivo [360]
	FASN	Tumor tissues, cell line	IHC, gene expression	Preclinical and clinical [364]

Abbreviations: IHC; immunohistochemistry, CSF; Cerebrospinal Fluid, HPLC; High Performance Liquid Chromatography, MS; Mass spectrometry, LC-MS; Liquid Chromatography-Mass Spectrometry.

### 4.5. Protein Biomarkers

Protein biomarkers are fundamental to the diagnosis, prognosis, and treatment of cancer, as well as to the evaluation of biological aging. Multiple clinically validated and exploratory markers have been identified across tumor types and aging contexts, reflecting the complex molecular heterogeneity of disease [32].

Prostate-specific antigen (PSA) is the predominant biomarker for PCa, facilitating early detection and monitoring. Although highly SN, PSA lacks SP because benign prostatic hyperplasia, prostatitis, and malignancy can all elevate serum levels within the “gray zone” (2–10 ng/mL) [369]. To improve diagnostic accuracy, derivative indices such as the free-to-total PSA ratio, glycosylated PSA variants, and the Prostate Health Index (PHI) have been introduced [370]. The PHI, approved by the U.S. FDA in 2012, integrates total PSA, free PSA, and [−2]proPSA into a single composite score and outperforms individual measurements for clinically significant PCa [371].

**Figure 5:**
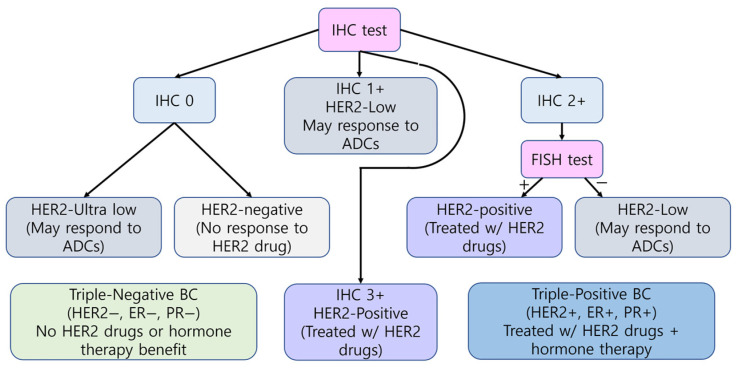
Breast Cancer Diagnosis and Treatment Guidelines by HER2 status. The workflow was laid out by referring to “Breast Cancer HER2 status” in the American Cancer Society web site [373] and reference paper [372]. IHC results are graded from 0 to 3+, where 0 indicates an ultralow or negative expression (‘−’) and higher scores represent increasing HER2 protein expression. FISH testing further classifies equivocal (2+) cases. HER2-positive (‘+’) tumors are eligible for HER2-targeted therapy (violet box), HER2-low cases may respond to ADCs (gray box), and HER2-negative tumors show no response (light gray box). Combined HER2, ER, and PR status defines triple-positive (blue box) or triple-negative (light green box) BC treatment strategies.

In BC, testing for human epidermal growth factor receptor 2 (HER2), encoded by *ERBB2,* is essential for selecting patients eligible for HER2-targeted therapies such as trastuzumab [372]. IHC remains the principal diagnostic technique, with 3+ scores or 2+ scores accompanied by ERBB2-positive fluorescence in situ hybridization (FISH) results defining HER2-positive BC [372,373]. Recently, the classification of HER2-low tumors (IHC 1+ or 2+/FISH-negative) has gained clinical significance due to the responsiveness to antibody-drug conjugates (ADCs) (e.g., trastuzumab deruxtecan). Alongside HER2, estrogen receptor (ER) and progesterone receptor (PR) are utilized in diagnostic procedures. Triple-negative BC is characterized by the low levels of HER2 and the lack of ER and PR. These are negative for HER2, ER, and PR. Hormone therapy and HER2-targeted drug therapy are not effective in these tumors. Triple-positive BC is characterized by the presence of HER2, ER, and PR. This cancer type is managed through hormone therapy and HER2-targeted therapy. The HER2 status and diagnostic and prescribing flow for BC are summarized in Figure 5.

HER2 testing methods approved by the FDA, along with IHC methods, are summarized in Table 5. The HercepTest provide standardized HER2 assessment; however, their limited dynamic range and subjectivity highlight the need for improved quantitative methods, including FISH, chromogenic in situ hybridization (CISH), RT-PCR, quantitative immunofluorescence (QIF), and RNA-based assays [372]. However, resistance poses a significant hurdle [32].

Carcinoma Antigen 15-3 (CA 15-3) serves as a tumor marker for various cancer types, particularly BC [374]. Elevated CA15-3, alongside alkaline phosphatase (ALP), was associated with a heightened likelihood of early recurrence in BC [375].

Cancer antigen 125 (CA-125) is an antigenic tumor marker of ovarian cancer (OC), used to track its development and recurrence using blood test [376]. However, its limited SN restricts its application in the early detection of OC [376]. To compensate for the low SN and SP of CA-125 alone, it is utilized alongside additional protein markers. The combinations of CA-125, human epididymis protein 4 (HE4), and soluble EGFR (SN 93.33% and SP 85.11% for alone vs. SN 83.3% and SP 100% for combination) [377], as well as CA-125, HE4, E-cadherin, and IL-6 (SN 90.4% and SP 87% for alone vs. SN 86.4% and SP 100% for combination) [378], demonstrated reduced SN and increased SP, meeting the ideal SN/SP criteria (SN 75% and SP 99.6%) [379]. Due to the low SN and SP of CA-125 for OC diagnosis, CA-125 alone is not used as a standalone test. Instead, some multivariate test methods including CA-125 have been cleared by the FDA for assessing the risk of malignant tumors in women with adnexal masses who are already scheduled for surgery (Table 6).

In the context of HCC, alpha-fetoprotein (AFP), des-gamma-carboxy prothrombin (DCP), and the glycosylated isoform AFP-L3 are clinically relevant [32]. ALP-L3 exhibits high SP for HCC but low SN when used alone. Of the three markers, AFP-L3 is the sole one cleared by the FDA for risk stratification [380]. Combining AFP, AFP-L3, and DCP significantly improves accuracy, especially in AFP-negative or small HCC lesion [380].

Carcinoembryonic antigen (CEA) and cancer antigen 19-9 (CA19-9) widely applied in the postoperative monitoring and prognosis of gastrointestinal (GI) malignancies [32]. In CRC patients, both elevated preoperative CEA and CA19-9 predicted shorter recurrence-free survival, while in PC both markers correlated with overall survival [381,382]. Combined CEA/CA19-9 measurement improved recurrence risk assessment following curative surgery.

**Table 6:** FDA-Cleared CA-125-Based Tests [382].

Test Name	Type	Components	FDA Clearance Year	Notes/Indications	SN	SP
ROMA (Risk of Ovarian Malignancy Algorithm)	Algorithm	CA125 + HE4 + menopausal status	2011	Preoperative risk stratification for epithelial ovarian cancer (EOC) in women with adnexal masses	~94–95%	~76–80%
OVA1	Multivariate index assay	CA125-II + Transthyretin (TTR) + Apolipoprotein A-1 (ApoA-1) + Transferrin (TF) + β2-microglobulin	2009	Pre-surgical assessment of adnexal mass malignancy risk.	96% (postmenopausal)/85% (premenopausal)	28–40%
Overa (OVA2)	Second-generation OVA1	CA125-II + HE4 + ApoA-1 + TF + Follicle-stimulating hormone (FSH)	2016	Improved version of OVA1 for adnexal mass risk assessment	91%	69%

In small cell lung cancer (SCLC) patients, serum pro-gastrin-releasing peptide (ProGRP), neuron-specific enolase (NSE), and cytokeratin 19 fragment (CYFRA 21-1) levels closely correlated with treatment response and overall survival [383]. These markers effectively predicted prognosis, monitored disease progression, and often detected relapse months before radiological evidence, demonstrating strong value for continuous management of SCLC. Combined measurement of CEA, CA19-9, and CA72-4 improved the diagnosis and follow-up of advanced gastric cancer, particularly when CEA was undetectable [384]. While not effective for early screening, these markers served as prognostic markers, aiding in recurrence detection and treatment decisions.

In cervical cancer, serum proteins CEA, squamous cell carcinoma antigen (SCCA), high mobility group box chromosomal protein 1 (HMGB1), and CYFRA 21-1 were analyzed in 36 cervical cancer patients to identify reliable biomarkers associated with human papillomavirus (HPV) infection [385]. The results showed that CEA had the highest detection rate and exhibited the strongest association with HPV-16.

Immunotherapy-related markers such as programmed death-ligand 1 (PD-L1) have transformed treatment paradigms in lung, melanoma, and renal cancers by guiding checkpoint inhibitor therapy [386].

Beyond oncology, protein biomarkers of aging provide quantitative metrics for assessing biological age and predicting the onset of chronic and neurodegenerative diseases [387].

Blood-based proteins, including p-tau181, p-tau217, neurofilament light (NfL), and glial fibrillary acidic protein (GFAP), are strongly associated with AD and all-cause dementia, showing high predictive accuracy (AUC ≈ 0.71–0.83) and improved performance when combined, especially p-tau217 with NfL or GFAP [388]. Proteomic studies from the Framingham Heart Study identified circulating proteins such as GDF15, NT-proBNP, CRP, leptin, IGF-1, and sRAGE, which correlate with cardiovascular events, heart failure, and mortality, reflecting their involvement in metabolic and inflammatory pathways [389]. Representative protein biomarkers for cancer and aging diagnosis are summarized in Table 7.

Comprehensive proteomic aging clocks, such as ProtAge, have extended this concept by integrating hundreds of proteins to predict biological age and multi-morbidity risk. The simplified ProtAge20 model maintains ~95% accuracy of the full model using only 20 proteins, representing pathways of extracellular-matrix (ELN), immune (GDF15, CXCL12), hormonal (FSHB, AGRP), and neural (GFAP, NEFL) function [387].

The ProtAgeGap, defined as the difference between predicted and chronological age, correlates with the risk of Alzheimer’s, chronic kidney disease (CKD), and all-cause mortality, demonstrating the clinical utility of proteomic age as a surrogate for biological health [387]. For each one-year increase in ProtAgeGap, the risk of developing AD rose by 1.16 times, all-cause dementia by 1.12 times, and CKD by 1.10 times. Individuals in the top 5% of ProtAgeGap had significantly greater odds of AD (2.6 times), CKD (1.8 times), and total mortality (1.9 times) [387].

Proteins such as ELN and GDF15 demonstrated the potential as therapeutic targets, providing the foundation for precision medicine to enable early intervention and promote healthy aging [387]. The selected 20 proteins are summarized in Table 8.

## 5. Early Detection Strategies for Cancer

Cancer ranks among the primary causes of mortality globally. Early detection of cancer in its initial stages or precancerous changes facilitates timely intervention, thereby slowing or preventing disease progression and mortality [27]. Late diagnosis significantly contributes to mortality rates in low- and middle-income countries. Equitable access to early diagnosis strategies is crucial to address this gap [27]. Recent literatures on the advancement of early cancer screening and diagnostic methods demonstrates significant progress through the integration of liquid biopsy technology, multi-omics biomarker analysis, and AI. These innovations have contributed to the development of multi-cancer early detection (MCED) tests, which exhibit high SN and SP in simultaneously identifying multiple cancer types. SN is defined as the proportion of correctly classified true positives among all samples assigned to the positive class, including true positive and false negative. In contrast, SP is defined as the proportion of correctly classified true negatives among all samples assigned to the negative class, including true negative and false positive [390]. Table 9 summarizes common evaluation metrics used in cancer diagnostics to aid understanding before discussing cancer diagnosis strategies.

### 5.1. Liquid Biopsy Approaches

Liquid biopsies provide a novel and minimally invasive method for cancer diagnosis and monitoring. The analysis focuses on cancer-related markers present in various bodily fluids, including blood, urine, and cerebrospinal fluid [394]. This method presents a systematic and thorough alternative to conventional invasive tissue biopsies, offering an evolving perspective on disease progression over time [395]. Currently, liquid biopsies include various components, including circulating tumor cells (CTCs), circulating tumor DNA (ctDNA), EVs, miRNA, circulating RNA (cfRNA), tumor platelets, and tumor endothelial cells [394]. Despite promising developments, challenges remain in terms of SN, SP, and standardization of liquid biopsy technology, particularly in early-stage cancers where tumor-derived biomarkers are scarce [396,397,398]. Biomarkers found in liquid biopsies offer additional insights. ctDNA indicates genetic mutations present in tumors, CTCs signify viable tumor cells capable of metastasis, and exosomes transport molecular cargo that reflects tumor status [399,400]. Liquid biopsy detects various biomarkers associated with cancer in blood and other body fluids and, when combined with various advanced technologies, is expected to be a promising technology for early cancer diagnosis [401,402]. Liquid biopsy biomarkers are summarized in Table 10.

#### 5.1.1. ctDNA

ctDNA is a piece of DNA released from tumors and present in the blood, which is used for detecting mutations, monitoring drug resistance, predicting recurrence, and other purposes [401]. Residual impurities can significantly affect DNA purity during ctDNA extraction. Cell-free DNA (cfDNA) is fragmented DNA found in biological fluids released from cells into the circulatory system [403]. It is present in both healthy individuals and patients with diseases, with ctDNA accounting for 1–2% of total cfDNA [404]. ctDNA can be differentiated from normal cfDNA fragments by the presence of epigenetic or genetic modifications, such as tumor-specific methylation markers and somatic point mutations [401,405]. Because the origin of ctDNA in the bloodstream is derived from CTC, exosomes secreted by tumor cells, apoptotic tumor cells and necrotic tumor cells, ctDNAm can aid in identifying clinical molecular subtypes of cancer and the tissue origin of the tumor [406].

The FDA has approved two blood-based tests (Shield and ColoHealth) for CRC screening in average-risk individuals. Shield test was performed to analyze cfDNA genomic variants, abnormal methylation status, and fragment patterns. This test demonstrated a SN of 83.1% for CRC and a SP of 89.6% for advanced neoplasia in an average-risk screening population [407]. These metrics met the prespecified acceptance criteria for FDA-approved screening tests for CRC. While its SN for advanced precancerous lesions was lower (13.2%), the test’s high SN for actual CRC, particularly for stage I, II, or III (87.5%), makes it a valuable tool for identifying existing cancers [407]. ColoHealth (Epi proColon) test detects methylated *Septin 9* (m*SEPT9*) DNA as a screening tool for CRC [408]. m*SEPT9* has shown higher SN in diagnosing CRC compared to conventional markers like CEA, CA19-9, or Fecal Occult Blood Test (FOBT) [409]. Combining m*SEPT9* with other markers can further increase diagnostic SN, especially for early stages. m*SEPT9*, a representative marker for CRC diagnosis, is stable across disease stages, could potentially be repurposed for detecting other gastrointestinal adenocarcinomas [410].

PC is generally identified at a late stage, characterized by low survival rates and elevated recurrence rates. Therefore, early diagnosis and prognostic or predictive markers are essential for customized therapy [411]. Currently, CA19-9 is the sole FDA-approved biomarker for PC. However, its efficacy is constrained by low SN and SP [411]. Methylation of *BNC1* and *ADAMTS1* has been shown to detect PC at an earlier stage [412]. While generally less sensitive than CA19-9 for the early diagnosis of PC, the combination of ctDNA with mutant *KRAS* and CA 19-9 markedly increased the diagnostic SN to 91% [411]. The integration of ctDNA with mutant *KRAS* demonstrated an effective marker for assessing PC progression during or following chemoradiotherapy or surgical intervention [413].

Changes in ctDNAm patterns are very important for the diagnosis, staging, prognosis prediction, and recurrence detection of breast cancer (BC) and LC. Typical DNAm patterns found in early BC are hypermethylation of tumor suppressor genes such as *BRCA1*, *ITIH5*, and *RASSF1A*, which induce inactivation of these genes [414]. Other types reduce methylation, contributing to the development and progression of the disease. These specific methylation signatures may aid in distinguishing BC subtypes, which is important for patient classification and personalized treatment approaches. Methylation patterns are also associated with treatment response and play an important role in predicting the outcome of immunotherapy. In BC patients, immune cell-specific hypermethylation patterns have been identified, which may contribute to risk stratification and improved treatment management [415].

Nine hypermethylated genes were identified in early-stage lung tumors, and four of these genes (*BCAT1*, *CDO1*, *TRIM58*, and *ZNF177*) formed a diagnostic signature [416]. DNAm analysis of non-invasive samples from 83 LC patients revealed that *BCAT1* is a candidate gene with high diagnostic efficacy in plasma-derived ctDNA [417]. A genome-wide DNAm profiling identified a panel of DNAm biomarkers (*CLDN1*, *TP63*, *TBX5*, *TCF21*, *ADHFE1* and *HNF1B*) in squamous cell LC [418]. Next-Generation Sequencing (NGS) provided comprehensive genome-wide methylation profiling, allowing for the identification of seven differentially methylated regions corresponding to *HOXB4*, *HOXA7*, *HOXD8*, *ITGA4*, *ZNF808*, *PTGER4*, and *B3GNTL1* genes associated with LC [419]. This panel achieved high diagnostic accuracy in distinguishing LC from benign diseases and healthy controls. *SHOX2* and *RASSF1A* genes have demonstrated high SN and SP in clinical studies by showing distinct DNAm patterns in non-invasive samples like blood and sputum [420].

A new liquid biopsy strategy combining circulating cell-free mitochondrial DNA (mtDNA) and ctDNA has improved cancer detection [406,421]. mtDNA exhibits a significantly higher mutation rate compared to nuclear DNA, attributed to the lack of histone protection, inefficient DNA damage repair mechanisms, and the prevalence of highly reactive oxygen species in the surrounding environment. Somatic mutations in mtDNA have been reported across multiple cancer types [422]. Mitochondria contain hundreds to thousands of copies of mtDNA, with the quantity varying by cell type; it is specifically high in tissues with elevated metabolic activity, such as skeletal muscle and liver. These characteristics enable both qualitative and quantitative detection of cell-free mtDNA in patient blood samples [423]. mtDNA analysis can provide useful insights when cfDNA analysis is not feasible. This suggests that mtDNA analysis may reveal characteristics of cancers that are not detectable by ctDNA, such as the aggressiveness of cancer cells or metabolic changes [421].

#### 5.1.2. CTCs

CTCs are cancer cells circulating in the bloodstream and are an important component of tumor spread and metastasis [401]. Evidence suggests that metastasis, the spread of cancer, is an early event in aggressive cancers but is usually detected late, often occurring even before primary tumors are clinically detectable [424]. The lack of CTCs in routine blood sample volumes is one of the main obstacles to using them for early cancer detection. CTC can be used not only for early detection of cancer but also for prognosis and treatment response monitoring [401]. It is challenging to accurately detect cancer in its early stages due to this low number, which restricts the SN of existing detection techniques [424]. In early-stage breast cancer (stage I-IIIA), the detection of more than one CTC is considered significant [425,426]. Although CTCs have been proven to be clinically useful biomarkers at metastatic BC [427], they are rarely detected in early BC, limiting their use as predictive or therapeutic diagnostic biomarkers or as indicators of minimal residual disease in this population [428]. However, utilizing nanostructured titanium oxide-coated slides, Krol et al. identified CTC clusters in a cohort of 28 BC patients, suggesting their role in early metastatic spread [425]. The presence of CTC clusters in early BC may serve as an additional, substantial risk factor for disease progression. Identifying patients with these clusters could help pinpoint high-risk individuals who might benefit from more aggressive or targeted early interventions.

Conventional methods for CRC diagnosis, such as colonoscopy and biopsy, are invasive and may miss asymptomatic patients. As a result, CTC analysis in CRC patients is gaining attention. Recent studies have detected CTCs using specific markers, including the adenomatous polyposis coli (APC) gene mutation, which is present in 60–70% of CRC patients [429]. Additionally, by utilizing established markers such as CK and vimentin in conjunction with the APC gene mutation, the origin of CTCs can be confirmed, thereby enhancing the accuracy, which indicates the proportion of correctly classified true positives and true negatives among all samples [390], and reliability of diagnosis [429]. The metabolic profiles of CTCs have shown potential for early and differential diagnosis of cancer. Yasmin et al. found that eicosanoids, acyl carnitine metabolites, and sterol lipids were specifically increased in CRC cells, suggesting their potential as diagnostic markers [430]. Quantifying mRNA levels of six CRC-related genes in the blood to detect CTCs has been demonstrated [431]. This study on 50 CRC patients using mRNA levels of six genes (CEA, mesenchymal–epithelial transition factor (c-Met), mucin 1(MUC1), CK19, EGFR, and Epithelial cell adhesion molecule (EpCAM)) showed a diagnostic SN of 87% and accuracy of 85%.

A study performed by Purcell et al. has shown that CTC load, PD-L1 expression, and gene expression profiles are valuable biomarkers to monitor and predict patient outcomes in stage III NSCLC [432]. A more substantial decrease in CTCs during chemoradiation therapy correlated with a significantly extended progression-free survival (PFS). Patients exhibiting a CTC reduction rate of less than 75% from baseline to 4 weeks post-treatment demonstrated a PFS of 7 m, markedly shorter than the 21 m average observed in the control group [432]. In patients receiving durvalumab, those who later experienced disease progression exhibited consistently elevated levels of PD-L1+ CTCs compared to patients with stable disease. Elevated PD-L1+ CTC ratios in patients receiving durvalumab were associated with reduced PFS [432]. CTCs isolated during chemoradiation therapy exhibited elevated gene expression linked to an aggressive and proliferative phenotype, indicating that surviving CTCs may possess enhanced metastatic and aggressive characteristics [432].

#### 5.1.3. EVs and Plasma Proteomic Biomarkers

EVs are enclosed by a lipid bilayer membrane measuring 40–150 nm in size. They originate from the endosomal compartment within cells and are secreted by nearly all eukaryotic cells [433]. EVs are involved in cell-to-cell communication, and EVs derived from tumor cells carry important information related to tumor progression [434]. EVs are found in all body fluids, offering a non-invasive way to study originating cells, their oncogenic transformations, the TME, and immune system homeostasis [434]. EVs transport molecular components such as proteins, nucleic acids (RNA), and lipids from tumor-producing cells, carrying tumor-specific markers and providing tumor molecular information [435].

In PC, particularly in its early stages, a high prevalence of mutant *KRAS* in circulating exosomal DNA (exoDNA) has been observed [436]. This mutation serves as a pivotal signal for malignancy, given *KRAS*’s critical role in oncogenesis. The rate of detection of *KRAS* mutants in exosomes was found to be superior to cfDNA across all stages of PDAC studied [436]. This reflects that exoDNA originates from viable cancer cells rather than dying tissue (cfDNA), making it particularly useful in the early stages of cancer. Moreover, elevated levels of EVs containing a cell surface proteoglycan, glypican-1 (GPC1) have emerged as a potential biomarker, showing markedly higher concentrations in patients with PDAC [437] and CRC [438] than in healthy individuals. In CRC, miR-96-5p and miR-149 have been proposed as factors regulating GPC1 expression and exosome secretion [438]. The integration of EV-derived miRNA (miR-23a-3p, miR-92a-3p, and miR-150-5p with CEA has enabled high-precision early diagnosis of CRC [439].

Urine-derived exosome ncRNA demonstrated PCa classification with high precision [440]. Three platforms were used for analyzing ncRNA. The Sentinel™ PCa Test distinguishes PCa from controls, the Sentinel CS Test stratifies low-risk (Grade Group 1) and intermediate/high-risk (Grade Groups 2–5) patients, and the Sentinel HG Test stratifies low-risk/favorable intermediate-risk (Grade Groups 1–2) and high-risk (Grade Groups 3–5) patients. Validated in 1436 subjects, the PCa Test demonstrated 94% SN and 92% SP, the CS Test showed 93% and 90%, and the HG Test exhibited 94% and 96%, respectively.

Kim et al. identified SCLC-specific exosomal miRNAs through the analysis of serum samples from SCLC patients and healthy controls [441]. A trio of miRNAs (miR-200b-3p, miR-3124-5p, and miR-92b-5p) was identified as notably significant. The combination of these miRNAs enhances the SP of SCLC diagnosis relative to the utilization of individual miRNAs, evidenced by an AUC value of 0.93 for the combined miRNAs compared to 0.64–0.76 for single miRNAs. Furthermore, this study confirmed that a panel of three miRNA was significantly associated with poor prognosis in SCLC, suggesting that this panel may also act as prognostic biomarkers. miR-29a-3p demonstrated potential as a promising prognosis biomarker in early-stage NSCLC, which could play a key role in the treatment and management of patients at increased risk of metastasis [442]. While the paper primarily draws attention to the prognostic value of miR-29a-3p, the study found that the expression levels of miR-29a-3p in plasma EVs from NSCLC patients were significantly different compared to those in healthy donors (*p* = 0.004). This difference suggests that miR-29a-3p could potentially serve as a marker to differentiate between NSCLC patients and healthy individuals.

For NSCLC, detection of EVs-based *EGFR* T790M mutation resistant to EGFR inhibitors was carried out [443,444]. The exosomal nucleic acids (exoNA) from plasma enhanced detection, particularly for low T790M copy numbers, early-stage NSCLC and its early identification through EVs could inform personalized treatment strategies. In NSCLC, exosomal PD-L1 is known to promote tumor growth through immune evasion [445]. A substantial number of patients demonstrate inadequate responses to PD-1/PD-L1 inhibitors, underscoring a pivotal challenge in the treatment of NSCLC [446]. Shimada et al. demonstrated that serum exo PD-L1 levels could serve as a valuable quantitative factor to predict anti-PD-1 response in NSCLC patients [447]. Patients with serum exo PD-L1 levels ≥ 166 pg/mL demonstrated a 100% disease control rate when treated with anti-PD-1 inhibitors, indicating its potential as a strong predictor for favorable responses.

BC patients demonstrate significantly elevated phosphorylated protein levels of select EV proteins—including Ral GTPase-activating protein subunit alpha-2 (RALGAPA2), cGMP-dependent protein kinase1 (PKG1), tight junction protein 2 (TJP2), and nuclear transcription factor, X box-binding protein 1 (NFX1) [448]. OC diagnosis may benefit from a panel of seven EV protein markers: EGFR, HER2, CA125, folate receptor alpha (FRα), cluster of differentiation (CD)24, EpCAM, and the combination of CD9 and CD63 [449]. These markers have proven effective in differentiating early-stage OCs from healthy controls. Another independent study identified FGG, MUC16, and APOA4 as key EV proteins capable of distinguishing malignant ovarian tumors from benign cystadenomas or healthy tissue [450].

Circulating proteins have shown promising results in early-stage cancer detection for LC, CRC, BC, OC, and HCC. Gasparri et al. identified Raf Kinase Inhibitory Protein (RKIP) and its phosphorylated form (pRKIP) as potential biomarkers for early-stage LC. Serum levels of RKIP were found to be significantly higher in early-stage LC patients compared to healthy controls, allowing for discrimination with 93% accuracy (AUC 0.94) [451]. The ratio of RKIP to pRKIP further enhanced diagnostic accuracy, particularly in distinguishing early-stage LC patients from high-risk healthy subjects (AUC 0.79).

AFP and vitamin K absence or antagonist-II (PIVKA-II) in serum were analyzed for diagnosis of HBV-related HCC [452]. Diagnostic performance showed AUCs of 0.765 (AFP), 0.901 (PIVKA-II), and 0.917 (combined), with combined detection superior. Both markers correlated positively with tumor size, differentiation, and vascular invasion. PIVKA-II alone or combined with AFP improves HCC diagnosis. Another study demonstrated that the diagnostic accuracy for HBV-HCC is significantly improved when AFP, PIVKA-II, and golgi glycoprotein 73 (GP73) are used together, particularly in the chronic hepatitis B patient population [453].

While many circulating proteins serve as predictive and prognostic biomarkers in BC, fewer are specifically highlighted for early detection. Serum LRP6N, haptoglobin (Hp), and apolipoprotein C1 (APOC1) have been identified as potential biomarkers for the early detection of BC through blood proteomics [454]. In vivo, LRP6N inhibited SDF-1/CXCR4-driven lung metastasis and prolonged survival, whereas LRP6 knockdown enhanced metastasis [455]. A secreted serum form of LRP6N, reduced during metastasis, was identified in both mice and patients. Thus, LRP6N may serve as a diagnostic marker and therapeutic inhibitor of breast cancer metastasis. APOC1 was demonstrated as a potential diagnostic serum biomarker for breast cancer (BC) through a comprehensive proteomic analysis [456]. Validation confirmed progressively elevated APOC1 expression from healthy controls to fatty breast disease, mastopathy, and BC patients. Thus, APOC1 is a promising serum biomarker for BC diagnosis. Hp is a prevalent human plasma protein that effectively binds hemoglobin during hemolysis [457]. Hp expression was increased in breast cancer tissue and the circulatory system, and it was found to promote tumorigenesis by regulating the cell cycle and apoptosis [458]. The N-glycosylation of disease-specific Hp β (DSHp-β) was evaluated as a biomarker for distinguishing BC from benign breast lesions [459]. Analysis of serum samples from 497 patients demonstrated that the DSHp-β N-glycosylation signature can serve as an excellent serological biomarker for breast cancer diagnosis and may provide mechanistic insights into tumorigenesis.

CancerSEEK, an innovative multi-analyte blood test designed for the early detection and localization of cancers of the ovary, liver, stomach, pancreas, esophagus, colorectum, lung, or breast, has been developed. This test combines genetic and protein biomarkers to achieve high SP and SN, demonstrating particular effectiveness for cancers lacking existing screening tests [460]. Genetic markers (*KRAS*) and eight specific proteins, including CA-125, CEA, CA19-9, PRL, HGF, osteopontin (OPN), myeloperoxidase (MPO), and tissue metalloproteinase inhibitor 1 (TIMP-1), are used. The OncoSeek test uses a panel of seven protein tumor markers (PTMs), including AFP, CA125, CA15-3, CA19-9, CA72-4, CEA, and CYFRA 21-1 [461].

#### 5.1.4. Non-Coding RNA

miRNAs in blood are increasingly recognized as non-invasive biomarkers for the diagnosis of various diseases, especially cancer, and for prognostic prediction [462]. miRNAs are rapidly released into bodily fluids from injured tissues, creating a profile that indicates particular disease conditions. This enables the early detection of diseases and the forecasting of recurrence risk.

miR-21 is a representative oncogenic microRNA that regulates post-transcriptional gene expression and is overexpressed in various cancers, including glioma, BC, and CRC [463]. Its major target genes include PTEN, proteolysis-targeting chimera, programmed cell death protein 4 (PDCD4), reversion-inducing cysteine-rich protein with Kazal motifs (RECK), and signal transducer activator of transcription 3 (STAT3), which are involved in apoptosis, invasion, metastasis, and proliferation. Consequently, miR-21 has been gaining attention as a diagnostic and prognostic biomarker for cancer, as well as a therapeutic target. Research and clinical trials are underway for anti-miR-21 oligonucleotides and a chemically modified miR-21 inhibitor (ADM-21)-based inhibition strategies. However, its potential utility as a tissue-specific biomarker requires careful consideration [463].

In OC, the combination of six miRNAs—miR-200a-3p, miR-766-3p, miR-26a-5p, miR-142-3p, let-7d-5p, and miR-328-3p—demonstrated the highest diagnostic performance, achieving an AUC of 0.994, SN of 98.4%, and SP of 95.6% [464]. This indicates that these miRNAs can distinguish malignant from benign OCs with very high accuracy, outperforming most single conventional markers. Furthermore, the combination of miRNAs with CA125 and HE4 yielded superior diagnostic results compared to using each marker alone. For instance, the combination of CA125, HE4, and exosomal miR-205 achieved 100% SN and 86.1% SP, which markedly exceeds the performance of CA125 or HE4 alone [465]. Similarly, the combination of miR-1307, CA125, and HE4 (AUC 0.970) and the combination of miR-375, CA125, and HE4 (AUC 0.945) demonstrated superior diagnostic accuracy compared to the individual markers: miR-1307 (AUC 0.671), miR-375 (AUC 0.788), CA125 (AUC 0.905), and HE4 (AUC 0.820) [466].

An integrated system combining an efficient EV separation platform and advanced AI analysis was used to analyze plasma samples collected from 80 CRC patients and 20 healthy individuals. The optimal combination of miR-23a-3p, miR-92a-3p, miR-150-5p, and the most commonly used CEA in relation to CRC, achieving an AUC of 0.9861 for overall CRC detection, surpassing individual markers and traditional CEA testing [440]. This biomarker combination demonstrated high SN (95.83%), SP (100%), and accuracy (96.67%) for overall CRC.

### 5.2. Advances in Diagnostic Technology

This session discusses DNAm detection methods in liquid biopsy and various advanced technologies recently developed to enhance the SN and SP of liquid biopsy.

#### 5.2.1. DNAm/Tumor DNA Detection Methods

DNAm, a crucial epigenetic modification, can be detected using various techniques, primarily focusing on specific sites within the genome [412].

Methylation Specific PCR (MSP) technique is used to detect DNAm at specific sites. At first, DNA is treated with sodium bisulfite to convert unmethylated cytosines to uracil while methylated cytosines remain unchanged [467]. Subsequent PCR amplification with methylation-specific primers allows for the detection of methylated sequences [467]. This method enables early cancer detection with high SN, even at 0.1% methylation [468]. It is cost-effective, rapid, and compatible with liquid biopsy samples using minimal DNA, though it may yield false positives from incomplete bisulfite conversion [468].

Whole Genome Bisulfite Sequencing (WGBS) is a comprehensive technology that determines methylation status across all CpG sites in the entire genome. This approach allows for a thorough understanding of differential methylation patterns between cancer patients and healthy individuals [412]. WGBS offers extensive, single-base methylation analysis following bisulfite conversion and NGS, even with minimal DNA quantities [469]. It facilitated early BC detection with high precision (AUC ~ 0.97) [469], aided in the diagnosis of esophageal cancer through 5-hydroxymethylcytosine (5hmC) multimodal analysis [470,471], and provided methylation landscapes across various cancers. Despite its efficacy, the high cost frequently limits research to small cohorts, necessitating subsequent validation at specific loci within larger populations [412]. Specialized cfDNA library preparation is necessary; however, its accuracy renders WGBS an essential instrument in cancer epigenomics [472].

Digital PCR (dPCR) offers a highly sensitive and quantitative way to detect and measure DNAm, particularly useful for low-abundance DNA samples [412]. ddPCR is a reliable method for detecting nucleic acid markers from cf DNA/RNA, CTC, and exosomes in blood, plasma, or serum. It helps predict tumor relapse and reveals intratumor heterogeneity and clonal evolution, offering valuable insights into treatment response across various malignancies [473]. Several studies have demonstrated the utility of ddPCR for analyzing the genetic profile of LC. In sputum cytology-positive cases, ddPCR showed high SN for EGFR mutations in LC [474], and combining sputum and plasma improved the detection rate of EGFR T790M during disease progression in NSCLC [444,475]. Furthermore, ddPCR using bronchoalveolar lavage fluid (BWF) achieved higher SN and diagnostic accuracy than plasma, particularly in early-stage disease in LC, indicating that BWF is a superior alternative for detecting mEGFR mutations [476].

NGS rapidly, accurately, and cost-effectively analyzes large volumes of genetic information, aiding in identifying disease causes and developing personalized treatments [477]. In the first-generation sequencing methods, Sanger’s chain-termination method uses dideoxy nucleotides to generate fragments of varying lengths, while Maxam–Gilbert sequencing employs chemical cleavage of nucleotides and works best for short DNA fragments. These foundational methods enabled later technological advances [477,478]. To overcome their low throughput, the second-generation sequencing (or NGS) emerged around 2005. NGS involves DNA fragmentation, single-stranded library preparation, and clonal amplification on beads or slides, followed by massively parallel sequencing [477]. Major platforms include 454 Roche (Roche), which uses pyrosequencing and bioluminescent real-time detection, and Illumina Genome Analyzer from Illumina, which uses sequencing by synthesis and evolved into the HiSeq 2000 and MiSeq [479]. The ABI SOLiD system (Applied Biosystems) applies sequencing by ligation, offering high throughput but short reads, while the Ion Personal Genome Machine (PGM) from Ion Torrent detects pH changes for fast, cost-effective sequencing [479]. Later, the third-generation sequencing (TGS) addressed NGS limitations by producing long reads without PCR, enabling real-time single-molecule sequencing. Platforms like PacBio and MinION (Oxford Nanopore) generate kilobase-scale reads more quickly and cheaply, though with higher error rates that can be reduced by increasing coverage [477,480].

NGS is applied to WGS, targeted sequencing, ChIP-seq, and RNA-seq [477]. WGS provides complete genetic information for disease research and drug response prediction [480]. Targeted sequencing enables faster, more cost-effective analysis of specific genes with higher coverage. ChIP-seq maps DNA-protein interactions to elucidate gene regulation and epigenetic mechanisms, thereby advancing precision medicine and improving diagnostic accuracy [481]. RNA-seq provides information solely on transcribed sequences, including both coding and non-coding RNA, enabling precise identification of functionally relevant alterations, increasing diagnostic rate by up to 10–35% [482].

Both dPCR and NGS platform technologies are the two primary high-resolution nucleic acid quantification methodologies in biomarker-based diagnostics. However, owing to their foundational operational concepts, such as the partition-based quantification of digital PCR and the extensively parallel sequencing of next-generation sequencing, they exhibit unique analytical advantages and constraints. To quantitatively compare the two approaches, Table 11 summarizes representative performance parameters reported in the literature.

#### 5.2.2. RNA-seq in Liquid Biopsy

RNA-seq provides deep analysis of the transcriptome, enabling the detection of novel RNA transcript variants, gene fusions, transcript isoforms, splice variants, and chimeric gene fusions involving previously unidentified genes or transcripts [498]. Specifically, RNA-seq can overcome technical limitations that DNA-based methods may have in detecting gene fusions (e.g., difficulty in detection due to long introns or repetitive sequences, lack of direct evidence for fusion expression at the mRNA level) [499]. RNA-seq can detect fusion events even in samples with low tumor cell content or unsuitable for DNA sequencing, thanks to the high expression of fusion mRNAs [499]. Furthermore, RNA-seq can determine whether mutations are expressed and their expression levels, filtering out cases where DNA mutations may be merely potential and clinically irrelevant, and directly assessing the functional impact of gene mutations [500].

RNA-seq is expanding its potential through the analysis of extracellular RNA (exRNA) in liquid biopsies [498]. ExRNA stably exists outside cells and is more readily accessible than tissue, enabling more frequent longitudinal sampling [498]. It offers significantly higher copy numbers than cell-free DNA, potentially providing greater SN, and differences in expression levels can serve as indicators of damage or disease states in specific organs or tissues [498]. The potential of exRNA has led companies like Exosome Diagnostics to develop ExoDx Lung (ALK), an exRNA-based clinical diagnostic test that measures the EML4-ALK transcript from exosomes in the plasma of NSCLC patients [498].

DNA sequencing and RNA-seq possess complementary characteristics, and when combined for analysis, they provide diagnostic information difficult to obtain from a single platform [499]. For example, in pediatric cancer patients, integrating WGS and RNA-seq identified one or more mutations in 93.7% of patients and therapeutic targets in 71.4%. This integrated approach also contributed to diagnostic changes [501].

Each of the RNA-seq pipeline’s components—sequence mapping algorithms, expression quantification algorithms, expression normalization methods, RNA extraction and quality control, data filtering and variant detection, splicing analysis algorithms, and bioinformatics pipeline integration—directly contributes to gene expression estimation quality (accuracy, precision, and reliability). To maximize the clinical utility of RNA-seq data, the pipeline must be carefully designed, optimized, and validated on a continuous basis [498,499,501,502,503].

#### 5.2.3. Microfluidic Devices (MDs)

Microfluidics integrates mixers, actuators, reactors, separators, and sensors onto a chip to optimize detection processes [504,505]. Microfluidic devices’ sophisticated systems and miniaturization reduce sample consumption, reaction times, detection SN, and costs [506]. Microfluidic biosensors excel in the isolation, enrichment, and detection of rare cells within complex biological samples. The efficiency of CTC enrichment in liquid biopsies is significantly influenced by MD geometries and surface modifications. Various strategies have been developed to enhance CTC isolation, focusing on both physical properties and biological characteristics of the cells [507]. Combining physical and magnetophoretic separations has achieved high separation efficiencies, with reported rates of 100% for CTCs and 93.3% [508]. Using red blood cell membrane mimics for surface coating to prevent leukocyte contamination and achieve pure CTC separation, a surface was developed that excludes blood cells while capturing tumor cells, achieving a capture efficiency of 91% and a purity of 89% [509]. Furthermore, the incorporation of tumor-specific ligands, such as folate and RGD peptides, onto the surface improves CTC binding, markedly augmenting the enrichment factor [509].

Mishra et al. developed a novel high-throughput MD specifically designed for the enrichment of CTCs from large blood volumes, such as entire leukapheresis products [510]. Traditional liquid biopsies using standard blood draws (e.g., 10 mL) are limited by the extreme rarity of CTCs, which typically yield 0 to 10 CTCs per patient. This new technology can process complete leukapheresis products (averaging 5.83 L of blood equivalent), resulting in an average of 10,057 CTCs per patient. This significantly increased yield eliminates the statistical limitations of previous methods, allowing for more robust and reliable analyses [510].

The existing MD method frequently produces heterogeneous CTC clusters, necessitating further processing for thorough analysis and phenotype identification. Hyperuniform micropost MD has demonstrated potential efficacy in categorizing diverse CTC phenotypes via cell trajectory analysis and ML [511]. This device showed high accuracy in classifying two CTC phenotypes, including PC3 (PCa) and SKBR3 (BC) cells [511].

The μTASWako i30, a fully automated immunoanalyzer, was designed for in vitro diagnostics utilizing microfluidic technology [512]. Capillary electrophoresis on a microfluidic chip has facilitated the identification of various glycoform types of AFP, a serum biomarker for HCC. The glycoform AFP-L3 was allegedly more specific for HCC. This assay system offers high SN and delivers rapid results in 9 min.

#### 5.2.4. Surface-Enhanced Raman Scattering (SERS) Biosensors

The integration of liquid biopsy with Surface-Enhanced Raman Scattering (SERS) biosensors significantly enhances the accuracy and SN of early cancer diagnosis [513]. SERS biosensors utilize the enhancement of Raman scattering signals by metallic nanostructures, typically gold or silver, to detect cancer biomarkers at very low concentrations [514,515,516,517]. This sensor, combined with nanotechnology, demonstrates improved detection capabilities. Gold nanorods and other nanoparticles (NPs) are employed to create hotspots that increase signal intensity, enabling the detection of low-abundance biomarker.

Circulating enzyme-mediated DNA amplification technology and gold nanoparticle@silicon-assisted SERS technology have enabled the reproducible identification of single-nucleotide variant ctDNA sequences in diffuse intrinsic pontine gliomas (DIPGs), with a low LoD as low as 9.1 fM in added blood samples [518]. Zhang et al. introduced an ‘immune-like sandwich multiple hotspots SERS biosensor’ designed for highly sensitive and stable CRC marker nucleoside diphosphate kinase A (NDKA) analysis in serum [519]. The practical utility of the biosensor was confirmed by detecting NDKA in serum, demonstrating a LoD of 0.25 pg/mL. Furthermore, it could detect NDKA at 10 ng/mL in mixed protein solutions, encompassing its high SN and SP.

Meanwhile, SERS biosensors face challenges in surface functionalization and signal fluctuation. To address these issues, Nguyen et al. used aluminum (Al) NPs to naturally immobilize DNA without surface functionalization and InGaN quantum wells to stabilize the SERS signals via abundant surface charges [520,521]. The combination of Al NPs for DNA immobilization and InGaN quantum wells for signal stabilization collectively enhances the SN of the biosensor to the single-molecule level.

A “sandwich” immunocomplex assembly strategy, entailing the pre-mixing of EpCAM-functionalized SERS nanotags with sEVs isolated from an OC cell line and the utilization of anti-CD9-conjugated magnetic particles as capture probes, exhibited optimal performance for sEV detection [522]. The enhanced SERS analytical approach was successfully implemented to profile surface protein biomarkers (EpCAM, CA125, and CD24) on the surface of sEVs derived from OC.

Hybrid SERS platforms have been developed to optimize the detection of cancer biomarkers in complex biological samples. Lu et al. developed a versatile microfluidic-SERS barcoding system by utilizing a single-layer, vertically oriented nanorod array, which generates a plasmonic coupling-based electromagnetic field [523]. The method is capable of achieving ideal sensitivities at subfemtomolar levels for four different miRNAs. The system also enables precise classification of tumor stages, metastatic conditions, and subtypes, achieving an overall accuracy of 94%.

Structurally unique molecules exhibit unique Raman scattering spectra. Label-free SERS analysis utilizes the unique Raman spectra of target substances to directly capture these unique spectral fingerprints from the sample itself, thereby identifying disease-related biomarkers [524]. The study performed by He et al. demonstrated a hybrid diagnostic approach that combines multiplexed Raman-tagged antibodies with label-free SERS and the Support Vector Machine (SVM) model, which significantly improved the accuracy, SN, and SP of OC biomarkers detection on EVs to over 95% [524].

#### 5.2.5. Surface Plasmon Resonance (SPR) Biosensors

SPR biosensors have emerged as a powerful tool for cancer detection due to their ability to provide optical, label-free detection of cancer biomarkers with high SN and SP [525,526]. These biosensors leverage the interaction between light and metal surfaces to detect changes in the refractive index, which can indicate the presence of cancerous cells or biomarkers [527]. Recent advancements in SPR technology have focused on enhancing SN, selectivity, and the ability to detect a wide range of cancer types, making them a promising option for early cancer diagnosis and monitoring [526]. The incorporation of materials such as TiO_2_, graphene, and antimonene has significantly improved the performance of SPR biosensors. These materials enhance the SN and binding efficiency of the sensors, allowing for more accurate detection of cancerous cells [525,526,528]. Nanostructures, such as NPs, nanoholes, and nanopillars, facilitate SPR, resulting in localized electromagnetic “hot spots” due to the resonant vibration of free electrons in metallic nanostructures [529]. These hotspots enhance interactions with analytes, thereby improving SN and signal detection [530,531]. SPR biosensor is promising for detecting EV cancer biomarkers with low sample volume and high SN [532]. For instance, a fiber-optic SPR biosensor was developed to detect BC-specific EVs with an LoD of 2.1 × 10^7^ particles/mL in buffer and 7 × 10^8^ particles/mL in blood plasma, demonstrating high SN and SP [533].

#### 5.2.6. Electrochemical Biosensors

Electrochemical biosensors for cancer marker detection are sophisticated devices that integrate various components to achieve high SN and SP. These biosensors primarily consist of a biorecognition element, a transducer, and signal amplification materials, which work together to detect tumor biomarkers effectively [534]. Common biorecognition molecules include antibodies, aptamers, and nucleic acids, which specifically bind to tumor biomarkers such as PSA, HER2, and CA19-9 [534,535]. These elements facilitate the selective recognition of cancer markers, crucial for accurate diagnosis. Electrochemical transducers convert the biological interaction into measurable electrochemical signals. Common types include glassy carbon electrodes modified with nanocomposites like reduced graphene oxide and metal oxides [536,537]. The transducer’s role is vital as it translates the binding event into an electrical signal, which can be quantified. Incorporation of nanomaterials such as gold NPs and metal oxides enhances the SN of the biosensor by increasing the surface area and improving electron transfer [537,538]. Signal amplification allows for the detection of low concentrations of biomarkers, with some biosensors achieving limits of detection as low as 1 cell/mL [539]. Electrochemical biosensors are capable of multiplexed detection, allowing simultaneous measurement of multiple cancer biomarkers. This capability is crucial for providing comprehensive diagnostic information and improving the accuracy of cancer diagnosis [540]. In addition, electrochemical biosensors can improve the vulnerability of false positive or false negative diagnoses caused by heterogeneity within and outside tumors (variations in genetic and protein expression profiles, epigenetic modifications, etc.) [540].

### 5.3. AI and Machine Learning

AI is the study of training machines to mimic human thinking, focusing on learning, reasoning, and self-correction [541]. Machine Learning (ML), a subset of AI, enables systems to automatically learn and improve from experience without explicit programming by analyzing data, recognizing patterns, and making better decisions [541]. Deep Learning (DL), a specialized branch of ML, uses neural networks inspired by the human brain to process information and classify patterns [541]. Unlike ML, DL handles larger datasets and performs self-directed predictions, making it highly effective in complex tasks such as image recognition, speech processing, and natural language understanding [541].

Mammography, CT, MRI, ultrasound, PET, SPECT, and X-ray are all essential imaging modalities for detecting cancer [542]. All are essential for identifying issues. Nonetheless, the interpretation of intricate images relies on the radiologist’s expertise and subjective judgment, potentially resulting in errors and discrepancies [542]. The risk of misdiagnosis and oversight of lesions is particularly elevated when lesions are subtle or when a radiologist is fatigued. Tissue-based biopsy remains the gold standard for definitive diagnosis but it presents certain challenges that render it invasive [394]. Consequently, AI is increasingly recognized as an essential complementary tool that enhances the precision and efficiency of diagnoses through objective and consistent analysis [543].

The integration of ML and DL in cancer early detection is revolutionizing the field of oncology by enhancing diagnostic accuracy, personalizing treatment strategies, and improving patient outcomes [29,544]. These technologies are particularly effective in analyzing complex datasets, such as medical images and genomic data, to identify early signs of cancer that might be missed by traditional methods [29,545,546].

#### 5.3.1. Key Concepts of DL and Diagnostic Features

Unlike traditional ML methods that rely on manual feature engineering, DL excels at automatically learning complex patterns and extracting features from unstructured data such as medical images, text, and genomic data [547,548]. Traditional neural networks generally possess one or two hidden layers. Conversely, DL models utilize deep neural networks (DNNs) comprising multiple hidden layers to learn complex representations and hierarchical structures of data. This facilitates the automated extraction of features from raw data, including the identification of elementary features such as edges in images in the initial layers and the recognition of objects in the subsequent layers [549]. DL models can efficiently process vast amounts of high-dimensional data, which is particularly useful in cancer research that requires complex and heterogeneous biological data [548]. Various DL technologies are being used for early cancer diagnosis, each specializing in different data types and diagnostic objectives.

#### 5.3.2. Convolutional Neural Networks (CNNs)

CNNs represent a category of DL models designed specifically for image data, such as CT, MRI, and histopathology, and are proficient in extracting spatial hierarchical structures of features. They achieve this by automatically learning spatial hierarchies and intricate patterns from raw image data, thereby bypassing the need for laborious manual feature engineering [550]. This indicates that they are capable of recognizing patterns irrespective of their precise location within an image [548]. Public datasets like the Curated Breast Imaging Subset of Digital Database for Screening Mammography (CBIS-DDSM) and The TCGA are commonly used for training CNN models, providing a diverse range of images for robust model development [551]. CNNs have revolutionized the detection of early-stage malignant tumors and tumor image classification [549].

CNN demonstrates elevated SN and SP in BC screening through the identification of microcalcifications and subtle architectural distortions. This significantly decreases false positive rates and increases the accuracy of cancer detection [552,553,554]. A prospective, multicenter cohort study evaluated the diagnostic accuracy of breast radiologists both with and without AI-based computer-aided detection (AI-CAD) for screening mammograms in a real-world, single-read setting [555]. The findings revealed a significant enhancement in the diagnostic accuracy of breast radiologists utilizing AI-CAD. While AI-based software such as the Lunit INSIGHT mammogram AI system + showed slightly lower SN, it outperformed radiologists in recall rate, SP, positive predictive value (PPV), and AUC [556]. In other comparative study, AI outperformed in terms of SP and PPV in low-density breast tissue [557]. However, radiologists showed greater SN than AI in diagnosing BC in dense breast tissue [557].

While CNNs are powerful for medical image analysis, several combinations and hybrid approaches have demonstrated superior performance for cancer detection, often by addressing the limitations of standalone CNNs or enhancing their capabilities. These superior combinations integrate CNNs with other DL architectures, ML techniques, or advanced preprocessing methods. CNN models have demonstrated high accuracy in LC detection, when combined with other neural network models [558,559,560]. A new combination model diversifies training data and improves model robustness by integrating differential augmentation (DA) into CNN and targeting specific elements like hue, brightness, saturation, and contrast [561]. The CNN+DA model outperformed DenseNet, ResNet, EfficientNetB0, and hybrid models like ensemble models.

The architectural variations in CNNs for PC detection exhibit distinct characteristics compared to those used for other cancers, primarily due to the unique challenges posed by pancreatic imaging. These variations are influenced by the pancreas’s small size, irregular shape, inaccessible location, and the necessity for precise segmentation in CT scans [562,563]. To tackle the complex challenges associated with pancreatic segmentation, including the pancreas’s varied morphology and the noise in medical CT images, a technique for automatic detection and segmentation classification of PC has been devised, employing the ASMO-ELC-Casnet-CNN model [564]. The developed model performs segmentation and classification by applying Spider Monkey Optimization through the fusion of UNET and CNN.

The combination of CNNs and Vision Transformers (ViTs) is a rapidly growing field in medical image analysis, as it effectively merges the strengths of both architectures to overcome their individual limitations [565]. CNNs excel at extracting local, fine-grained features due to their inherent inductive bias, while ViTs are adept at capturing global contextual relationships and long-range dependencies across the entire image. This hybrid approach has led to significant advancements in diagnostic accuracy. For instance, UnetTransCNN is a novel parallel architecture that integrates ViT encoder for global feature extraction with a CNN decoder for local detail restoration [566]. This design allows the model to simultaneously process both global and local information, which is crucial for complex anatomical structures. The model was specifically adapted for 3D medical image segmentation with volumetric convolutions and 3D positional encodings. The Dice score measures the extent of overlap between regions identified by the computer and those designated by experts; a higher value denotes a more precise correspondence, whereas a lower value reflects a discrepancy. The 95% Hausdorff distance (HD95) quantifies the spatial separation between boundaries; a diminished value signifies a strong alignment, whereas an elevated value denotes increased discrepancy. When tested on the Beyond the Cranial Vault (BTCV) data set for abdominal organ segmentation and brain tumor segmentation from MRI and CT data (MSD), along with the kidney tumors from CT scans (KiTS19) data set for generalization evaluation, UnetTransCNN outperformed existing models in abdominal organ segmentation on the BTCV dataset with an average Dice score of 85.3%, especially for difficult regions like the gallbladder and adrenal glands [566]. The model also demonstrated superior performance on the MSD (brain tumors) and KiTS19 (kidney tumors) datasets, achieving high Dice scores and low HD95, and notably faster inference times compared to other models [566].

#### 5.3.3. Recurrent Neural Networks (RNNs) and Long Short-Term Memory (LSTM)

RNNs and their variants, including LSTM and Gated Recurrent Units (GRUs), are specifically designed for the analysis of sequential data, such as patient records, and are capable of modeling time-dependent health trajectories [548]. These methods have shown significant accuracy in gene expression analysis and the classification of cancer subtypes [567]. The integration of RNN and LSTM with CNN has shown promising results in improving the accuracy and efficiency of BC detection and classification. The hybrid CNN-LSTM model achieved high accuracy (99% for binary classification and 92.5% for multi-class classification) in identifying benign and malignant BC, as well as their subtypes [568]. Another hybrid CNN-LSTM model achieved further enhanced diagnostic accuracy and reliability for BC detection and classification [569].

The R-LSTM-CNN framework was proposed for detecting and classifying LC from CT images, outperforming traditional CNN and LSTM models by effectively segmenting and extracting features from lung images [570]. Bidirectional Long Short-Term Memory (Bi-LSTM) networks significantly improve LC detection by leveraging their ability to process sequential data and capture long-term dependencies, achieving very high accuracy in detecting LC [571]. This study showed they could identify cancer with nearly perfect accuracy (99.89%) and were excellent at correctly identifying both positive and negative cases.

#### 5.3.4. Others

Rare cancers often lack sufficient annotated data due to their low prevalence and privacy constraints. To address this, researchers are increasingly turning to generative models such as Generative Adversarial Networks (GANs) and Variational Autoencoders (VAEs). These models can synthesize realistic medical data—ranging from histopathological images to gene expression profiles—thereby augmenting limited datasets and enhancing model generalization [572]. Sun et al. proposes Onto-CGAN, a novel generative framework that combines knowledge from disease ontologies with GANs to generate unseen diseases that are not present in the training data [573]. This study demonstrated that Onto-CGAN can generate new diseases with statistical characteristics similar to actual data and significantly improve ML model training. This novel approach addresses rare disease data shortages and has applications in data augmentation, hypothesis generation, and preclinical clinical model validation.

Transfer learning (TL) models are useful for accelerating model development and improving performance with a small amount of medical data by fine-tuning pre-trained models to medical data [574]. This model can also be particularly useful in cases where it is difficult to obtain large datasets, such as rare cancers. The RareNet framework, developed by Shao et al., demonstrated this by utilizing TL on DNAm data to classify rare cancers with high accuracy, outperforming traditional ML models [575]. TL utilizes insights acquired from training models on extensive datasets, like ImageNet, which comprises millions of images across various categories, and applies this knowledge to more specialized tasks with smaller datasets [576]. Consequently, TL offers a solid framework for enhancing the diagnosis of rare diseases in medical imaging.

Self-supervised learning (SSL) is a paradigm in ML that leverages unlabeled data to learn useful representations, which can be applied to various downstream tasks. This approach is particularly beneficial in scenarios where labeled data is scarce or expensive to obtain [577]. Pai et al. developed a foundation model for cancer imaging biomarker discovery by training a convolutional encoder with SSL using a dataset of 11,467 radiographic lesions [578]. A crucial finding is that the foundation model excels in reducing the demand for training samples in downstream applications, which is highly beneficial in medicine where large labeled datasets are often scarce. This allows for effective biomarker discovery even with limited data.

High-performing models alone are insufficient for clinical integration; interpretability is essential. The “black box” nature of DL models has spurred the development of Explainable AI (XAI) techniques, which aim to make model decisions transparent and trustworthy. This transparency is essential for fostering trust and understanding in AI applications across various domains, including healthcare [579]. Tools such as Gradient-weighted Class Activation Mapping (Grad-CAM), Shapley Additive Explanations (SHAP), and Logical Interpretable Model-agnostic Explanations (LIME) provide insights into feature importance and decision pathways, enabling clinicians and researchers to validate AI outputs [580,581]. Recent studies have applied XAI to cancer research with promising results. For example, Dalmolin et al. used SHAP to identify influential genes in cancer classification models, enhancing both interpretability and biological relevance [582]. In drug repurposing for rare diseases, Perdomo-Quinteiro et al. introduced a graph-based XAI framework that generates semantic explanations for predicted drug candidates, facilitating hypothesis generation and experimental validation [583].

#### 5.3.5. AI-Based Early Cancer Diagnosis

AI technology is being used in modern healthcare systems to improve the effectiveness of cancer screening programs and methods. The use of AI in cancer screening can improve its accuracy, strengthen early detection through more sensitive and specific screening processes, optimize medical resource allocation through efficient targeting of high-risk groups, and enable personalized medical approaches tailored to the individual patient [584].

##### Image-Based Diagnosis

AI excel at detecting subtle patterns that may elude human observation, thereby assisting physicians in expediting and enhancing diagnostic accuracy [585]. CNNs have been developed to detect microcalcifications and architectural distortions in mammograms, which are critical indications of BC [551,552,553,554,556,569]. For example, in a cross-modality fusion technique that combined mammography and ultrasound, a specially created 17-layer CNN achieved an accuracy of 96.4% [554]. A newly developed CNN architecture using the Mammographic Image Analysis Society (MIAS) and Digital Database for Screening Mammography (DDSM) datasets for mammographic image analysis demonstrated accuracies of 99.175% and 98.44%, respectively, on mammographic images, clearly distinguishing the boundaries of cancerous regions in abnormal mammographic images [586]. A computational framework that classifies mammography images using ResNet-50 CNN to diagnose BC achieved an excellent classification accuracy of 93%, which is expected to facilitate the early diagnosis and classification of malignant and benign BC [587]. A hybrid CNN-LSTM model achieved high accuracy of 99.17% and 99.90% on two histological data set, demonstrating enhanced classification accuracy and resilience [569]. CNNs have been used to analyze infrared thermographic images for BC detection [588]. By integrating CNNs with Enhanced Particle Swarm Optimization (EPSO) and Generative Adversarial Networks (GANs) for data augmentation, achieving a recognition rate of 98.8%. and demonstrating the potential for early, reliable, and cost-effective BC screening in real-world clinical environments [588].

A novel Back Propagation Boosting Recurrent Wienmed model (BPBRW) with Hybrid Krill Herd African Buffalo Optimization (HKH-ABO) mechanism has been developed for detecting BC in an earlier stage using breast MRI images, achieving improved accuracy of 99.6% with a 0.12% lower error rate [589]. A new granular computing-based DL model was evaluated using ultrasound image datasets and breast tissue pathology image datasets [590]. The proposed model achieved accuracies of 93% and 95% on the two real-world datasets, respectively. A method that applies deep belief networks (DBN) to ROI images for BC diagnosis has been proposed [591]. The proposed DBN model achieved performance rates of 96.32%, 96.68%, 95.93%, and 96.40% in accuracy, SP, SN, and precision, respectively, suggesting DBN is an efficient and robust algorithm for classifying BC and normal tissue in mammograms [591].

The integration of low-dose CT scans with AI-driven analysis facilitates the detection and classification of early-stage LC lesions [558,559,560,561,592]. Google’s DL model looked at 3D CT scans and did better than expert radiologists in some cases, cutting down on false negatives by 5% and false alarms by more than 10%. These kinds of tools can pick up very small problems on scans that might be missed, which could help find LC at Stage I, when they are much easier to treat [592]. The combination of CNN for image analysis with XAI techniques, particularly Grad-CAM demonstrated a high accuracy up to 93.06% of LC detection [558]. The accuracy of cancer detection significantly increases by over 99% when CNNs are combined with a residual network architecture (ResNet), such as ResNet18 or SE-ResNeXt-50, primarily due to their advanced feature extraction capabilities, ability to handle complex patterns, and robust classification mechanisms [559,560]. CNNs applied to biparametric MRI have surpassed conventional techniques in the precise identification of significant PC lesions [593,594].

CAD–enhanced colonoscopies detected approximately 24% more adenomas than standard screening (44% vs. 36%), due to the capture of small polyps that are easily missed [595]. However, there was no significant improvement in the detection of advanced lesions, and the increase in SN was primarily due to the detection of very small polyps. The identification of small polyps necessitates meticulous evaluation of the potential for early adenoma diagnosis, which may progress to cancer, alongside the financial implications of the unwarranted excision of non-cancerous polyps [595].

##### Genomic and Molecular Diagnosis

AI can be used to make sense of the vast and complex data generated by genomic and molecular analyses, leading to the discovery of new biomarkers and more precise risk predictions [596,597]. The MCED testing is a technology that analyzes molecules such as DNA, RNA, and proteins derived from cancer cells in the blood to assess the possibility of cancer [598]. While some tests can estimate the origin of the cancer, additional tests such as imaging tests or tissue biopsies are required for a definitive diagnosis. MCED testing requires ongoing development to enable the early detection of various cancers before symptoms appear. Currently, these tests have not received FDA approval, and some are only available as laboratory-developed tests under the Clinical Laboratory Improvement Amendments (CLIA) regulations [598].

The integration of MCED with AI significantly enhances the accuracy of cancer early detection through various methodologies and technologies [599]. This multifaceted strategy improves predictive efficacy, enabling more reliable identification of tumors and facilitating clinical applications [600]. An AI-based MCED testing analyzes atypical methylation patterns using ctDNA and applies AI algorithms developed based on large datasets of cancer and non-cancer patients to detect more than 50 types of cancer at all stages with high SP and identify the primary tissue [597]. This test precisely detected two-thirds of the twelve lethal cancers lacking routine screening methods. Because advanced cancer has more tumor DNA in the blood, the detection rate increased with later stages of the disease [597]. The integration of large-scale WGS with real-world clinical data provides actionable insights for personalized cancer care, accelerates biomarker discovery, and demonstrates the potential to establish a robust framework for genomic medicine within national healthcare systems [601].

The SYMPLIFY study investigated the cancer detection performance of the GRAIL Galleri MCED blood test in patients referred for urgent testing for the possibility of female-specific conditions, LC, or lower or upper gastrointestinal cancer in the UK healthcare system, or in patients referred to rapid diagnostic centers with nonspecific symptoms [602]. At an advanced stage, it demonstrated effectiveness in detecting cancer signals with high SP (98.4%) and moderate SN (66.3%). The ML model analyzed cfDNAm patterns and showed stability across various patient subgroups. However, the moderate SN highlights the need to improve the test’s SN (negative predictive value) to enhance clinical utility [603].

The PATHFINDER Study performed in oncology and primary care outpatient clinics at seven US health networks, a convenience sample of adults aged 50 years or older without signs or symptoms of cancer consented to MCED testing [604]. This study utilized a ML model integrating cfDNAm patterns. It showed solid performance with a 99.1% SP and a 98.6% negative predictive value (NPV), effectively detecting multiple cancers, including those without routine screening, at early stages [604].

The SPOT-MAS test showed a non-invasive ctDNA-based test for the simultaneous early detection of multiple cancers in asymptomatic adults [605]. It identified the presence of cancer and its tissue of origin (TOO) through DNAm and fragment size analysis. This enables more precise support for subsequent diagnosis and treatment decisions, allowing healthcare professionals to efficiently determine the areas to focus on during testing. In the study, SPOT-MAS demonstrated NPV of 99.92%, indicating a very high likelihood that cancer will not be present for at least 12 months following a negative result [605]. This helps reduce patient anxiety and minimize unnecessary additional testing.

A recent study evaluated a new MCED test analyzing plasma cell-free DNA using genetic and fragmentomics features from whole-genome sequencing. This test was developed to identify cancer signals and tissue origins. [606]. The MCED test achieved an overall SN of 87.4% and SP of 97.8% in the independent validation cohort. Crucially, it maintained high SN for early-stage cancers (stages I and II), with 79.3% and 86.9% SN, respectively, indicating its potential to detect cancers when they are most treatable. The test demonstrated effectiveness in detecting cancers that currently lack established screening protocols, such as PC and OC. For instance, it achieved a SN of 76.9% for PC and 90.5% for OC, which are often diagnosed at advanced stages [606]. In addition, this study demonstrated an accurate prediction of TOO with 83.5% accuracy. The features help medical professionals to accurately identify the primary site of malignant tumors and take appropriate follow-up measures [606].

Homologous recombination deficiency (HRD) causes genomic instability in cancer and is associated with a higher risk of cancer in BRCA1/2 mutation carriers [607]. HRD reveals SN to Poly ADP-ribose polymerase (PARP) inhibitors. Therefore, accurate HRD detection improves mutational profiling, which aids in detecting genomic instability and guiding targeted therapies in cancer patients [608]. ML has been used to create algorithms such as ovaHRDscar and tnbcHRDscar, which enhance HRD detection in OC and BC, respectively [609]. These algorithms used a powerful combination of bioinformatics, ML, and statistical methods to analyze large genomic datasets and identify specific allelic imbalances, improving clinical outcome prediction and patient selection for HR-targeted therapies.

A DL framework, Genomic Status scan (GSscan), employing LSTM for the estimation of HRD status from low-pass whole genome sequencing data, has been developed [610]. The framework exhibited adaptability to low DNA input levels, suggesting potential applications in plasma cell-free DNA and minimal biopsy samples. Additionally, GSscan exhibited superior accuracy in predicting HRD status in breast and ovarian cancer samples, surpassing current methodologies, and HRD-positive patients identified by GSscan demonstrated enhanced clinical outcomes [610].

CancerSig, an evolutionary supervised learning method was proposed to identify cancer stage-specific miRNA signatures for early cancer predictions [611]. CancerSig demonstrated a mean performance across 15 different cancer types with a 10-fold cross-validation accuracy of 84.27% ± 6.31%, SN of 0.81 ± 0.12, SP of 0.80 ± 0.10 [611]. OncoSeek, a new protein analysis-based MCED approach that utilizes a panel of seven protein tumor markers, has been specifically designed to be cost-effective and practical for use in low- and middle-income countries, leveraging advanced AI models [461]. This method reduced false positive rates compared to traditional methods and improved SP to 92.9%.

A novel approach, the ZAHV-AI system, integrating EV isolation technology with AI-based analysis, has been introduced to enhance the early detection and monitoring of CRC [440]. By combining EV-derived miRNA with the conventional marker CEA through AI-based analysis, high-density early diagnosis was achieved across the entire CRC diagnostic spectrum (AUC 0.9861). Particularly, it demonstrated perfect performance with an AUC of 1.0 in detecting Stage 0–I CRC. This compensates for the low early-stage SN of conventional CEA testing, suggesting the potential for a practical clinical tool that significantly enhances the ability to diagnose and monitor CRC at an early stage.

RiboTIE is a state-of-the-art DL-based tool optimized for analyzing ribosome profiling (Ribo-seq) data to decipher RNA translation positions and identify translated open reading frames (ORFs) [503]. RiboTIE applies cutting-edge ML technologies such as transformer networks to process variable-length inputs (the number of reads mapped to the transcriptome) and perform automated feature extraction. It also utilizes pre-trained models optimized across numerous ribosome profiling samples to enhance overall performance [503]. RiboTIE outperformed other ORF detection tools in terms of SN and precision when identifying standard coding sequence (CDS). For example, in pancreatic progenitor cell samples, RiboTIE detected 64.9% more CDS than ORFquant, with 300% more small CDS under 300 bp in length [506]. It also detected the highest number of CDSs with non-canonical ORF (ncORF) (48, all CUG), outperforming other tools. These findings uncover the presence of previously unrecognized translated proteins or peptides. ncORFs may yield new insights into disease biology and facilitate the identification of novel biomarkers or therapeutic targets for cancer diagnosis and treatment [503].

The HCC Early detection Screening (HES) algorithm enhanced AFP surveillance for HCC by incorporating age, Alanine aminotransferase (ALT), platelet count, AFP rate of change, and cirrhosis etiology [612]. Validated in 7432 cirrhosis patients, it detected early-stage HCC within 6 months with 51.2% SN and 90% SP vs. 46.0% SN for AFP alone, identifying 136.46 vs. 118.01 early HCC cases per 1000 imaging analyses and detecting 56% vs. 50% of cases missed by ultrasound, offering modest but meaningful improvement at no added cost [612]. In the study, at a fixed 10% false-positive rate (FPR), HES V2.0 showed higher SN (~47% vs. 38% for AFP and 41% for GALAD), especially for early-stage HCC, which is the hardest to detect. Machine learning, as utilized in HES V2.0, can leverage dynamic trends, nonlinear effects, and multifactorial interactions, resulting in earlier and more precise detection of HCC in high-risk patients compared to logistic regression analyses such as GALAD and ASAP. These models are compared in Table 12.

##### Multi-Modal Data Integration

Electronic health record data are naturally rich in diverse features that span both time and space, including socioeconomic details, molecular data, and various modalities such as tabular data, images, time-series data, and free text. These data components can be structured or unstructured, providing a comprehensive view of patient information that aids in the development of effective AI-driven cancer detection and screening solutions [584].

A study published in Nature Medicine in 2023 utilized DL techniques on millions of patient records from Denmark and the United States to identify high-risk groups for PC, a challenging cancer to detect early, and successfully predicted its onset up to three years in advance [613]. The model, referred to as “CancerRiskNet,” employed electronic health data, including patients’ diagnostic records, test results, medication prescriptions, and family history. The system identified high-risk patients with notable accuracy by analyzing symptom and medical code combinations that mirrored the early progression pathways of PC. This method may facilitate the early identification of pancreatic tumors at a surgically operable stage by offering tailored screening for the designated high-risk patient population [613].

The Artificial intelligence-aided PCa (APCA) score, derived from routine health check-up data, demonstrated potential to significantly reduce the number of unnecessary prostate biopsies in Asian populations [614]. The APCA score predicts high grade PC (HGPC) using 18 features, including some previously unused markers, and achieved an AUC of 0.76 in the multicenter validation cohort. When applied, it could reduce unnecessary biopsies by 20–38%, but may miss 5–10% of HGPC cases [614].

Orpheus, a new multimodal DL tool that combines H&E whole-slide images and text-based pathology reports, accurately identified high-risk cases for BC (Recurrence Score (RS) > 25) in three separate test groups from different medical centers and countries [615]. With an AUC of 0.89, Orpheus found high-risk cases in TAILORx better than the current nomogram, which has an AUC of 0.73. It also accurately predicted the risk of metastasis recurrence in patients with RS ≤ 25, it achieved an AUC of 0.75 compared to 0.49 for the existing RS assay for hormone receptor-positive early BC, thus indicating potential for personalized treatment and monitoring [615].

Recent studies have employed multimodal data on BC to compare the clinical and molecular characteristics of the disease in African and South Asian women with those in European women, underscoring the necessity for customized diagnostic, therapeutic, and screening strategies that consider the distinct clinical and molecular traits linked to varying genetic backgrounds to guarantee equitable advantages for all communities [616].

##### Topological Data Analysis

In Topological Data Analysis (TDA), topological features (TFs) aim to quantify and describe information about the intrinsic “shape” and “connectivity” of data [617]. This focuses on identifying hidden patterns and structures within data that traditional statistical or ML methods may overlook [617]. Key TDA methods, Persistent Homology (PH) and Mapper, are used to extract and utilize TFs [617].

TFs, when combined with AI technologies, particularly ML and DL, have enabled significant advancements in cancer diagnostics. For example, when analyzing lung tumors in chest CT images, TFs can predict true histology more accurately than radiomic features [618]. This contributes to higher diagnostic accuracy by capturing complementary information in distinguishing benign from malignant tumors and adenocarcinoma from squamous cell carcinoma. It has also been used to classify patients with chronic obstructive pulmonary disease (COPD) [617,619] or detect COVID-19 infection [620] via CT images, demonstrating accuracy equal to or better than existing methods.

TDA is used in cancer research to capture important attributes such as a tumor’s shape, texture, and intensity variations [617]. In BC diagnosis, TFs enhanced DL model performance and help identify subgroups with cancer-related gene mutation profiles that were missed by existing models [621]. In OC and BC histopathology image analysis, the TopOC-1 and TopOC-CNN models improved tumor type discrimination accuracy by up to 8.35% (for TopOC-CNN) by integrating TFs into existing DL models [622]. Specifically, they extracted unique “topological fingerprints” by analyzing the evolution of topological patterns across each color channel [622]. For PC, TDA-based systems predicted tumor aggressiveness from histological images, replicated the Gleason grading system, and identified more refined disease subtypes [623].

DL models are often called ‘black box’ models, making their internal workings difficult to understand. TDA enhances model explainability by quantifying structural and geometric patterns in the data, providing insight into why the model makes specific decisions [624]. The Mapper uses the probability values from the ML classifier as a “lens function,” providing a visual representation of how the model classifies data, thereby offering insight into the model’s decision-making process [617]. For example, it visually shows whether certain data categories are easily identifiable or where the model experiences confusion [617].

Biomedical data is often difficult to process with traditional analytical tools due to its scale and complexity [625]. TDA effectively handles high dimensionality and is robust against noise and non-linearity, providing useful insights even in scenarios with data quality issues [626]. PH can compress large, high-dimensional data into compact, low-dimensional representations while preserving important topological information [617]. ML models require very large datasets for training, but in the biomedical field, it can be difficult to secure such large-scale labeled datasets. TDA can be used even with small datasets insufficient for ML model training, making it particularly important in the field of medical image diagnosis [617].

Beyond visual diagnostics, TDA can also be applied to gene regulatory network analysis. PH is utilized to understand the structural characteristics of cancer cell networks. By identifying significant deviations in topological features (e.g., loops, co-clusters) within gene interaction patterns between cancer and normal samples, it reveals higher-order connectivity relationships contributing to disease complexity [627,628].

## 6. Early Detection Strategies for Aging

Early diagnosis of aging and slowing its rate hold greater economic value than treating individual diseases. It has been reported that slowing aging to extend life expectancy by one year generates $38 trillion in value, while extending it by ten years generates $367 trillion in value [629]. In relation to early diagnosis of aging, this section discusses recently developed techniques for the early identification of neurodegenerative disorders such as AD and Parkinson’s disease (PD). AD is the most common cause of dementia and is a serious health problem that is expected to increase threefold by 2050 [630]. Early diagnosis of cognitive decline is crucial for implementing effective treatment strategies to mitigate disease progression. This can help alleviate symptoms and improve quality of life [630,631]. This section encompasses biomarkers, the significance of assessing cognitive function, MRI-based techniques, AI and ML models, and brain age prediction.

### 6.1. Key Biomarkers and Pathological Changes

#### 6.1.1. Fluid-Based Biomarkers

When compared to CSF biomarkers and PET imaging, blood-based biomarkers offer advantages such as simple sampling, low invasiveness, and cost-effectiveness, making them the most promising early screening technology for AD [632]. Neurofibrillary Tangle (NfL) is a protein that is an important component of the cytoskeleton of neurons. It is released into CSF and plasma upon neural damage, leading to elevated levels in these bodily fluids. Plasma NfL levels were significantly higher in mild cognitive impairment (MCI) and AD groups compared to healthy controls, with the highest concentrations observed in the MCI group. This suggests that NfL levels begin to rise before the onset of significant cognitive decline [633,634]. In AD, abnormal aggregation of phosphorylated tau (p-tau) forms insoluble clumps, leading to synaptic dysfunction and brain cell death [635]. The subjective cognitive decline (SCD) group had higher plasma p-tau_181_ and plasma GFAP levels than the normal control (NC) group [632]. GFAP is an inflammatory molecule released by astrocytes and microglia, signaling neuronal damage due to reactive astrocytes [636]. A robust correlation between plasma p-tau_217_ levels and the severity of NfL in AD patients was demonstrated in a postmortem study [633]. miRNA-92a-3p, miRNA-486-5p, and miRNA-29a exhibited a significant correlation with the initial phases of AD. Also, levels of miRNA-483-5p were higher in people with MCI and AD than in NC [634]. Salivary levels of lactoferrin, and *P. gingivalis* IgG and specific urinary metabolites such as ApoC3 have been studied as non-invasive biomarkers for AD risk and disease diagnosis [634]. A study profiling human proteins throughout the entire 50-year lifespan identified 29 proteins that consistently increased in expression in six or more human tissues with age [637]. One of these, serum amyloid P component (SAP) is involved in amyloid formation, and its age-related increase may contribute to vascular aging, tissue damage, and inflammation. Amyloid protein aggregation has been identified as a common aging characteristic across human organs [637]. An intrinsic cellular aging clock (DNAm IC) derived from DNAm data based on the INSPIRE-T cohort has been constructed [316]. This clock has been validated as a more accurate predictor of mortality than existing epigenetic clocks and has been shown to be closely associated with aging indicators such as immune and inflammatory markers, functional and clinical outcomes, and lifestyle factors. Additionally, DNAm IC can be calculated using salivary samples, demonstrating its potential as a non-invasive alternative [316]. In contrast, a new proteomic aging clock measuring biological age using circulating plasma proteins has been developed [387]. Utilizing large-scale data from the UK Biobank (UKB), ProtAgeGap composed of 20 core protein models achieving approximately 95% of the age prediction performance of 204 protein models, demonstrated the ability to predict chronological age with high accuracy. This clock proved effective in predicting risks of age-related diseases, multiple morbidities, and mortality, showing comparable predictive power in other populations, including China (CKB) and Finland (FinnGen) [387].

#### 6.1.2. Cell Aging and Neuroinflammation

There is growing evidence that terminally differentiated neurons in the brain can re-enter a cell cycle-like process during neuronal aging and disease states. Cell cycle-related events were predominantly observed in excitatory neurons, and it was demonstrated that their eventual fate is likely to be cellular senescence [638]. In normal brain aging, the number of neurons reentering the cell cycle and aging neurons diminished, whereas in late-onset AD, these cells accumulated [638]. Neuroinflammation, a persistent low-grade inflammation linked to aging, demonstrates an enhanced pro-inflammatory response. It originates from intricate interactions among immune cells in the aging brain, dystrophic glial cells, and the impairment of the blood–brain barrier [639]. When autophagy is impaired, damaged cellular components accumulate, particularly in neurons and microglia. The accumulation of these toxic proteins exacerbates inflammation and contributes to neurodegenerative diseases (NDDs) [639]. Chronic inflammation worsens neurodegenerative diseases (NDDs) and eventually causes cognitive decline [639]. NDD like AD and PD are strongly linked to impaired proteostasis, characterized by protein aggregates that signify a failure in the cellular mechanisms governing protein synthesis, folding, and degradation [640].

#### 6.1.3. Cognitive Function Assessment

Dementia is an underdiagnosed syndrome, and there is a need to improve the early detection of cognitive decline. Early indicators of cognitive decline should be based on concerns from the individual or a reliable informant and can be objectified through standardized neuropsychological testing [641]. Repeatedly assessing learning using the multi-day learning curve (MDLC) can reveal disease-related memory decline in precliniciated with future cognitive decline relative to amyloid levels and standard measures [642]. In the preclinical AD stage, significant changes were found in tasks assessing various cognitive functions (e.g., episodic memory, semantic memory, language, and perception). Additionally, low cognitive performance in memory, attention, and executive function tasks predicts future cognitive decline even in global deterioration scale (GDS) stage 1 (i.e., when there is no subjective or objective cognitive decline) [641]. Gender-based research reveals significant gender-specific disparities in neurodegenerative biomarkers and their correlations with cognitive decline, especially concerning PD [643]. In both male and female PD patients, elevated CSF levels of IL-8 correlated with diminished Montreal Cognitive Assessment (MoCA) scores that identifies a sign of cognitive impairment, suggesting an association with cognitive decline. Elevated CSF levels of FABP in males and elevated MCP-1 and SCF levels in females were correlated with diminished MoCA scores, respectively [643]. Sensory deficits, including the deterioration of vision, hearing, taste, smell, and touch, may exacerbate the brain’s susceptibility to neuropathological alterations and increase the risk of AD onset [644]. Auditory processing deficits may worsen cognitive decline by elevating neural activity and resulting in tau accumulation [644]. Visual and tactile stimulation therapy has shown potential in alleviating cognitive decline in AD patients [644].

### 6.2. Neuroimaging Techniques

#### 6.2.1. T1-Weighted MRI and Structural Changes

T1-weighted MRI scans, which enhance signals from fatty tissue and suppress signals from water, are particularly useful for identifying cognitive decline and early signs of AD, as well as monitoring cognitive decline over time [645]. Aging causes various changes in the brain that can be seen with an MRI including brain volume changes, fluid-attenuated inversion recovery (FLAIR) white matter hyperintensity lesions, and changes in tissue properties like relaxation rate, myelin, iron content, and neuronal density [646]. Changes in brain volume with age include a decrease in gray matter and an increase in ventricle volume, both of which are linked to a decline in cognitive function [646]. Structural brain changes such as hippocampal atrophy and cortical atrophy, can happen long before the onset of cognitive symptoms [645]. The left parahippocampal gyrus and the left inferior lateral ventricle are two important parts of the brain that are linked to early diagnosis of AD. The latter, which often reveals structural changes such as enlargement and shape distortion in AD patients, showed the highest accuracy for diagnosing AD, with a maximum accuracy of 0.86 [630].

#### 6.2.2. White Matter Hyperintensity Lesions (WMH)

Vascular WMH are frequently observed in individuals aged 60 and above, with prevalence increasing with age. They are closely associated with cognitive decline, stroke risk, mental health, and brain structural deterioration [647]. WMH has been reported at high frequencies in young adults under 40 years of age. If these lesions represent an early form of subclinical cerebral small vessel disease (cSVD), it is important to investigate their emergence and progression to study pathophysiological correlates and associations with genetic, environmental, and behavioral risk factors [648]. Various tools exist for WMH segmentation, with DL-based methods increasing in number. However, there is a lack of validated automated methods in populations with low prevalence and low overall lesion burden [648]. The “SHIVA-WMH” detector, which was developed in the context of the SHIVA project (https://rhu-shiva.com/), is a 3D Unet-based tool optimized to detect the entire range of WMH severity, including very mild cases observable in young subjects. This tool demonstrated superior performance compared to existing methods in WMH segmentation [648].

#### 6.2.3. Other MRI Techniques

Tissue relaxometry indicates changes in tissue relaxivity with age and provides reference values for distinguishing between normal aging and pathological conditions [649]. Myelin content quantified by MRI increases with age and later decreases, and is associated with cognitive decline and changes in walking speed in the elderly. In AD patients, age-related myelin destruction is more pronounced [646]. Brain iron accumulation detected using magnetization susceptibility MRI techniques increases with age in specific brain regions and may contribute to neurodegenerative processes [646]. Neurofluid imaging using MRI techniques such as gadolinium-based contrast agents (GBCA) and diffusion MRI demonstrates age-related changes in the dynamics of CSF and interstitial fluid (ISF), which are crucial for brain health and waste removal [646]. HIV infection accelerates brain aging and disrupts glymphatic clearance pathways in the aging brain, which may explain motor and executive dysfunction in HIV-infected individuals [650]. The assessment of glymphatic performance was conducted using the DTI-ALPS method, which is based on diffusion imaging of MRI data. Healthy individuals exhibit higher glymphatic function at younger ages, which significantly decreases with age. However, HIV-infected individuals exhibit impaired glymphatic clearance function regardless of age [650]. Functional MRI (fMRI) analyzes brain activity using BOLD contrast images, and image quality has been improved by introducing spin echo pulses and enhancing magnetic field strength. This allows comparison of differences between active and resting states, developmental and aging states, and normal and damaged brains. However, large sample sizes are required for interpretation in cognitive research [651].

#### 6.2.4. Other Neuroimaging Data

Multimodal imaging is used for the diagnosis of AD, and T1-weighted MRI detects atrophy in the medial temporal lobe and other areas [652]. Position Emission Tomography (PET) provides information on functional changes in the brain, such as glucose metabolism (FDG-FET), amyloid plaque deposition, and tau pathology [652]. These various imaging techniques can contribute to the early diagnosis of AD by elucidating its characteristics and understanding the association between protein aggregates and pathophysiology [652]. Electroencephalography (EEG) is used to analyze brain activity patterns by recording synaptic activities and identifying changes associated with AD [653]. The diagnosis of AD is made on the basis of characteristic patterns such as a decline in oscillatory activity and a reduction in complexity levels in EEG signals [653]. Retinal imaging is being studied as a potential early diagnostic tool for AD by assessing retinal vascular thickness and characteristic changes noninvasively [654,655]. A study that employed mesoscale microscopy technology and in vivo imaging to elucidate the detailed changes occurring in the brain vascular network of aged mice revealed that vascular dysfunction, particularly impaired blood–brain barrier (BBB) function and reduced pericytes density, may precede neuronal damage associated with NDD and other forms of dementia [656]. This finding suggests that such alterations could function as early indicators or therapeutic targets.

#### 6.2.5. Key Datasets

The Open Access Series of Imaging Studies (OASIS) is a project aimed at making neuroimaging data sets of the brain freely available to the scientific community [657]. OASIS-Cross-sectional and OASIS-Longitudinal have been used in neuroanatomical research and the development of segmentation algorithms. OASIS-3 includes long-term multimodal imaging, clinical, cognitive, and biomarker data from subjects with normal aging and AD, while OASIS-4 provides MR, clinical, cognitive, and biomarker data from patients with memory problems [657].

The AD Neuroimaging Initiative (ADNI) is a long-term, multi-center research dataset aimed at developing biomarkers for AD. It includes MRI, PET, and cognitive test data from patients with AD, CN (cognitively normal), and MCI [658]. The Internet Brain Segmentation Repository (IBSR) dataset has been developed for the purpose of evaluating brain image segmentation, and it comprises 20 real T1-weighted MRI scans as well as expert manual segmentation results [659]. The model consists of slices at various resolutions and volumes of 256 × 256 × 128, and also provides segmentation of 32 non-cortical structures [660].

The Human Connectome Project (HCP) is a large-scale international research project aimed at mapping the brain’s structural and functional connectivity in great detail [661,662]. It collected a wide range of brain imaging data, including structural MRI, diffusion MRI (dMRI), resting-state and task-based functional MRI (fMRI), as well as behavioral and cognitive data from approximately 1200 healthy adults [661,663,664]. The HCP provides standardized protocols and high-resolution images (e.g., dMRI 1.25 mm, fMRI 2 mm), as well as the ability to analyze genetic factors, including data from twins and families. This information is used in a variety of applications, including brain network analysis, cognitive function research, disease mechanism exploration, and predicting brain age [665,666,667]. An open platform makes data freely available, which is extremely useful in neuroscience and AI research [668].

The Cambridge Centre for Aging and Neuroscience (Cam-CAN) is a substantial public neuroscience dataset aimed to investigate the impact of aging on cognitive functions and the associated neural mechanisms [669,670]. It collected high-quality brain imaging data, including structural MRI, resting-state and task-based functional MRI, and MEG data, from 700 healthy adults aged 18 to 87 [669]. It also has information from a variety of cognitive and behavioral tests, including tests of memory, attention, and executive function [671]. This data, obtained through standardized protocols, is utilized to examine changes in brain structure and function, cognitive decline, and the mechanisms associated with age-related diseases [672,673]. Cam-CAN data supports the development of neuroimaging biomarkers for cognitive aging and early detection of decline [672]. Brain age prediction using multimodal neuroimaging data achieved high accuracy in distinguishing between chronological age and brain-predicted age [674]. Researchers can get free access by filling out an application.

### 6.3. Integration of AI

A range of data types have been leveraged for the early diagnosis and prediction of AD, with AI and ML/DL) models playing a pivotal role in the analysis of this data to discern subtle patterns indicative of the disease.

#### Various ML/DL Models and Performance

Traditional aging markers like SA-β-gal, p16, p21, HMGB1, and Lamin B1 for identifying SnCs have limitations in terms of variability and lack of consistency. However, the ML-based nuclear morphometric pipeline (NMP) demonstrated the quantification of nuclear features at single-cell resolution to generate an “aging gradient”, enabling objective and precise evaluation of SnCs [675]. NMP utilizes unsupervised learning, making it applicable to various aging-inducing factors, cell types, and tissue environments, and has demonstrated that major SnCs fractions change with age such as fibroadipogenic progenitors (FAPs) in young regenerating muscle versus satellite cells in aged muscle [675].

To facilitate early diagnosis of AD, 146 brain-based biomarkers (BBBMs) and 12 ML algorithms based on ADNI data were used [676]. The results indicated that Linear Discriminant analysis (LDA), Naive Bayes, and SVM showed promising performance, and an ensemble model integrating these algorithms was developed. Four BBBMs such as Immunoglobulin M (IGM), Placenta Growth Factor (PLGF), Serum Glutamic Oxaloacetic Transaminase (SGOT), and Alpha-1-Microglobulin (A1Micro) were found to be critical for early detection of AD, demonstrating a SN of 92.86% and SP of 82.35%, thereby validating that BBBMs and ML are effective diagnostic tools for clinical practice [676].

A study comparing traditional ML algorithms, including SVM, decision trees, and K nearest neighbor (K-NN), and DL techniques such as multilayer perceptron (MLP) and CNNs for early diagnosis of AD showed that ML is useful with limited data, while DL demonstrates superior performance with abundant data, suggesting the possibility of early and accurate diagnosis [677]. The primary strength of CNNs is their proficiency in automatic feature extraction from MRI images due to their ability to learn complex patterns in complex data [678]. CNNs have demonstrated excellence in MRI image processing, showing the highest accuracy in diagnosing AD [679]. However, their efficacy may be constrained by issues such as gradient vanishing or explosion, which can occur in deeper layers of the network [678,680]. Residual networks such as ResNet-50 demonstrated CNNs’ diagnostic accuracy of AD further by addressing vanishing gradient issues, allowing for deeper architectures and better feature extraction [681].

To overcome the limitations of existing large-capacity TL models, a lightweight CNN model integrating a squeeze and excitation (SE) block and an innovative Avg-TopK pooling layer has been reported [682]. Additionally, this model utilized SMOTE (Synthetic Minority Over-sampling Technique) to address data imbalance issues. This technique generates synthetic data for the minority class to balance the dataset and helps the model improve its prediction performance for the minority class. The CNNs model designed in this way achieved an improved accuracy of 99.84% in AD diagnosis [682].

A hybrid model combining features extracted from the ResNet-18 architecture and traditional image processing algorithms, including Local Binary Pattern (LBP), Discrete Wavelet Transform (DWT), Gray Level Co-occurrence Matrix (GLCM), demonstrated an accuracy of 99.8% and SP of 100% in the diagnosis of MRI images for early detection of AD [683]. Deep feature maps extracted by ResNet-18 were combined with traditional feature extraction algorithms such as LBP, DWT, and GLCM to generate rich feature vectors. These integrated features were classified using feedforward neural networks (FFNN) [683].

Through the utilization of previously trained models, TL provides the benefit of lowering the costs associated with training while simultaneously enhancing performance with a limited amount of training data [684]. The implementation of a fine-tuned CNN model was demonstrated by applying TL to CNN architectures (AlexNet, GoogleNet, and MobileNetV2) and combining these architectures with several solvers including Adam, Stochastic Gradient Descent (SGD), and Root Mean Square Propagation (RMSprop) [684]. This model achieved a high AD classification accuracy of 99.4% on the Kaggle MRI dataset and 98.2% on the OASIS dataset. These results demonstrate that the model has the potential for the early and accurate diagnosis of AD.

A new DL architecture combining a 3D convolutional block attention module (3D CBAM) with a video Swin Transformer in a 3D CNN, named 3D-CNN-VSwinFormer, demonstrated 92.92% accuracy and 0.9660 AUC in distinguishing AD patients from cognitively normal (CN) individuals by extracting local features from 3D MRI data and obtaining comprehensive information on brain structural changes through multi-scale features [685]. This model effectively avoided the data leakage issues that can occur in 2D MRI slice-based analyses while also capturing global spatial information about the brain [685]. Furthermore, it demonstrated superior computational efficiency.

DL can improve diagnostic accuracy by combining data from multiple sources, such as MRI, EEG signals, retinal images, cognitive test results, DNA analysis, blood-based biomarkers, and voice recordings [686]. For example, a multi-modal DL framework that utilizes the entire ADNI series, including T1-weighted MRI and 18F-FDG PET from the ADNI1 dataset, as well as Aβ PET and tau protein PET from ADNI2 and ADNI3 datasets, achieved a balanced accuracy of 1.00 in the AD versus CN task and 0.76 in the MCI versus CN task [687]. A multimodal classification model that combines clinical, cognitive, MRI, and EEG data has been proposed. This model uses the TimesBlock module to capture EEG time patterns and then fuses MRI spatial information and EEG time data via cross-modal attention to improve the distinction between AD, MCI, and CN groups [688]. Furthermore, the first AD classification dataset encompassing all three modalities has been constructed, increasing the potential for early detection and intervention [688]. A multimodal learning study incorporating MRI-derived brain atrophy, EEG irregular patterns, behavioral assessments, and amyloid beta (Aβ) markers demonstrated that a reduction in Aβ1-42/Aβ-40 correlates with frontal-temporal lobe atrophy and anomalies in EEG signal complexity and information transfer [689]. A random forest model that used structural, electrophysiological, and clinical data was able to accurately predict Aβ ratios 91.6% of the time. This suggests that it could be a useful and cheap way to diagnose problems early [689].

Given the importance of sleep disorders in AD, a multi-modal ML approach has been proposed to non-invasively predict core CSF biomarkers of AD using polysomnography (PSG) and clinical and blood data [690]. The study found significant correlations between sleep-related variables from PSG and the levels of AD CSF biomarkers. Poorer sleep quality and shorter sleep duration were associated with lower Aβ42 levels. The most effective predictors of CSF p-tau and t-tau levels were quantitative PSG features (PSGVAR), conventional PSG parameters, and clinical variables (ALL subset) [690]. These findings suggest that specific quantitative PSG features, such as sleep EEG, could serve as potential early biomarkers for AD. Furthermore, while CSF collection via lumbar puncture is invasive and expensive, making it unsuitable for widespread screening, PSG offers a less invasive alternative for detecting early AD markers [690].

To address the issue of the “black box” nature of DL models, which makes decision-making processes opaque and difficult to understand, explainable AI (XAI) frameworks such as Gradient-weighted Class Activation Mapping (Grad-CAM), Local Interpretable Model-Agnostic Explanations (LIME), SHapley Additive exPlanations (SHAP), Layer-wise Relevance Propagation (LRP), and Saliency Map have been developed [691]. For example, Grad-CAM improves model interpretability and clinician confidence by visually demonstrating that the model focuses on specific areas of the brain (e.g., parietal lobe, cerebral cortex, Broca’s area, medial temporal lobe) when making diagnostic decisions [691]. SHAP quantifies each variable’s contribution to the model’s prediction using numerical features such as brain volume and metabolic rate and points out the basis of predictions using numerical quantification or rule-based explanations, thereby increasing clinician confidence and encouraging clinical application of the model [691].

### 6.4. Brain Age Prediction and Brain Age Gap

The field of brain age prediction (BAP) is an emerging discipline, employs neuroimaging data and ML algorithms to estimate the biological age of an individual’s brain in relation to their chronological age [692]. This approach has attracted considerable attention as a potential biomarker for brain health, NDD, and cognitive aging [693]. The BAG is the difference between the predicted age and chronological age, reflects the rate at which the brain is aging relative to expectations [692]. The field encompasses a wide range of neuroimaging techniques, ML methodologies, and clinical applications.

#### 6.4.1. Importance of BAP and BAG

Brain age refers to the biological age of the brain, estimated using MRI data [258]. MRI is important in the diagnosis of NDD and plays a key role in distinguishing normal aging from dementia [694]. Structural MRI is helpful in identifying early-stage AD by analyzing brain structural changes, such as hippocampal atrophy [695]. BAG is considered a promising indicator of brain health [258]. It provides indicators of age-related brain functions [258]. Identifying the genetic elements that contribute to BAG will enable the development of genetically validated targets and treatment strategies that may decelerate or potentially reverse brain aging, resulting in a prolonged lifetime [258].

#### 6.4.2. Neuroimaging Techniques and Their Characteristics

Structural MRI, especially T1-weighted imaging, is the predominant method employed for predicting brain age [696]. Structural characteristics by T1 weighted MRI reflect age-related alterations in gray matter volume, cortical thickness, and brain morphology [697]. Regions exhibiting the most pronounced age-related alterations encompass the frontal and temporal cortices, with periventricular areas being especially important for age prediction [698]. The MAE tends to be lower in samples characterized by a narrower age range, as this reduces errors when the predicted brain age closely aligns with the mean chronological age of the population being studied [699]. The UK Biobank, ADNI, and Neurodegenerative Disease Cohort Study (NACC) datasets were employed to systematically assess the efficacy of these models as age prediction instruments for healthy individuals and their viability as biomarkers for diverse NDD [700]. The findings indicated that a penalized linear ML model utilizing Zhang’s BAP approach [701], which integrates age bias correction methods commonly applied in BAP models, attained highly precise BAP with MAE of under one year in external validation [700]. This model exhibited stable predictive performance across different age groups and subgroups, including race and MRI machine/manufacturer [700].

Recent studies demonstrate the enhanced efficacy of multimodal approaches compared to single-modality predictions [702]. Roibu et al. demonstrated that 57 distinct MRI contrasts, including structural, susceptibility-weighted, diffusion, and functional MRI, were capable of predicting brain age [702]. Ensemble methods that integrate multiple modalities offered greater accuracy compared to individual modalities and stronger correlations to non-imaging measurements [702].

Diffusion Tensor Imaging (DTI) offers distinct insights into alterations in white matter microstructure associated with aging [156,703]. Wang et al. proposed a 3D CNN model and trained it using fractional anisotropy (FA) data collected from six public datasets (*n* = 2406, age = 17–60) to estimate brain age, achieving robust BAP performance with a MAE of 2.785 years and a correlation coefficient of 0.932 [703].

PET imaging provides additional functional data for predicting brain age [704]. Dörfel et al. showed that 5-HT2AR binding assessed via PET exhibited performance similar to gray matter volume (MAE = 6.63 vs. 6.95 years), while the combining both measures enhanced prediction to MAE = 5.54 years [704]. This demonstrated the importance of integrating molecular imaging biomarkers with structural assessments.

#### 6.4.3. ML/DL Performance of BAP

Traditional ML methods require manual feature extraction, which can be time-consuming and subjective [705]. However, it exhibits accelerated learning on small to medium-sized datasets and necessitates less computational power, making it appropriated for environments with constrained hardware resources [706]. ML models have shown promising results when combined with neuroimaging data in the early stages of diagnosing neurodegenerative diseases, particularly AD [707,708]. Xiong et al. evaluated the impact of ML algorithms and image modalities for predicting brain age in adults aged 44.6 to 82.3 years old [709]. Among six ML algorithms (Lasso, relevance vector regression (RVR), support vector regression (SVR), extreme gradient boosting (XgBoost), category boost (CatBoost), and multilayer perceptron (MLP)), Lasso achieved the lowest MAE in both uni- and multi-modality predictions, demonstrating it as the most accurate ML for BAP. Specifically, for multimodality data, Lasso recorded an MAE of 2.741 years [709]. Meanwhile, the ensemble model achieved an MAE of 2.338 years, outperforming Lasso, a single ML when computational efficiency was not a primary concern [709]. In addition, the study demonstrated that the choice of imaging modality, particularly T1-weighted MRI and DWI, plays a more significant role in prediction accuracy than the choice of ML algorithm [709]. While several ML algorithms can effectively predict brain age, the specific choice of algorithm significantly influences the observed associations between BAG and cognitive functions, particularly executive function and emotion processing [710]. SVR, RVR, and Gaussian Process Regression (GPR) demonstrated better performance in identifying BAG-Cognitive associations compared to other ML algorithms, emphasizing the critical role of algorithm selection in brain age research [710].

CNNs and other DL architectures have demonstrated exceptional efficacy in extracting intricate patterns and anomalies from imaging data for BAP. The Simple Fully Convolutional Network (SFCN) architecture has shown particular success, with studies reporting MAE values of 2.14 in UK Biobank data (*n* = 14,503) [711].

A number of DL models, including SFCN, ResNet, EfficientNet, VGGNet, DenseNet, GLT, and 3D-ViT, have been trained on T1-weighted MRI data from the UK Biobank [258]. Among them, the 3D-ViT (three-dimensional vision transformer) demonstrated the best performance, displaying an MAE of 2.64 and a Pearson correlation coefficient of 0.90 in 5-fold cross-validation [258].

The 3D DenseNet-169-based model was effectively trained using interpolated 2D axial T1-weighted MRI scans derived from research-grade 3D T1-weighted MRI scans [712]. The model achieved an MAE of 1.53 years with a high correlation to chronological age (Pearson’s r = 0.996). Furthermore, the model’s ability to achieve an MAE of 2.73 years (after age bias correction) on clinical grade 2D axial MRI scans, and even lower (2.23 years) with an ensemble approach, signified its high accuracy for practical application. For AD patients, the model predicted an MAE of 3.10, which was significantly higher than that of CU subjects (*p* < 0.001) [712]. This represents a significant improvement over existing models that could not accurately predict brain age using clinical grade 2D axial MRI scans because they have been trained using research-grade 3D T1-weighted MRI scans [713]. The study demonstrated that the 3D DenseNet-169-based model can accurately predict brain age from routine clinical 2D MRI scans and effectively identify increased brain age gaps associated with NDD such as AD, enhancing the technology’s broad clinical applicability [712].

Recent innovations include transformer-based approaches and hybrid CNN-transformer models [714]. The ds-FCRN (dual-stream fully convolutional residual networks) combined with transformer-based global–local feature learning achieved competitive performance while providing enhanced interpretability. In a test set comprising 3276 healthy subjects (mean age 64.15 ± 7.45 years, 1561 males), the 3D ds-FCRN model achieved an MAE of 2.2 years in BAP, outperforming existing models on the same dataset [714]. A Dense Transformer Foundation Model with Mixture of Experts (DenseFormer-MoE), which integrates dense convolutional network, a Vision Transformer, and MoE, was introduced, and improved BAP accuracy by effectively learning both local and global feature representations [715]. Pre-training the DenseFormer backbone using a masked autoencoder enabled the acquisition of robust and generalizable feature representations through the masking and reconstruction of input patch segments. This significantly enhanced performance, particularly in medical imaging, where labeled data is limited [715]. A global–local attention network model for BAP using multimodal neuroimaging data, including sMRI and dMRI, demonstrated an MAE of 2.44 years, showing a reduced MAE by 10–76% compared to uni-modality models [674]. In particular, this multimodal model revealed unique associations between predicted brain age and behavioral measures, such as walking endurance and loneliness, in the HCP dataset, which were not detected by chronological age alone. Specifically, higher predicted brain age was associated with lower walking endurance and loneliness scores, and higher odor identification ability and conscientiousness scores [674]. In the Cam-CAN dataset, brain age and chronological age exhibited a similar correlation with behavioral measures [674].

Quantum deep learning (QDL), an emerging DL sub-field, was assessed for brain data learning tasks [716]. A statistical analysis of brain tumor classification studies (*n* = 16) revealed that the QDL model achieved an average accuracy of 0.9701, slightly outperforming the classical model, which had an average accuracy of 0.9650. Similar trends were observed across multiple domains, including AD, stroke lesion detection, cognitive state monitoring, and BAP (MAE 3.302 vs. 3.31, RMSE 4.083 vs. 4.28) [716]. QDL models may offer advantages such as accelerated processing, enhanced performance, improved generalization, and more efficient parameter utilization. In addition, they could aid in the analysis of intricate neural data by using feature representations in high-dimensional quantum spaces. While QDL has great potential in neuroinformatics, it has fundamental limitations in data encoding, the need for robust and task-specific circuit design, current constraints in quantum hardware, and difficulties with model optimization and interpretability [716].

## 7. Point-of-Care Applications

Point-of-Care (POC) diagnostic technology plays a pivotal role in advancing healthcare services and precision medicine by enabling diagnosis and monitoring close to the patient [717]. POC devices, developed through advancements in nanotechnology, molecular diagnostics, AI, and smartphone integration, are revolutionizing healthcare by providing rapid, accurate, and highly accessible diagnostics [717,718,719]. This substantially influences the early detection of diseases like cancer and age-associated diseases, resulting in improved patient outcomes and increased efficiency within the healthcare system. In addition, it contributes to improving access to medical services, particularly in environments with scarce medical resources or in remote areas [717].

This review examines key technologies used in POC devices, clinically approved and developing point-of-care sensors for the diagnosis of cancer and geriatric diseases, emphasizing significant advancements in biosensor technology, and emerging innovations anticipated to transform early detection and monitoring.

### 7.1. Recent Developments for Enhancing Biosensor Performance

#### 7.1.1. Nanotechnology (Nanomaterials and Nanostructures)

Nanomaterials play a crucial role in enhancing biosensor performance. Nano-structures contribute to lowering detection limits, reducing SN to contamination, and increasing mechanical strength [720,721]. Noble metal NPs (NM NPs) (e.g., gold, silver) and quantum dots (QDs) play a crucial role in signal amplification during POC biosensor development [722]. Gold NPs (AuNPs) generate colorimetric signals five orders of magnitude stronger than organic dyes [723,724]. QDs exhibit high resistance to photobleaching, making them advantageous for long-term and in vivo applications [725]. Carbon nanotubes (CNTs), and graphene are widely used. Combined with various sensing platforms, they enable rapid, cost-effective, and reliable POC diagnostics required for cancer diagnosis [720,726].

#### 7.1.2. Wearable Sensors

Wearable biosensors are used to continuously and non-invasively monitor human physiological parameters through bodily fluids like sweat, tears, and saliva [727]. Designed to be flexible and integrated into multiple networks, enabling continuous biomarker monitoring. Integration with AI systems contributes to predictive model generation and remote clinical decision support [727]. Wearable platforms like smart contact lenses, sweat patches, and digital wristwatches provide valuable insights into an individual’s physiological state and health status [728]. Wireless smart contact lenses demonstrated potential for diabetes diagnosis and treatment [729,730,731], while wearable sweat-based blood glucose monitoring devices also measure blood sugar non-invasively [732,733].

#### 7.1.3. Smartphone Integration

Smartphones have become a core component of POC diagnostic devices [734]. They leverage already widely available devices to reduce the cost of additional specialized equipment and provide functions such as data processing, image analysis, and result transmission [735]. Smartphones can be transformed into low-cost, high-precision colorimeters through image processing and data analysis capabilities, and are utilized for diagnosing various diseases [735]. They connect to POC devices via Bluetooth, Wi-Fi, or USB. Smartphones serve as readers for portable diagnostic devices, processing and quantifying images such as lateral flow immunoassay (LFIA) results [719], coffee ring biosensor patterns [726], and fluorescent signals [736].

#### 7.1.4. Integration of AI in POC Testing (POCT)

AI and ML algorithms are integrated into POC devices to enhance diagnostic accuracy and efficiency [719]. They interpret complex multivariate patterns, generate predictive models, and automate image/signal analysis. AI can reduce the risk of misdiagnosis and lower dependence on skilled medical personnel, improving healthcare accessibility in resource-poor countries and remote areas [719]. AI models are used for image processing (e.g., cell type classification, anomaly detection), quantifying bioanalytical results, and predicting disease, achieving high accuracy and SN in diagnosing specific cancers (e.g., BC, LC) [717]. The convergence of these diverse technologies is expected to drive the evolution of POC devices into more accurate, affordable, and user-friendly forms, playing a crucial role in enhancing healthcare accessibility and delivering patient-centered medical services. Recently, a DL model combined multi-ultra-sensitive coffee ring biosensor has been proposed [726]. The system couples a generative neural network with a CNN to quantitatively analyze complex biomolecular patterns directly from smartphone-captured images. This AI-driven approach enabled highly precise protein detection down to 3 pg/mL within 12 min, eliminating the need for bulky analytical instruments [726]. By training the DL model on diverse optical interference and pattern datasets, the platform achieved a broad, multi-dimensional detection range for biomarkers related to sepsis (procalcitonin), COVID-19 (N-protein), and cancers (CEA, PSA). When validated using saliva samples, its signal-to-noise ratio exceeded that of conventional LFIA by more than twofold, demonstrating the transformative potential of AI-assisted biosensing for rapid, low-cost, and high-accuracy early disease diagnostics [726].

### 7.2. Microfluidics Systems and Chip-Based Devices

#### 7.2.1. Concept and Features of Microfluidics Systems and Chip-Based Devices

Microfluidics, also known as lab-on-a-chip or micro total analytical systems manipulates minute fluids within microchannels [737]. MDs facilitate sample pretreatment, separation, dilution, mixing, chemical reactions, detection, and product extraction using minimal sample volumes within a singular, compact system. This enhances the speed and efficiency of analysis, reduces reagent consumption, and automates the entire process, thereby minimizing human intervention [504,506,738]. Due to their exceptional portability and user-friendly interfaces, MDs enable even inexperienced users to conduct analyses independently [738]. Furthermore, they provide multiplexing capabilities, which enable the simultaneous detection of multiple biomarkers in a single analysis, thus facilitating a more precise evaluation of the disease state [739].

#### 7.2.2. Application of Microfluidic System for Cancer Diagnosis in POCT 

An ISFET (Ion-Sensitive Field-Effect Transistor) sensor-based LoC (Lab-on-Chip) device was developed to detect cervical cancer by amplifying human papilloma virus (HPV) DNA and human TERT mRNA through the loop-mediated isothermal amplification (LAMP) assays [740]. This portable device effectively detected cervical cancer from biopsy samples, demonstrating the potential for large-scale cervical cancer screening in resource-limited settings. The HPV 16/18 E6 oncoprotein has been recognized as a potential biomarker for a more precise early diagnosis of cervical cancer [741]. The OncoE6™ Cervical Test (Arbor Vita) is a fast, simple lateral flow method that detects HPV16/18 E6 oncoproteins with high SP and detects high-grade cervical lesions. This technology could enable decentralized screening of hard-to-reach populations if it supports self-collection samples [742]. The diagnostic performance of the OncoE6™ cervical test kit for cervical precancer and cancer in the Amhara Region of northwestern Ethiopia was evaluated, revealing a SN of 57.14% and a SP of 98% in identifying cases of cervical precancer and cancer [743]. Its high SP renders it an appropriate screening choice in areas with high HPV prevalence and large high-risk populations.

He et al. developed a microfluidic chip that combines centrifugation pretreatment with a nano-sensing film to rapidly and accurately detect VEGF165, a crucial factor in angiogenesis and cancer progression, in clinical blood samples [744]. Plasma was separated and conveyed to the electrode within 5 min, where a dual-aptamer-based sandwich approach was employed. The Apt2 film on the Au electrode surface and the Apt1/Au NP nanoprobe selectively and sensitively bind to VEGF165 in plasma. This sensor exhibited a detection limit of 0.67 pg/mL and a broad linear range of 1 pg to 10 ng, suggesting its utility as a POC platform for the early clinical diagnosis and prognostic monitoring of VEGF165 [744].

Using nanoporous gold membranes and antibody-conjugated nanorods, a new MD demonstrated rapid, ultrasensitive isolation and in situ detection of LC-specific exosomes in urine [745]. Validated in patient samples, it distinguished early-stage cancer with high purity and low detection limits, indicating potential adaptability to other tumor types.

Inspired by the hunting efficiency of octopuses, a deterministic lateral displacement (DLD) microfluidic chip incorporating aptamer-functionalized nanospheres (AP-Octopus-Chip) has been developed [746]. The multivalent aptamer-antigen binding enhanced affinity by 100-fold and capture efficiency by over 300% compared with a monovalent aptamer-modified chip [746]. Captured cancer cells can be released with up to 80% efficiency and 96% survival rate, making it compatible with mutation detection and CTC culture. CTCs were successfully detected in all cancer samples [746]. In another similar study, aptamers were functionalized onto leukocyte membrane nanovesicles and attached to a microfluidic chip, implementing a fluidic multivalent nanointerface [747]. This biomimetic interface maximized ligand-receptor interactions, achieving a 7-fold higher capture efficiency and a 97.6% CTC survival rate compared to monovalent aptamer chips. Furthermore, it minimized background blood cell adsorption and successfully detected CTCs in 17/17 actual patient samples, demonstrating its clinical applicability [747]. Inspired by the predatory strategy of plate corals, a biomimetic CTC capture nanoprobe (MNPA-TCMMGO) has been developed by conjugating a multi-affinity aptamer to magnetic graphene oxide coated with tumor cell membranes [748]. The cell membrane provided homotypic tumor cell SP, while the multivalent aptamer significantly enhanced binding affinity and probability, enabling sensitive and specific capture of rare CTCs. Furthermore, it exhibited robustness and biocompatibility while maintaining captured cell viability, demonstrating promise for downstream analysis and clinical application [748].

Paper possesses unique properties such as capillary-based liquid transport, ease of design, and a high surface area-to-volume ratio, making it a widely used material in POC devices for its cost-effectiveness and ease of production and operation [749]. Paper-based MDs (µPADs) can be produced at low cost, providing accessibility to a wide range of patients. They can perform analysis on various bodily fluid samples (urine, saliva, blood, tears, etc.) [749]. Paper-based assays detecting HPV E7 tumor protein was developed, which assay operate without instruments through a simple 5-step process [750]. The HPV E7 paper-based assay demonstrated 95% accuracy in clinical performance when compared to histopathologic diagnosis of cervical intraepithelial neoplasia grade 2 or more severe (CIN2+) [750]. With additional clinical validation, they could enable highly specific POC testing in resource-limited settings [750]. A 3D platform has been developed to concurrently detect tumor markers such as CA125 and CEA in blood samples, as well as a wireless point-of-care system for detecting neuron-specific enolase (NSE) [749]. A label-free μPAD sensor for accurate electrochemical detection of PSA was developed using wax screen printing and gold NPs (AuNPs)/reduced graphene oxide (rGO)/thionine (THI) nano composites, demonstrating sensitive detection of PSA down to 10 pg/mL (linear range 0.05–200 ng/mL) [751]. This sensor showed the potential to provide a new platform for low-cost, sensitive POC diagnosis of PC [751].

#### 7.2.3. Application of Microfluidic System for Age-Related/Chronic Diseases Diagnosis in POCT 

A 3D μPAD capable of simultaneously measuring multiple cardiac biomarkers in blood, including heart-type fatty acid-binding protein (H-FABP), cardiac troponin I (cTnI), and copeptin, has been developed [752]. A high-performance optical sensing platform equipped with a passive blood filtration microfluidic cartridge for detecting CRP has also been developed, proving useful for monitoring patients with chronic diseases [753]. A microfluidic POC system integrated with a field-effect transistor sensor array capable of simultaneously analyzing multiple CVD biomarkers—including CRP, N-terminal pro-B-type natriuretic peptide (NT-proBNP), cTnI, and fibrinogen—has been developed, demonstrating promise for CVD monitoring [754]. The sensor assessed biomarkers over an extensive dynamic range: NT-proBNP (0.1–10,000 pg/mL), fibrinogen (50–1000 mg/dL), cTnI (0.1–10,000 pg/mL), and CRP (0.5–9 mg/L) [754].

A portable, low-cost sensor designed to measure serum phosphate in patients with advanced CKD and dialysis patients using a single drop of blood (less than 60 μL) was introduced [755]. This sensor combines a paper-based microfluidic platform with a smartphone reader and exhibited a strong correlation (r = 0.95) with laboratory tests in clinical trials [755]. This affordable and easy-to-use system can accurately measure serum phosphate levels in just 45 min in POC, allowing patients to monitor it more frequently at home [755].

Saliva is progressively utilized for diagnostic purposes. Saliva is an optimal non-invasive biological fluid for biomarker detection due to its capacity for large-volume collection and its precise reflection of blood analyte concentrations [756]. A cobalt-MOF (Co-MOF) modified carbon cloth/paper (CC/Paper) hybrid button-sensor was developed as a portable, robust, and user-friendly electrochemical analytical chip for the nonenzymatic quantitative detection of glucose, a classical biomarker of diabetes [757]. The flexible Co-MOF/CC interface, characterized by numerous catalytic sites and a large specific surface area, enabled the Co-MOF/CC/paper hybrid button sensor to accurately quantify glucose with high SN and selectivity in saliva as well as serum and urine, without the necessity of enzymes [757]. The paper-based chip demonstrated affordability, exceptional portability, and durability, rendering it appropriate for home healthcare and rapid POC diagnostics [757].

A POC diagnostic sensor utilizing paper-based fluorescent vertical flow analysis (fxVFA) successfully quantified three acute cardiac injury biomarkers (myoglobin, CK-MB, H-FABP) within 15 min using 50 µL of serum per patient [758]. Neural network-based analysis achieved a detection limit below 0.52 ng/mL, linearity above 0.9, and a coefficient of variation below 15%. Its low-cost, portable design demonstrated enhanced diagnostic accessibility even in resource-limited settings [758].

### 7.3. Electrochemical Biosensors in POCT

#### 7.3.1. Concept and Features of Electrochemical Biosensors

Electrochemical sensors are gaining considerable attention in the POC field due to their high SN, miniaturization, and cost-effectiveness [759]. They are used in various applications ranging from disease diagnosis to health status monitoring. These sensors primarily detect biomarkers by immobilizing biomolecular recognition elements such as enzymes, antibodies, or aptamers onto electrode surfaces [759]. Electrochemical sensors provide rapid and quantitative results by directly reflecting material concentrations through various electrical signal changes [759]. Voltammetry is appropriate for trace analysis owing to its exceptional SN and precision, whereas amperometry facilitates rapid and accurate measurements by monitoring current responses to variations in analyte concentration [760]. Impedance measurement offers instantaneous assessment of electrode-analyte interactions, elucidating reaction rates and mechanisms [760]. Potentiometric titration provides a straightforward and expedient technique for determining concentration by measuring potential variations. These varied electrochemical methods concurrently fulfill high SN, cost-efficiency, and speed, rendering them ideal quantitative analytical instruments for biosensors [759].

Through microelectrode technology, they can maintain high SN even with very small sample volumes without significantly affecting measurement SN [761].

#### 7.3.2. Application of Electrochemical Biosensors for Cancer Diagnosis in POCT 

Exosome miRNA patterns reflect dysregulation in tumor cells, making them promising for cancer diagnosis. For examples, a photoelectrochemical (PEC) biosensor based on the hybrid nanomaterial MoS_2_@Ti_3_C_2_ was developed [762]. This biosensor exhibited high SN for miRNA detection, attributed to the superior electron transfer capability of Ti_3_C_2_. The detection limit for miR-92a-3p, an exosome miRNA linked to CRC, was 0.27 fM, with a linear detection range extending from 1 fM to 100 nM [762]. A significant characteristic was its selectivity, allowing for accurate differentiation between miR-92a-3p and other RNA molecules with erroneous sequences. In addition, by accurately detecting miR-92a-3p concentrations in exosomes from both patient groups and healthy controls, PEC biosensor demonstrated clinical applicability [762]. Another PEC immunosensor for detecting CEA utilized an organic-inorganic heterojunction photoactive material by combining the organic semiconductor Y6 with AuNPs [763]. ZnPO_4_ NPs with horseradish peroxidase (HRP) embedded was used as signal probe. The insulating film generated through HRP catalysis inhibited the photoelectrochemical current intensity, facilitating sensitive and selective detection of CEA within a linear range of 1 pg/mL to 1 ng/mL, with a detection limit of 0.5 pg/mL [763].

A screen-printed gold electrode-based electrochemical miRNA detection platform was developed [764]. Methylene blue-modified DNA sequences immobilized on the electrode enabled targeted detection of miRNA-21, achieving an LoD of 2 nM using square-wave voltammetry. After optimization for pH, interfering substances, and NaCl concentration, high selectivity, reproducibility, and nM-level performance were confirmed in urine samples [764].

A CRISPR-Cas12a-based electrochemical aptamer sensor was developed for ultra-high-SN detection of the cancer biomarker MUC1 [765]. The synergistic effects of aptamer dual recognition, Cas12a-mediated trans-cleavage, and glucose oxidase (GOD) enzyme-catalyzed signal amplification significantly enhanced selectivity and SN. The detection principle involves measuring the oxidation current change from H_2_O_2_ generated by the glucose oxidation reaction. This occurs when Cas12a activity cleaves probe single-stranded DNA (pDNA) within the GOD-pDNA/magnetic Fe3O4@Au (MGNP) complex, releasing GOD. The sensor exhibited excellent linearity in the range of 1.0 × 10^−17^–1.0 × 10^−10^ g/mL, with an extremely low LoD of 7.01 × 10^−18^ g/mL. Its applicability was also confirmed in real medical samples [765].

Nano-porous interdigital electrodes (NP-IDE) employing single-walled carbon nanotubes as transducers exhibited exceptional selectivity in detecting DNA (with fM SN) and BC biomarker proteins (with pg/L SN for p53) [766].

3D Microelectrode Arrays (3D MAs) provide superior detection characteristics (response time and detection levels) compared to conventional planar macroelectrodes [761]. 3D MAs demonstrated three-fold higher SN than ELISA (the optical-based gold standard) [767] and proved capable of detecting RNA down to picomolar concentrations [761]. Particularly in microfluidic environments, 3D MAs demonstrated high SN even with minimal samples of 0.3 µL, compared to ELISA’s 50 µL sample size [767]. The 3D morphology controls electrode tip discharge, enhances the diffusion profile of target species, and utilizes current concentration phenomena to reduce the active area of individual electrodes [761].

A portable disposable solid-contact ion-selective potentiometric sensor (SC-ISE) has been developed for rapid measurement of sarcosine, a PCa biomarker, with detection limits as low as 9.95 × 10^−13^ M [768]. Furthermore, it exhibited a Nernstian response in the 10^−7^–10^−12^ M range and successfully detected sarcosine in urine samples without pretreatment, demonstrating its clinical applicability as a point-of-care sensor meeting WHO ASSURED criteria [768].

A clinically applicable electrochemical micro-nano motor biosensor employing a compact MD for the multiplex detection and assessment of BC biomarkers has been developed [769]. The MOF-based micro-nano motor (MOFtor) biosensor simultaneously detected and quantified BC biomarkers, such as ER, PR, HER2, and Ki67 with elevated SN. MOFtor functionalized with aptamers and antibodies on SiO_2_@Co-Fe-MOF operated as a miniature swimmer under an electric field to capture targets, facilitating an automated sample-amplification-output system that enhances and transmits four electrochemical signals [769]. The biosensor achieved detection limits of HER2 0.01420 pg/mL, ER 0.03201 pg/mL, Ki67 0.01430 pg/mL, and PR 0.01229 pg/mL. Compared to conventional biopsy (approximately 1 week), this biosensor demonstrated a short detection time of 40 min, indicating significant potential as a rapid and precise auxiliary diagnostic tool in clinical settings [769].

#### 7.3.3. Application of Electrochemical Biosensors for Age-Related/Chronic Diseases Diagnosis in POCT

An electrochemical platform integrating aptamers, magnetic NPs, and the CRISPR/Cas12a system has been developed for the rapid and precise detection of cTnI, a myocardial injury marker [770]. The principle involves complementary DNA (probe 2, P2) released upon aptamer–cTnI binding triggering Cas12a trans-cleavage, which reduces the electrode signal. This achieved an LoD of 10 pg/mL and a linear range of 100–50,000 pg/mL. This sensor demonstrated excellent selectivity and SN even in serum samples, suggesting its utility as a tool for CVD diagnosis, prognosis assessment, and treatment monitoring [770].

Detecting intracellular action potentials (Aps) is essential for a comprehensive understanding of cardiac pathogenesis and effective drug screening [771]. Microelectrode array (MEA) equipped with microelectrodes of various sizes, combined with microelectroporation technology, successfully recorded APs within cardiomyocytes [771]. Simulations and experiments demonstrated that large electrodes can also be utilized for intracellular AP acquisition. Large electrodes exhibited high amplitude and SNR, while small electrodes showed high perforation efficiency and single-cell signal yield [771]. Consequently, small electrodes were found suitable for densely packed cells, and large electrodes for dispersed cells. This confirms the suitability of each electrode type for different cell arrangements, presenting new possibilities for developing low-cost, high-quality intracellular AP recording electrodes [771].

The first glucose meters of the 1970s lacked precision. However, by 1980, the glucose meter enhanced functionality with a digital display. Inexpensive, low-blood-volume meters in the 1980s established self-monitoring of blood glucose as the standard [772]. The integration of A1C testing that measures the average amount of sugar in individual blood over the past three months and insulin pumps facilitated the Diabetes Control and Complications Trial, which validated the advantages of glucose regulation [773]. The proliferation of continuous glucose monitoring (CGM) devices has driven advancements in non-invasive and minimally invasive blood glucose measurement technologies. These offer the advantage of reducing pain, discomfort, and infection risks compared to fingerstick blood sampling [774]. Non-invasive methods measure glucose levels via skin-adhered sensors [775], while minimally invasive methods utilize bodily fluids like tears [776,777].

Remote-controlled smart contact lenses (SLCs) capable of simultaneously performing non-invasive glucose monitoring in tears and treating diabetes have been developed [729,730]. Keum et al. developed a wearable contact lens comprising a real-time electrochemical biosensor, an on-demand flexible drug delivery system (f-DDS), a resonant inductive wireless energy transfer system, a complementary integrated circuit (IC)-based microcontroller chip with a power management unit (PMU), and a remote radio frequency (RF) communication system [729]. It identifies glucose levels in tears instantaneously, eliminating the necessity for invasive blood tests. The GOD-based biosensor exhibited therapeutic effects comparable to Avastin injections by administering drugs on demand, demonstrating potential for advancement as a next-generation wearable therapeutic and diagnostic platform for the management of ophthalmic diseases and chronic conditions.

Tears show promise as an alternative means for diagnosing diabetes in blood, and SLCs are being utilized to measure glucose in tears. However, their clinical application remains limited due to controversy surrounding their correlation with blood glucose (BG) levels [730]. To resolve this controversy, a study was conducted utilizing wireless, flexible SCLs to examine the relationship between tear glucose (TG) and BG [730]. SCLs can continuously monitor basal TG at intervals shorter than one minute while eliminating the effects of reflex tears, thereby introducing the notion of “individual delay time,” which signifies variations in metabolic rates among individuals. Consequently, exact correlations between TG and BG were established among various subjects, including healthy individuals, diabetic patients, rabbits, and beagles. This study demonstrated an improved accuracy of TG-based BG prediction and the promise for the clinical utility of non-invasive diabetes monitoring [730].

Sweat, a biological fluid, is also emerging as a reliable surrogate indicator for blood analyte concentrations [733]. A wearable/disposable sweat-based glucose monitoring device integrated with a feedback-type transdermal drug delivery module has been developed [778]. The device integrates humidity, glucose, pH, and temperature sensors to detect changes in sweat composition in real time, while achieving high electrochemical SN and stability through a porous gold electrode and a fixed GOD-based structure. A combination of a stretchable heater and metformin-loaded phase-change NPs within hyaluronic acid hydrogel microneedles enabled stepwise and precise drug release according to temperature patterns. This multilayer patch system enhanced portability and accuracy through sensor calibration and a minimalist layout, representing a significant closed-loop therapeutic platform for non-invasive, personalized blood glucose management [778]. In a similar study, a bio-based cellulose paper glucose monitoring sensor utilizing methylated and phosphorylated microfibrillated cellulose (MFC) improved sweat capillary flow and enabled accurate glucose detection with just 1 µL of sweat [779]. Methylation provided chemical resistance, while the multilayer micro-patch design optimized sweat collection and detection efficiency. Integrated with an artificial transdermal drug delivery device (agarose gel), this system demonstrated stable performance under varying sweat volume, temperature, and pH conditions. Furthermore, temperature-dependent metformin release enabled a reliable closed-loop switch system for diabetes management [779].

### 7.4. Optical Biosensors

#### 7.4.1. Concept and Features of Optical Biosensors

Optical biosensors are devices that generate and detect signals by utilizing the interaction between light and biomarkers. They include diverse technologies such as SERS, fluorescence, absorption/colorimetry, and SPR. Optical biosensors offer sensitive, economical, and broadly applicable diagnostic techniques, frequently integrated with smartphones for result delivery via picture analysis [780]. Nanoparticles are used for signal amplification in SERS, fluorescence, and colorimetric-based assays.

#### 7.4.2. Application of Optical Biosensors for Cancer Diagnosis in POCT

The development of the DHOR portable fluorescence detection device demonstrated a significant advancement for rapid and cost-effective analysis of D-2-hydroxyglutaric acid (D-2-HG), a key biomarker for gliomas. acute myeloid leukemia, and chondrosarcoma [781]. Accumulation of D-2-HG due to mutant isocitrate dehydrogenase (IDH) activity competitively inhibits various α-ketoglutarate-dependent dioxygenase activities, thereby promoting tumorigenesis and carcinogenesis through epigenetic changes [782]. Unlike conventional methods such as liquid chromatography–tandem mass spectrometry (LC-MS/MS) and MRS, which are time-consuming and unsuitable for POC use, DHOR enables accurate D-2-HG detection from serum, urine, and brain tissues within approximately 5 min. Its low-cost design using off-the-shelf components allows rapid intraoperative assessment of IDH mutation status in tumor tissues, potentially improving real-time surgical decision-making and patient outcomes [781].

The smartphone-based optical microscope platform (EpiView-D4) directly quantified protein biomarkers in cell lysates using a polymer brush-based immunodiagnostic chip (D4) and simultaneously evaluated cell morphology and HER2 protein expression in fine needle aspiration (FNA) samples for BC diagnosis [736]. Pilot studies also accurately classified HER2-positive/negative tumors, demonstrating that this device can serve as a low-cost, rapid, and accurate alternative for BC diagnosis in settings with limited access to pathology services [736]. The buoyancy-lifted bio-interference-orthogonal organogel messenger (BLOOM) POC device achieved a SN of 88.8 percent and a SP of 88.9 percent for bladder cancer diagnosis, surpassing the SN of 20 percent of existing commercial kits [783]. It was also able to detect early-stage bladder cancer. The device measures fluorescence changes via smartphone without interference from patient hematuria [783]. An OLED-based active plasmon colorimeter biosensor detected LC biomarker neuron-specific enolase (NSE) in the 1–100 ng/mL range, demonstrating a low LoD of 200 pg/mL and high selectivity, showing potential for LC diagnosis [784].

SPR biosensor based on molecular aptamer beacon (MAB) conversion and tyramine signal amplification (TSA) was developed for the highly sensitive and specific detection of HER2-positive exosomes [785]. Following exosome capture, exposed G4-hemin induced tyramine-coated AuNPs deposition, amplifying the SPR signal. The use of non-enzymatic G4-hemin overcame enzyme limitations. Furthermore, high SP was achieved through dual recognition of protein and lipid membranes in exosomes. The sensor operated within the range of 1.0 × 10^4^ to 1.0 × 10^7^ particles/mL and accurately distinguishes patients from healthy individuals, demonstrating its clinical potential for HER2-positive BC diagnosis [785].

A SERS/electrochemical dual-mode biosensor was developed for the highly sensitive on-site detection of miR-106a, a marker associated with gastric cancer [786]. Based on a Ag nanorod array electrode coated with multi-functionalized MoS_2_ nanosheets, a sandwich-structured sensor was realized that simultaneously ensures stability and SN with a capability of distinguishing single-base mismatches, achieving low detection limits of 67.44 fM in SERS mode and 248.01 fM in electrochemical mode. Its excellent performance in human serum further enhances its applicability for early cancer diagnosis [786]. SERS has traditionally struggled to distinguish subtle spectral differences in complex matrices due to peak overlap, baseline noise, and intermolecular interactions. Integrating SERS with sophisticated AI data analysis effectively mitigates these challenges, enhancing analytical precision [787]. This method facilitates accurate differentiation of Raman spectra between healthy individuals and patients, significantly enhancing the potential of liquid biopsy in clinical applications [788,789].

The DNA-controlled protein fluorescence system represented a significant advance in biosensing technology due to its ability to accurately monitor intracellular ATP [790]. The system differentiated between cell types based on intracellular ATP levels, as seen in the significantly higher ATP levels in A549 LC cells compared to normal BEAS-2B cells, indicating its potential for developing cancer diagnostics [790].

A novel multi–ultra-sensitive coffee ring biosensor leveraging asymmetric nanoplasmonic structures has been recently developed [726]. Biomarkers were concentrated via droplet evaporation on a nanofiber membrane, and a second plasmonic droplet containing functionalized gold nanoshells was superimposed and dried to form an asymmetric nanoplasmonic pattern [726]. The device achieves protein detection down to 3 pg/mL within 12 min through a two-step droplet-based concentration mechanism: biomarker preconcentration via evaporation on a nanofiber membrane, followed by the deposition and drying of a secondary plasmonic droplet containing functionalized gold nanoshells. This process generates an asymmetric nanoplasmonic pattern that amplifies local electromagnetic fields, dramatically enhancing optical signal intensity [726].

#### 7.4.3. Application of Optical Biosensors for Age-Related/Chronic Diseases Diagnosis in POCT

A smartphone-based POCT device utilizing fluorescence technology to detect cholesterol, glucose, lactate, and uric acid has been introduced for rapid monitoring of metabolic biomarkers [791]. Using an ANN-based smartphone app to analyze fluorescence RGB·HSV, it detected targets in serum within 50 min, demonstrating R-value of 95% or higher and performance comparable to standard methods. It also showed excellent normal/abnormal discrimination, indicating its potential as a portable, low-cost POCT device for rapid monitoring of metabolic biomarkers [791].

A sensor utilizing a plasmonic etalon nanostructure for TG detection in a SCL has been proposed. The proposed structure generates light interference and surface plasmon resonance between two metallic reflective surfaces, amplifying the electric field and consequently attaining heightened SN to variations in refractive index [792]. Constructive interference occurs when 2nd=mλ, linking light wavelength (λ), resonance order (m), layer distance (d), and refractive index (n). That is, if the interlayer distance or refractive index changes due to glucose binding to the etalon surface, the resonant wavelength (color) changes. The elevated quality factor (Q-factor) further amplifies detection efficacy. The engineered lens was capable of detecting glucose in PBS solution within the concentration range of 0.15–10 mM, exhibiting significant SN even at minimal levels [792].

Aptamer-based biosensors are emerging as promising tools for the early diagnosis of aging and age-related diseases due to their high SN and stability [793]. Optical aptasensors detect target molecules through changes in optical signals, including fluorescence, colorimetry, and biolayer interferometry (BLI) [793]. An aptasensor based on nitrogen-doped carbon dots (NCD) was developed for the detection of tau protein, a crucial biomarker for AD. The NCD fluorescence is suppressed by the aptamer; however, upon binding with tau protein, the aptamer is liberated, reinstating fluorescence and facilitating signal detection with an LoD of 3.64 ng/mL [794]. The aptasensor for detecting amyloid-β1-40 oligomer (Aβ40-O), a key biomarker for early diagnosis of AD, is a label-free colorimetric biosensor composed of an aptamer-AuNP conjugate. It relies on visible color changes arising from interactions among aptamers, the target molecules, and nanomaterials such as AuNPs [795]. This aptasensor demonstrated high SN with an LoD of 3.03 nM and a linear detection range from 10.00 nM to 100.0 nM [795]. Another dual-amplification colorimetric sensor for Aβo detection achieved an LoD of 0.23 pM [796]. A biosensing platform for GDF15 detection was developed, featuring automated, high-throughput, and real-time online monitoring capabilities using BLI- Enzyme-Linked Aptamer Sorbent Assay (ELASA) technology [797]. This platform detected growth differentiation factor-15 (GDF15), a biomarker for glaucoma and aging, with an exceptionally low LoD of 5–6 pg/mL [797].

### 7.5. Nucleic Acid Amplification Tests (NAATs)

#### 7.5.1. Concept and Features of NAATs

NAATs are used to identify pathogens or disease biomarkers by extracting, amplifying, and detecting specific nucleic acid (DNA or RNA) sequences. PCR, LAMP and nanotechnology have been used.

#### 7.5.2. Application of NAATs for Cancer Diagnosis in POCT 

Loop-mediated isothermal amplification (LAMP) is a nucleic acid amplification technology that is faster than traditional PCR for nucleic acid amplification because it operates at a single, constant temperature, eliminating the need for a thermal cycler [798]. When combined with equipment-free sample preparation techniques such as ColdSHOT, LAMP facilitates highly sensitive and rapid on-site diagnostics in portable, POC settings [799]. An extraction-free HPV DNA test using LAMP-based detection of HPV DNA has been developed [800]. This test uses a low-cost benchtop heater/fluorescence reader to provide results within one hour, holding potential to expand cervical cancer screening in resource-limited settings [800].

ctDNA levels are present at extremely low concentrations in early stage of cancer. To address this issue, various amplification methods for detecting ctDNA have been developed. Surface-Limited Amplification (nSLAM) is a technology that directly amplifies and accumulates target ctDNA on the surfaces of Fe_3_O_4_-Au core–shell NPs (CSNPs) [801]. CSNPs exhibit dispersibility and superparamagnetism, acting as nanoelectrodes in PCR. Following magnetic collection, signals are reamplified via electrochemical measurement. This enabled ultra-high SN detection at approximately 3 aM levels and rapid detection within 7 min metastatic BC ctDNA detection in vitro [801].

The hybridization chain reaction (HCR) is an efficient, enzyme-free method for DNA amplification that runs at ambient temperature [802]. Two stable DNA hairpin species coexist in solution until the initiator strand triggers a series of hybridization events. This technology, combined with electrochemical DNA-based sensors, enables the measurement of nucleic acids in whole blood without calibration [802]. This approach enabled the quantitative detection of miR-21 with a SN of 1 pM and a dynamic range from 1 pM to 1 nM [803].

A dual-recognition-controlled electrochemical biosensor targeting CTCs has been developed [804]. In this sensor, two aptamer hairpin probes bind to adjacent proteins (EpCAM and MUC1) on the cell membrane, inducing a strand displacement reaction. This results in significant amplification of the electrochemical signal through dimer-like rolling circle amplification (RSA). Signal generation occurs only when the dual-expression protein is present, ensuring high analytical precision. When applying DNA nanostructure capture technology, the detection limit decreased to 3 cells/mL. Furthermore, stability and selectivity were demonstrated in whole blood matrices, indicating potential utility for cancer diagnosis and personalized treatment strategies [804].

DNA tetrahedron (DTN)-based logic signal amplification system has been developed [805]. This system utilized 3D DNA nanonetwork employing DTNs to perform catalytic hairpin assembly (CHA) reactions. It formed a hyperbranched 3D nanonetwork in the presence of target miRNAs (miR-21, miR-141), achieving a detection limit at the fM level, approximately 52 times lower than existing CHA systems [805]. This biosensor demonstrated the potential for distinguishing cancer cells from normal cells and identifying subtypes of BC cells based on modular logic gates [805].

#### 7.5.3. Application of NAATs for Age-Related/Chronic Diseases Diagnosis in POCT

Musunuru et al. demonstrated that CRISPR base editors delivered to living cynomolgus monkeys (Macaca fascicularis) via lipid NPs can efficiently and precisely modify disease-related genes [806]. Specifically, using CRISPR technology and lipid NPs, nearly complete hepatic suppression of the proprotein convertase subtilisin/kexin type 9 (PCSK9) gene—a factor increasing cardiovascular disease risk—was observed. Concurrently, blood PCSK9 levels and PCSK9-mediated low-density lipoprotein cholesterol levels decreased by approximately 90% and 60%, respectively. This effect was maintained for 8 months after a single treatment [806].

A highly sensitive chemiluminescent immunoassay was developed, which employed a sandwich architecture comprising antibodies immobilized on magnetic beads and ssDNA detection antibodies to initiate RCA reactions and amplify signals [807]. Aβ42 and Aβ40 demonstrated minimal detection thresholds of 1.99 pg/mL and 3.14 pg/mL, respectively. The Aβ42/Aβ40 ratio distinguished AD patients from healthy controls with an SN of 90.48% and a SP of 63.64%, demonstrating significant potential for non-invasive diagnosis and assessment of AD [807].

### 7.6. FDA-Approved or Cleared POC Devices

In 2016, the cobas EGFR Mutation Test v2 became the first FDA-approved liquid biopsy test to detect specific EGFR gene mutations in cfDNA from plasma samples. This test is used as a companion diagnostic to determine which patients with metastatic NSCLC are eligible for first-line, maintenance, or second-line treatment with erlotinib, an EGFR-targeted medication [808].

Some MDs are used to isolate and analyze liquid biopsy markers such as CTCs and exosomes. The ClearCell^®^ FX system (Biolidics, Singapore) is an automated, label-free device that enriches intact and viable CTCs from whole blood [809]. The system demonstrates over 80% SN and SP in detecting CTCs from clinical samples and has achieved multiple regulatory approvals, including CE-IVD, US FDA Class I, and CFDA Class I registrations [809].

In January 2019, the FDA approved the first POC total PSA test, the Silver Amplified NeoGold ImmunoAssay (Sangia), which gives results in under 15 min from a fingerstick blood sample [810]. With a digital rectal examination (DRE), it detects PC in men 50 and older, streamlining testing and patient care with rapid results outside of a lab [810].

### 7.7. Deployment Considerations of POC Versus Laboratory-Based Biosensing Platforms

The deployment of POC biosensing platforms presents a distinct paradigm from conventional laboratory-based systems in terms of analytical SN, robustness under ambient environments, and user interface design, particularly in the context of elderly populations requiring continuous or longitudinal health monitoring [811].

#### 7.7.1. Analytical Sensitivity

From a SN standpoint, POC devices have made substantial advances through the integration of microfluidics, plasmonics, and nanomaterial-enhanced sensing layers, achieving detection limits that rival or surpass centralized assays such as ELISA or chemiluminescent immunoassays [504,726,811,812,813]. These innovations enable the quantification of ultra-low-abundance biomarkers, including circulating DNA, proteins or peptides at femtomolar to attomolar levels, critical for early disease detection in AD or cancer screening [801,814,815]. In contrast, laboratory-based platforms, though traditionally regarded as the gold standard for analytical precision, often rely on large-volume samples, batch processing, and controlled reaction conditions, which may limit their ability to capture transient or low-copy biological signals relevant in early pathophysiological changes [738,816].

#### 7.7.2. Robustness

In terms of robustness, laboratory systems benefit from highly standardized protocols and controlled environmental conditions [717]. While highly reliable when conditions are met, the entire process is susceptible to delays from sample transport and errors associated with pre-analytical, analytical, and post-analytical phases [817]. POC devices, which must function reliably in diverse, non-clinical environments, prioritize robustness. To address this, modern POC biosensors emphasize mechanical and environmental stability as core design principles aligned with the WHO’s ASSURED (Affordable, Sensitive, Specific, User-friendly, Rapid, Equipment-free, and Deliverable) framework [818]. Challenges remain, however, as environmental fluctuations, sample matrix heterogeneity, and user-dependent variability can affect signal reproducibility [817]. POC systems incorporate solutions to mitigate these challenges. The integration of advanced sensors and microfluidic components helps in precise fluid control and reduces the system’s susceptibility to contamination [717]. Some optical sensing platforms are designed to be intrinsically stable, making them largely immune to positional changes and mechanical vibration, often allowing operation on a lab bench rather than a stabilized optical table [753]. Furthermore, the complexity and noisy nature of POC data can be effectively managed by integrating AI and ML algorithms, which are capable of learning complex patterns and improving diagnostic accuracy and reliability despite the imperfections of the sensors [717]. The BLOOM assay, for instance, enhances robustness against complex sample heterogeneity (like blood in urine) by using a biphasic system that spatially separates the biomarker recognition from the signal transduction [783].

#### 7.7.3. Usability

The usability and interface design of POC platforms are central to their success, particularly for elderly patients managing chronic diseases. Conventional laboratory diagnostics require skilled personnel, complex instrumentation, and repeated patient visits, which can pose accessibility barriers for older adults with limited mobility or cognitive decline [813,819]. In contrast, POC platforms prioritize user-friendliness and accessibility, making them uniquely suitable for decentralized testing and self-monitoring, which is critical for the aging population [809]. POC solutions enable patients to potentially assess their health conditions without the need for regular physician visits [717]. They are designed to be operated feasibly by patients or in a “near-patient” format, generally requiring no specialized training [717]. Devices like the glucometer exemplify this success, dominating diabetes monitoring due to their affordability and ease of use for patients [820]. POC devices are portable and often leverage inexpensive and simple formats, such as paper-based analytical devices [813] or lateral flow assays [750]. Their simplicity often extends to sample preparation, employing non-invasive or minimally invasive sampling methods. Integration with ubiquitous smart technologies, like smartphones, facilitates reading and interpretation of results [755,777]. Digital tools offer cost savings and can shorten the administration time of certain tests, making the patient journey more patient-friendly. AI integration into POC automates complex result interpretation (e.g., classifying faint test lines) and data analysis, which reduces the dependency on untrained users and enhances diagnostic accuracy, empowering frontline health workers and potentially individual patients [719]. This is especially beneficial for ensuring equitable access and reliable delivery to diverse and low-literacy populations [821].

## 8. From Discovery to Clinical Application

From the discovery of a specific protein in the urine samples of patients with myeloma by Bence Jones in 1846 to today’s AI-enabled liquid biopsy systems, the trajectory of biomarker research reflects a shift from isolated biochemical observations to integrated, regulatory-validated clinical frameworks [822].

### 8.1. Historical Development of Biomarkers

From the 1960s to the 1980s, molecular identification transformed cancer diagnostics with biomarkers like AFP (HCC), CEA (CRC), and PSA (PCa) [823]. In the 1990s–2000s, genomic and proteomic tools advanced biomarker discovery. BRCA1/BRCA2 enabled hereditary cancer screening. CA 19-9 (PC) and microRNAs (CRC) revealed gene regulation and epigenetic mechanisms. Technologies like microarrays, MALDI-TOF, LC-MS/MS, and 2D PAGE supported high-throughput biomarker validation [822]. From the 2010s onward, clinical translation accelerated with FDA-approved markers: BTA and NMP22 (bladder cancer) [824], and CA-125/HE4 (ROMA test for ovarian cancer) [377]. Molecular diagnostics now routinely assess HER2, EGFR, KRAS, and ALK mutations to guide targeted therapies (e.g., trastuzumab, erlotinib, cetuximab) [372,825]. Liquid biopsy innovations have expanded their roles from early diagnosis to providing crucial information for patient prognosis and the real-time monitoring of treatment effectiveness and disease recurrence [395]. Integration of AI/ML-based multi-omics models began bridging laboratory discovery with real-time clinical decision support [826,827]. Proteomic clocks and AI-driven predictive models extend biomarker applications from cancer surveillance to aging-related disease prevention, representing a convergence of oncology, geroscience, and digital diagnostics [387].

### 8.2. Regulatory Frameworks

#### 8.2.1. United States (FDA)

In vitro diagnostic (IVD) biomarker tests are regulated as medical devices via 510(k), De Novo, or Premarket Approval (PMA) pathways, depending on risk class and predicate availability. The following table summarizes their major distinctions and clinical implications for biomarker-based diagnostic devices [828] (Table 13).

#### 8.2.2. Europe

The European Medicines Agency (EMA) operates a formal Biomarker Qualification process through Committee for Medicinal Products for Human Use (CHMP), providing qualification advice, letters of support, or a qualification opinion for specific contexts of use, facilitating consistent uptake across trials and submissions [831].

### 8.3. Regulatory, Validation, and Cost-Effectiveness for New Biomarkers

Introducing a new biomarker into routine clinical practice involves navigating complex regulatory requirements, ensuring robust multi-center validation, and addressing critical cost-effectiveness issues. These factors are crucial for successful adoption and widespread use [832].

#### 8.3.1. CLIA/LDT Considerations in the U.S.

In the United States, many early-stage biomarker tests are initially introduced as laboratory-developed tests (LDTs) under the CLIA, which are regulated by the Centers for Medicare and Medicaid Services to ensure analytical quality [833]. Although the FDA has intermittently sought to expand its oversight of LDTs as medical devices, a 2024 final rule was vacated by a federal court in March 2025 [834]. Subsequently, in September 2025, the FDA reverted to the pre-rule regulatory framework, while broader policy discussions continue [835]. As a result, developers are advised to pursue CLIA validation in the early stages and prepare for potential FDA submissions such as 510(k), De Novo, or PMA, when aiming to scale beyond a single laboratory or to secure national coverage and standardization.

#### 8.3.2. Multi-Center Validation Pathways and Evidentiary Standards

Translational readiness of biomarker-based diagnostics is significantly enhanced through prospective, multi-center studies that rigorously establish three key pillars: analytical validity across different sites and instruments, clinical validity against appropriate comparators, and clinical utility in terms of impact on clinical decision-making and patient outcomes [836]. To ensure transparency and reproducibility, study designs should adhere to established consensus standards for diagnostic accuracy, such as the Standards for Reporting of Diagnostic Accuracy Studies (STARD) guidelines [837]. These standards support the generation of robust estimates for SN, SP, AUC, LoD, and overall reproducibility, all within the framework of pre-specified analysis plans and external validation cohorts.

#### 8.3.3. Cost-Effectiveness and Implementation

From an implementation perspective, cost-effectiveness is a critical consideration for payers and health technology assessment (HTA) bodies. Evaluations typically incorporate quality-adjusted life years (QALYs), budget impact, downstream resource utilization (e.g., confirmatory imaging or biopsy), and diagnostic turnaround time [838]. Strategies to enhance value include enrichment approaches (such as applying age or risk thresholds and multi-analyte triage), reflex testing algorithms (initial screening followed by confirmatory diagnostics), optimizing site-of-care delivery (point-of-care versus centralized laboratories), and leveraging longitudinal monitoring to enable earlier detection and reduce overtreatment [839,840]. Ultimately, biomarker tests that demonstrate clear clinical actionability and a favorable economic profile in real-world, multi-center settings are more likely to gain payer coverage and widespread clinical adoption.

#### 8.3.4. FDA-Recognized Examples

Examples directly relevant to early detection include: (i) Epi proColon^®^ (methylated SEPT9)—the first FDA-approved blood-based CRC screening assay (PMA, 2016) [841]; (ii) Cologuard^®^ (multi-target stool DNA, mt-sDNA) for CRC screening (PMA, 2014) [842]; (iii) comprehensive genomic CDx panels such as FoundationOne CDx and Guardant360 CDx (PMA) for tumor profiling to guide therapy (not screening) [843]; and (iv) CellSearch^®^ for CTCs detection (FDA-cleared) for monitoring [428]. For aging-related conditions, the FDA has cleared the first blood test to aid in diagnosing AD—the Lumipulse G p-tau217/β-amyloid 1-42 plasma ratio (May 2025) and previously cleared a CSF β-amyloid 1-42/1-40 ratio test via De Novo (2022) [844]. At present, there is no FDA-approved MCED screening test. Several are under investigation, and many assays are available only as CLIA LDTs. Table 14 summarizes representative cancer and aging biomarkers that have obtained FDA or EMA approval, or are under regulatory review, illustrating the translation pipeline from discovery to clinical application.

## 9. Current Limitations and Future Directions for Early Diagnosis of Cancer and Aging

### 9.1. Current Limitations in Early Diagnosis of Cancer and Aging

#### 9.1.1. Biomarker-Related Limitations

The early detection of cancer and age-associated diseases can markedly enhance patient outcomes, decrease mortality, and provide prompt therapies. However, despite significant technological progress, several critical limitations currently hinder their clinical translation. One major challenge is the low abundance and biological heterogeneity of biomarkers. Early disease signals, such as ctDNA, CTCs, and EVs detected through liquid biopsy, are present at extremely low concentrations and exhibit high intra- and inter-tumor variability. The presence of tumor heterogeneity significantly increases the probability of false negatives and false positives. Similarly, aging-related markers such as p16, p21, and Lamin B1 show substantial inter-individual variation, and there is no clear distinction between normal and pathological aging states. This variability reduces SN and contributes to inconsistent results.

Another major barrier is the lack of established pre-analytical and analytical techniques impedes cross-study comparisons and clinical application. No commonly agreed cut-offs or reference values exist for aging-related biomarkers, specifically BAG indices obtained from neuroimaging, which complicates clinical interpretation. Moreover, confounding by age-related comorbidities such as chronic inflammation, immune dysfunction, and metabolic disorders can obscure true biomarker signals and increase the risk of false positives, especially in older populations.

#### 9.1.2. Technological and Analytical Limitations

Current techniques are constrained by technological and analytical restrictions. Many platforms confront an inherent trade-off between SN and SP, with ultra-sensitive detection increasing the possibility of false positives, which is especially challenging in population-wide screening.

AI and ML models have demonstrated transformative potential in cancer diagnosis, prognosis prediction, and biomarker discovery. However, despite their impressive analytical capabilities, several critical challenges must be addressed before these technologies can be fully integrated into clinical practice. The main barriers include data bias and representativeness, lack of external validation and generalizability, and limited interpretability of DL models, all of which undermine clinical reliability, regulatory acceptance, and physician trust. Table 15 summarizes the key technical and ethical challenges limiting the clinical adoption of AI/ML models and presents potential strategies to enhance reliability, transparency, and regulatory compliance.

In addition, existing early diagnostic tools, such as NGS and high-resolution imaging, require significant infrastructure, cost, and skill, limiting their use at POC settings. Crucially, most systems are still in the proof-of-concept or pilot-study stages, with no large-scale, multicenter prospective clinical validation.

Regarding POCT, despite these advantages, miniaturization that enables portability and low power consumption introduces inherent trade-offs. Reduced optical path lengths, limited reagent volumes, and microfabrication tolerances can amplify noise and inter-device variability [845]. Furthermore, miniaturized components are more sensitive to environmental perturbations such as temperature, humidity, or vibration [846]. Therefore, precise instrument calibration remains a critical challenge in POC deployment. Unlike laboratory analyzers that undergo regular professional calibration, POC systems must maintain long-term stability through self-calibration algorithms, embedded reference standards, or sensor redundancy [847]. Some platforms employ built-in calibration cartridges or cloud-linked quality control feedback loops to ensure consistent analytical performance across distributed devices [848].

**Table 15:** Limitations and Improvement Strategies of AI/ML Models for Clinical Translation.

Category	Key Limitations	Improved Strategy/Future Directions	Ref.
Dataset Bias and Representativeness	Many AI/ML models are trained on single-center or demographically narrow datasets, resulting in poor generalizability across populations. Imbalanced datasets (e.g., underrepresentation of minority groups, rare cancer subtypes) cause biased model predictions and reduced diagnostic fairness.	Implement fairness-aware training algorithms to ensure demographic balance. Employ FL to enable data diversity without centralizing sensitive data. Adopt model cards to disclose dataset composition, bias sources, and subgroup performance.	[849,850]
Lack of Cross-Cohort Validation and Overfitting	Many models perform well in internal validation but fail in external datasets due to overfitting. Lack of multi-institutional testing limits generalization. Medical data drift over time reduces algorithmic robustness.	Conduct multi-site, prospective validation and external benchmarking. Apply TL and domain adaptation to enhance robustness across cohorts. Establish continuous model monitoring and recalibration pipelines in clinical use.	[851,852]
Interpretability and Black-Box Barriers	DL models often act as “black boxes,” providing limited insight into decision-making. Lack of interpretability decreases clinician trust and impedes regulatory approval.	Develop XAI frameworks (e.g., SHAP, LIME, integrated gradients) to visualize key features influencing model outputs. Combine interpretable hybrid architectures (e.g., attention-based + explainability layers). Include clinician-in-the-loop validation for model reasoning.	[853]
FL and Distributed Model Deployment	Despite privacy advantages, FL faces challenges with data heterogeneity, communication overhead, and explanation consistency across institutions. Lack of harmonization hinders reproducibility.	Use FED-XAI frameworks integrating explainability with FL. Standardize communication protocols and ontology alignment among institutions. Implement federated evaluation benchmarks to harmonize model performance reporting.	[854]
Clinical Integration and Regulatory Adoption	Limited clinical trial evidence for efficacy and safety. Absence of standardized regulatory pathways and real-time post-market surveillance for AI tools.	Conduct prospective clinical trials aligned with FDA/EMA digital health guidelines. Establish AI-QMS for traceability and auditability. Encourage regulator–developer collaboration for explainability and patient safety.	[855]

Abbreviation: AI; artificial intelligence; ML; machine learning, DL; deep learning, FL; federated learning, TL; transfer learning, XAI; Explainable AI, SHAP; SHapley Additive exPlanations, LIME; Local Interpretable Model-Agnostic Explanations, FED-XAI; Federated Explainable Artificial Intelligence, QMS/AI-QMS; Quality Management System/Artificial Intelligence Quality Management System, FDA; U.S. Food and Drug Administration, EMA; European Medicines Agency.

### 9.2. Future Directions for Early Diagnosis of Cancer and Aging

We discuss prospective development directions based on significant instances of technology that showed notable improvements in early cancer and aging diagnosis compared to existing techniques (Table 16).

#### 9.2.1. Multi-Omics Integration and Personalized Biomarker Panels

The next generation of diagnostic systems will integrate genomics, epigenomics (including DNA methylation), transcriptomics, proteomics, metabolomics, and immune markers into comprehensive biomarker panels. This multi-omics approach will elucidate complex biological networks underlying cancer and aging, overcoming the sensitivity and specificity limitations of single-marker approaches. These integrated analyses will enable accurate early diagnosis and distinguish diverse aging pathways (“ageotypes”) within same-age cohorts, facilitating personalized risk prediction and intervention strategies.

#### 9.2.2. AI/ML Advancement

AI and ML algorithms will enable pattern recognition from large-scale, high-dimensional datasets, detecting early disease signals imperceptible to human analysis. Next-generation explainable AI (XAI), self-supervised learning (SSL), and foundation model methodologies will enhance diagnostic accuracy, reduce algorithmic bias, and increase clinician trust and adoption. When integrated into POCT platforms, these algorithms will enhance biomarker detection precision and reliability, particularly through advanced colorimetric and electrochemical sensing methods [719]. This is described in greater detail in Table 14.

#### 9.2.3. Ultra-Sensitive Detection Technologies

Next-generation high-sensitivity detection platforms are being developed to achieve picogram-level and single-cell resolution while maintaining minimal invasiveness. These systems integrate multiple complementary technologies to enhance diagnostic accuracy and responsiveness. Rapid screening can be accomplished through lateral flow assays and LAMP, while SERS enables molecular fingerprinting with exceptional specificity [719]. Electrochemical and plasmonics-based sensors provide real-time quantitative monitoring of dynamic biological processes, and FET sensors allow highly sensitive, label-free detection of biomolecular interactions [856]. Furthermore, nanomaterial-based sensors enable multiplexed, high-sensitivity analyses within miniaturized systems. The convergence of electrochemical, immunological, plasmonic, and aptamer-based sensing modalities represents a transformative step toward faster, more precise, and integrated diagnostic platforms.

#### 9.2.4. Miniaturized and Portable Diagnostic Platforms

Microfluidic lab-on-a-chip (LoC) systems are poised to revolutionize diagnostic testing by miniaturizing complex immunoassay workflows into highly integrated single-chip platforms. By combining microfluidic control, surface-enhanced nanostructures, and advanced photonic sensing modules, these systems enable the simultaneous detection of multiple biomarkers using only minimal sample volumes [857,858]. Such integration significantly improves assay speed, portability, and real-time quantification while reducing sample handling and reagent consumption. Moreover, their compact and automated design enhances accessibility in resource-limited settings. Emerging sensor architectures, including dual-core D-shaped photonic crystal fiber-based SPR sensors [859] and dielectric-grating near-field sensors [860], offer exceptional chemical stability, high sensitivity, and robust multiplexed performance, making them ideally suited for portable POC applications.

#### 9.2.5. Organ-on-Chip Systems for Dynamic Disease Modeling

Organ-on-a chip (OoC) platforms will enable tissue-specific modeling of disease microenvironments for longitudinal protein monitoring [861,862]. These microphysiological systems utilize living human cells within microfluidic environments to replicate organ-level physiology and pathology, offering ethical and predictive alternatives to animal models [863]. Cancer-on-a-chip models incorporating patient-derived cancer cells and stromal components will track secretion patterns of VEGF and cytokines throughout drug response cycles, bridging in vitro discovery with in vivo validation [864,865,866]. These platforms will recreate TMEs and organ interactions, facilitating personalized drug testing and metastasis modeling. Supported by regulatory frameworks such as the 2022 FDA Modernization Act 2.0, this “clinical-trial-on-a-chip” approach will advance precision oncology, drug safety evaluation, and translational cancer research [867].

#### 9.2.6. Longitudinal Monitoring and Digital Integration

Future biomarker research will prioritize longitudinal tracking [868,869] and multiplexed biosensing [870] for dynamic monitoring of biomarker fluctuations, enabling early relapse detection and therapy response assessment. These systems will permit continuous surveillance of aging-related proteins under controlled flow and stress conditions, providing mechanistic insights into proteomic aging clocks and therapy-response trajectories. Integration of AI-driven analytics with cloud-linked longitudinal databases will enable trend analysis, anomaly detection, and predictive modeling of biomarker evolution across time and organ systems [871]. This digital-biosensing synergy will transform episodic diagnostics into continuous, personalized disease monitoring, particularly for cancer and age-associated chronic diseases.

#### 9.2.7. Manufacturing and Quality Assurance

Nanotechnology will redefine POCT device fabrication through nanometrology techniques such as atomic force microscopy (AFM) and scanning tunneling microscopy (STM), ensuring device integrity and performance at the nanoscale. These quality assurance approaches will be essential for ultra-miniature devices, including those deployed in Internet of Things (IoT) healthcare applications. Regulatory harmonization will ensure consistent standards and safe deployment across healthcare settings [872].

#### 9.2.8. Paradigm Shift Toward Preventive Medicine

A fundamental transition from single-point testing to longitudinal and preventive screening will identify pre-disease states and enable early interventions. This includes tracking early deviations in brain aging trajectories BAG measures and monitoring circulating biomarkers for cancer recurrence risk. 

#### 9.2.9. Clinical Translation Framework

Achieving clinical translation will require robust regulatory and standardization frameworks, including clearly defined performance criteria, quality control systems, and privacy safeguards. Successful implementation will depend on collaborative efforts across industry, healthcare institutions, and academic research centers to establish unified standards for these emerging technologies [317].

## 10. Conclusions

Cancer and aging are distinct but interconnected biological processes that share common mechanisms such as genomic instability, telomere dysfunction, cellular senescence, chronic inflammation, and metabolic abnormalities. This overlap offers opportunities for biomarker discovery enabling early detection and targeted interventions. Early diagnosis is critical for improving treatment outcomes, reducing mortality, and enabling timely therapeutic interventions. However, conventional diagnostic methods often rely on invasive biopsies, imaging tests with low SN, and single-protein analyses, potentially missing early signals and delaying intervention.

Emerging biomarker-based approaches are shifting this paradigm. Liquid biopsy enables non-invasive detection of ctDNA, cfDNA, CTCs, EVs, and miRNA in blood or other biofluids. However, its clinical performance is limited by low analyte concentrations and high biological variability. Advanced analytical and detection platforms, including NGS, ddPCR, LAMP, MDs, SERS sensors, FET biosensors, and electrochemical sensors, along with nanotechnology further enhance SN with minimal samples. Concurrently, AI and ML/DL enable the identification of subtle, high-dimensional patterns, improving diagnostic accuracy and risk evaluation.

Furthermore, wearable biosensors and miniature lab-on-a-chip systems provide real-time POCT. Emerging biomarkers, such as biological age and BAG, provide new means to identify at-risk populations before symptom onset. The convergence of liquid biopsy, multi-omics, AI, and smart biosensors promises a shift from single-point diagnostics to continuous personalized monitoring.

To apply these advances to routine clinical practice, additional efforts are required to establish standardized protocols, conduct large-scale clinical validation, and develop accessible POC diagnostic platforms. These innovations hold the potential to revolutionize the early detection and personalized management of cancer and age-related diseases.

## Figures and Tables

**Table 1:** Seven Genetically Supported Biomarker Genes for Brain Aging [258].

Gene	Main Function	Associated with BAG	Key Notes and Disease Links
MAPT	Encodes Tau protein; stabilizes neuronal microtubules	Negative causal association (blood and brain tissues)	Linked to AD and Parkinson’s disease; high expression correlates with higher glucose, blood pressure, and ApoA
TNFSF12	Encodes TWEAK cytokine; modulates inflammation	Negative causal association (transcript and protein level)	Regulates blood–brain barrier inflammation and supports glucose homeostasis; strong multi-layer genetic evidence
GZMB	Encodes Granzyme B; serine protease	Negative causal association	Mediates inflammation (induces IL-8, MIP2); linked to vascular and skin aging
SIRPB1	Immune receptor (Ig superfamily)	Negative causal association	Associated with longevity genetics; linked to insomnia, IL-1α, glucose
GNLY	Encodes Granulysin from NK/T cells	Positive causal association	Antimicrobial and pro-inflammatory; induces cytokines (CCL5, IL-10, IL-6, IFN-α)
NMB	Neuropeptide	Positive causal association	Regulates feeding behavior and obesity—key factors in aging
C1RL	Complement-related protease	Positive causal association (brain tissue)	Linked to AD, obsessive–compulsive disorder, anorexia nervosa; regulates immune/inflammatory pathways

**Table 3:** Comparison of DNAm clocks.

Generation	Clock	CpG Sites	Key Features	Performance	Ref.
1	Hannum	71	A single-tissue clock developed primarily based on white blood cell (blood) data	While it shows high accuracy in chronological age prediction, applying it to non-blood tissues or children may result in biased age estimates.	[304]
	Horvath	353	A pan-tissue prediction model developed using over 8000 samples from 51 healthy tissue and cell types. Applicable across the entire lifespan (from fetal samples to individuals aged 100).	Shows a high correlation with chronological age of r > 0.90 across the entire age range sample.	[305]
	Weidner	3	Blood aging can be tracked using just three CpG site (*ITGA2B, ASPA and PDE4C*) changes.	Higher precision than age predictions based on telomere length, cost-effective, and practical forensic applicability. Variation in age predictions correlates moderately with clinical and lifestyle parameters	[306]
2	PhenoAge	513	Designed to predict ‘Phenotypic Age’ by combining 9 key clinical biomarkers with chronological age.	Outperforms 1st-generation clocks in predicting mortality, healthy life expectancy, cardiovascular disease, and multiple comorbidities.	[308]
	GrimAge	1030	Developed to predict time to death by integrating seven plasma protein surrogate biomarkers and a DNA methylation-based surrogate marker for smoking history (pack-years).	It demonstrates the strongest predictive power for lifespan and healthspan. AgeAccelGrim excels in predicting mortality, coronary heart disease, and cancer.	[309,310,311,312]
	DunedinPACE	173	Developed to measure the pace of aging by analyzing 20 years of longitudinal data. It separates aging from cohort effects and survival bias using single-age birth cohort data.	Provides additional predictive power over existing clocks including GrimAge, associated with predicting morbidity, disability, and mortality. Shows a high correlation with aging rate (r = 0.78).	[313]
	CausAge, AdaptAge, and DamAge	586, 1000 and 1090	Developed by integrating causal information through epigenome-wide Mendelian randomization. DamAge tracks harmful methylation changes, while AdaptAge tracks beneficial adaptive changes.	DamAge correlates with negative outcomes such as mortality, while AdaptAge is associated with beneficial adaptations. It demonstrates SN to short-term interventions and may serve as a preferred biomarker for tracking events that influence aging-related traits during development.	[314]
	IntrinClock	381	A clock designed to remain unaffected by changes in immune cell composition	High overall prediction accuracy across a variety of tissues and SN to cellular interventions	[315]
	IC Clock	91	Trained to predict IC based on clinical assessments (cognition, motor function, psychological well-being, sensory abilities, vitality)	Emerges as one of the most powerful predictors of mortality, surpassing the 1st and 2nd generation clocks (HR = 1.38)	[316]
	Aging Atlas	>900,000	the atlas integrates differentially methylated positions, variably methylated positions, and entropy to capture both deterministic and stochastic changes	Shifts the focus from age-prediction clocks to a systems-level atlas that identifies mechanisms, biomarkers, and intervention targets across human tissues	[317]

**Table 5:** FDA-approved HER2 assessment methods [372].

Method	Assay/Test	FDA Approved Year	Notes/Indications
IHC	HercepTest	1998	First FDA-approved companion diagnostic with trastuzumab; used rabbit polyclonal antibody with signal amplification
	PATHWAY 4B5	2016	Rabbit monoclonal antibody assay; approved for HER2-low breast cancers; expansion to HER2-ultralow expected by 2025
FISH	INFORM HER2/neu	1997	First FDA-approved prognostic HER2 test.
	PathVysion	2001	Companion diagnostic for trastuzumab therapy.
	PharmDx Kit	2005	For ERBB2 (HER2) amplification testing.
	HER2 FISH	2013	Approved alongside trastuzumab emtansine (T-DM1).
CISH	SPOT-LIGHT	2008	First FDA-approved CISH assay for ERBB2; more followed later.
QIA algorithms	PATHIAM, ScanScope XT, VIAS, ARIOL, ACIS	Various (last approved ~2010)	Automated HER2 IHC image analysis; no new approvals in past 15 years; predated HER2-low focus.
Standardized Quantitative Controls	Microbead calibrator slides	Various (post-2000s)	Cell-sized microbeads with synthetic antigens; improve reproducibility and LoD in IHC

**Table 7:** Representative Protein Biomarkers for Cancer and Aging.

Biomarkers	Cancer or Condition	Biological/Clinical Role	Sample Type	Detection/Analytical Platform	Clinical or Regulatory Status	Evidence Level/Stage
PSA	PCa	Serine protease secreted by prostate epithelium; elevated in malignancy and benign disease	Blood/ Serum	Immunoassay, ELISA, chemiluminescence	Widely used; PHI test FDA-approved (2012)	Clinical practice, prospective validation [370]
fPSA/tPSA ratio	PCa	Composite index improving SP over total PSA	Blood	Immunoassay + mathematical model	FDA-approved (PHI = [−2]proPSA/fPSA × √PSA)	Clinical validation completed [371]
HER2 (*ERBB2*)	BC	Receptor tyrosine kinase predictive for trastuzumab response	Tissue	IHC, FISH, CISH, QIF, RT-PCR, RNA assay	FDA-approved companion diagnostic (HercepTest)	Extensive clinical validation [372]
ER/PR	BC	Hormone receptor markers guiding endocrine therapy	Tissue	IHC	FDA-approved assays	Routine clinical use [372]
CA 15-3 (MUC1)	BC	Glycoprotein shed from tumor cells; recurrence monitoring	Serum	ELISA, chemiluminescent assay	Research/clinical follow-up use	Retrospective and prospective cohorts [375]
CA-125 (MUC16)	OC	Tumor-associated glycoprotein; recurrence monitoring	Serum	Immunoassay, ELISA	Part of FDA-cleared multivariate tests (ROMA, OVA1)	Clinical and regulatory validation [377]
HE4 (WFDC2)	OC	Improves SP when combined with CA-125	Serum	Immunoassay	FDA-cleared (ROMA algorithm)	Clinical validation [379]
AFP/AFP-L3/DCP(PIVKA-II)	HCC	AFP oncofetal marker; AFP-L3 specific for HCC; DCP complements AFP	Serum	Immunoassay, lectin affinity assay	AFP-L3 FDA-cleared; DCP regionally approved	Clinical validation [380]
CEA/CA19-9	CRC, PC, GC	Circulating tumor markers for prognosis and recurrence monitoring	Serum	ELISA, electrochemical sensor	Clinical guideline recommended	Large cohort validation [381,382,384]
ProGRP/NSE/CYFRA 21-1	SCLC	Neuroendocrine markers correlating with therapy response	Serum	Immunoassay	Clinical application	Retrospective validation [383]
SCCA/HMGB1	Cervical cancer (HPV-related)	Tumor-associated and inflammatory proteins; correlate with HPV status	Serum/Tissue	ELISA, IHC	Research stage	Retrospective/exploratory [385]
PD-L1	Lung, melanoma, kidney	Checkpoint ligand guiding anti-PD-1/PD-L1 therapy	Tissue	IHC (22C3, SP142), RNA-seq	FDA-approved companion diagnostic	Clinical implementation [386]
p-Tau181/p-Tau217/NfL/GFAP	AD, dementia	Neural proteins indicating tauopathy and neurodegeneration	Plasma/CSF	SIMOA, immunoassay	CE-marked/validation ongoing	Longitudinal cohort validation [388]
GDF15/NT-proBNP/CRP/Leptin/IGF-1/sRAGE	Cardiovascular and metabolic aging	Proteins reflecting inflammation, metabolism, and CVD risk	Plasma/Serum	Multiplex proteomic assay	Research/prognostic validation	Large cohort studies [389]
ProtAge20 panel	Systemic aging	Proteomic aging clock predicting multimorbidity and mortality	Serum/Plasma	LC-MS/MS, computational modeling	Research use	Prospective cohort validation [387]

Abbreviations: PCa; prostate cancer, BC; breast cancer, OC; ovarian cancer, HCC; hepatocellular carcinoma, CRC; colorectal cancer, PC; pancreatic cancer, GC; gastric cancer, SCLC; small cell lung cancer, AD; Alzheimer’s disease.

**Table 8:** ProtAge20 Proteins by Tissue/Organ Source [387].

Tissue/Organ	Protein	Full Name	Major Biological Function	Aging Relevance
Central Nervous System	GFAP	Glial fibrillary acidic protein	Astrocyte structural protein	Marker of brain aging and neurodegeneration
	NEFL	Neurofilament light polypeptide	Axonal structure protein	Marker of neuronal damage
	PLXNB2	Plexin-B2	Axon guidance, cell signaling	Brain development and aging
	NCAMI	Neural cell adhesion molecule 1	Neuronal plasticity	Cognitive aging
	CLU	Clusterin (Apolipoprotein J)	Chaperone, amyloid clearance	Associated with neurodegenerative disease
Metabolic/Live- Associated	APOE	Apolipoprotein E	Lipid metabolism	Associated with cognitive aging, dementia risk
	IGFBP2	Insulin-like growth factor binding protein 2	Growth factor regulation	Aging-related decline in IGF signaling
	IGFBP4	Insulin-like growth factor binding protein 4	Growth factor regulation	IGF signaling control
Immune/Inflammatory System	GDF15	Growth/Differentiation Factor 15	Stress and inflammation response	Strongly rises with age; linked to frailty and multimorbidity
	CXCL17	C-X-C motif chemokine ligand 17	Immune cell chemotaxis	Marker of inflammaging
	LTF	Lactoferrin	Innate immune defense	Age-related inflammation
	SERPINA3	Alpha-1 antichymotrypsin	Inflammation regulation	Increases with chronic inflammation
	B2M	Beta-2 microglobulin	Immune system component	Rises with age, linked to frailty and mortality
Hormonal/Endocrine System	FSHB	Follicle-stimulating hormone subunit beta	Hormonal regulation	Reflects reproductive aging axis
	INHBA	Inhibin subunit beta A	Hormone and growth factor	Regulates cell proliferation
	AGRP	Agouti-related protein	Appetite regulation	Declines with age; energy balance
ECM/Connective Tissue	COL6A3	Collagen type VI alpha 3 chain	ECM	Reflects tissue stiffness and fibrosis
	ELN	Elastin	ECM	Declines with aging, reduced tissue elasticity
	MMP2	Matrix metalloproteinase 2	ECM remodeling	Tissue senescence and fibrosis
	TGFBI	Transforming growth factor beta-induced protein	ECM organization	Cellular senescence signaling

**Table 9:** Common evaluation metrics used in cancer diagnostics and biological aging prediction [391,392,393].

Abbreviation	Full Name	Meaning in Cancer Diagnosis
accuracy	Accuracy	Proportion of correctly classified cases (true positives + true negatives) among all samples.
AUC	Area Under the Receiver Operating Characteristic Curve	Overall diagnostic ability to discriminate cancer vs. non-cancer; higher AUC = better performance.
LoD	Limit of Detection	Lowest concentration of biomarker detectable above background, critical for early cancer detection.
SN/TPR	Sensitivity/True Positive Rate	Fraction of cancer cases correctly identified as positive (reduces false negatives).
SP/TNR	Specificity/True Negative Rate	Fraction of non-cancer cases correctly identified as negative (reduces false positives).
PPV	Positive Predictive Value	Probability that a positive result truly indicates cancer.
NPV	Negative Predictive Value	Probability that a negative result truly indicates absence of cancer.
Precision	Positive Predictive Accuracy	Fraction of predicted positives that are truly positive (closely related to PPV).
MAE	Mean Absolute Error	Regression error metric, sometimes applied in cancer risk or biological age prediction models.
RMSE	Root Mean Square Error	Another regression-based error metric, penalizes larger errors more heavily than MAE.

**Table 10:** Summary of liquid biopsy biomarkers for cancer detection.

Biomarker Category	Representative Molecules	Sample Type	Detection Platform/Technology	Key Performance	Evidence Level
ctDNA	EGFR, KRAS, BRAF, PIK3CA, TP53	Plasma, Serum	Digital PCR, NGS, BEAMing, ddPCR	LoD: 0.01–0.1%; SN 85–95%; SP 95–99%	FDA-approved (e.g., cobas EGFR v2)
CTCs	EpCAM, CK19, vimentin, HER2	Whole Blood	Microfluidic capture, immunomagnetic separation, RT-PCR	Recovery > 80%, SP >90%	CE-IVD/FDA Class I (ClearCell^®^ FX)
EVs	CD63, CD81, TSG101, Alix, HER2, PD-L1	Plasma, Urine, Saliva	Nanoplasmonic sensors, microfluidic EV chips, electrochemical detection	LoD: 10^2^–10^3^ vesicles/mL; SN 90%, SP 95%	Exploratory/Clinical validation ongoing
cfRNA, miRNA, lncRNA	miR-21, miR-155, miR-210, HOTAIR, MALAT1	Plasma, Serum, Urine	qRT-PCR, LAMP, NGS, NanoString	SN 80–95%; SP 85–98%; AUC 0.85–0.95	Clinical research/ retrospective cohorts
Protein Biomarkers	CEA, CA15-3, CA19-9, AFP, PSA, HER2	Plasma, Serum	ELISA, SERS, electrochemical biosensors, microfluidic immunoassay	SN 70–95%; SP 80–99%; LoD: pg–fg/mL	Multiple FDA-cleared assays
DNAm/Epigenetic Markers	SEPT9, SHOX2, RASSF1A, GSTP1	Plasma, cfDNA	Methylation-specific PCR, bisulfite sequencing	SN 75–90%; SP 85–95%; AUC 0.88–0.95	FDA-approved (Epi proColon)
Metabolites and Lipids	Choline, lactate, sphingomyelin	Plasma, Urine	LC–MS/MS, NMR spectroscopy, electrochemical sensors	SN 70–90%; SP 75–95%	Exploratory/ preclinical
Immune/Inflammatory Biomarkers	IL-6, CRP, TNF-α, YKL-40	Plasma, Serum	Multiplex ELISA, electrochemical immunosensor	SN 80–95%; SP 85–98%	Clinical validation (Large cohort)
Multi-omics/Integrated Panels	ctDNA + miRNA + EV proteins	Plasma, cfDNA, EVs	Hybrid microfluidic–AI systems, SERS–electrochemical fusion	AUC > 0.95; turnaround time < 1 h	Translational/emerging POC diagnostics

**Table 11:** Comparative Analytical Performance of Digital PCR and NGS-Based Platforms for Circulating Biomarker Detection.

Parameters	dPCR/ddPCR	NGS	Ref.
Detection Principle	Partition-based absolute quantification of mutant or methylated alleles	Sequencing of millions of cfDNA fragments with molecular barcodes	[483,484]
LoD	0.0005~0.01% VAF (1 mutant in 10^4^~10^6^ copies) for KRAS mutation	~0.1–6% Variant Allele Fraction for KRAS mutation	[485,486]
SN	KRAS detection: 81% Plasma HPV: 70% BRAFV600E mutation:100%	KRAS detection 65% Plasma HPV: 75%	[484,487,488]
SP	KRAS: 85% BRAF V600E:69.88%	KRAS: 88%, Advanced NGS:99.999%	[483,484,487]
Dynamic Range	3–5 orders of magnitude	3–8 orders of magnitude	[489,490,491,492]
Quantification Accuracy	Absolute quantification	Semiquantitative	[493]
Multiplexing Capacity	Low (≤10 targets/run)	High (>1000 targets/sample)	[488,489,493]
Turnaround Time	Short (~hours) (sample-to-result)	Long (~days) (library prep + sequencing)	[487,493]
Cost per Sample (approx.)	Low cost ($50~$300)	Less cost-effective for fewer than 20 targets ($300~$1500)	[486,494,495]
Instrumentation Complexity	Generally simpler equipment and straightforward data interpretation; POC feasible	extensive bioinformatics support and complex data interpretation; Laboratory-based	[493,495]
Clinical Applications	Minimal residual disease monitoring, targeted mutation tracking	Comprehensive mutation profiling, tumor heterogeneity analysis	[486,493,496,497]
Limitations	Limited multiplexing; requires prior mutation knowledge	Higher cost, longer TAT, bioinformatic complexity	[486,493]

Abbreviations: VAF; Variant Allele Fraction, LoD; Limit of Detection, POC; Point-of-Care, TAT; Turnaround Time.

**Table 12:** Comparison of HCC Detection Models [612].

Model	Type of Model	Input Features	Key Technical Approach	Overall HCC (All Stage) TPR *	Early-Stage HCC TPR	Notes
HES V2.0	Machine learning regression (Generalized Estimating Equation (GEE))	Age, ALT, platelets, etiology (viral/non-viral), AFP, AFP-L3, DCP, longitudinal changes in AFP/AFP-L3/DCP	GEE model + 10-fold cross-validation	47.2%	45.3%	Adds dynamic biomarker trends and clinical liver function indicators for improved SN
GALAD	Logistic regression	Gender, age, AFP, AFP-L3, DCP	Fixed regression equation	41.1%	39.8%	Widely validated; higher false-positive rate (~26% at common threshold)
ASAP	Logistic regression	Age, sex, AFP, DCP	Fixed regression equation	42.4%	40.7%	Performs better in viral cirrhosis; excludes AFP-L3
AFP alone	20 ng/mL in plasma			38.4%	39.8%	Baseline comparator

* True positive rate (TPR = SN).

**Table 13:** Comparative Summary of 510(k), De Novo, and PMA Regulatory Pathways [828].

Feature	510(k) Premarket Notification	De Novo Classification	PMA
Applicable Device Class	Class I–II (low to moderate risk)	Class I–II (novel, low-to-moderate risk without predicate)	Class III (high risk, life-supporting or sustaining)
Predicate Requirement	Required—substantial equivalence to a predicate device	No predicate—creates a new classification	Not applicable—new high-risk device
Regulatory Basis	Section 510(k) of the FD&C Act	1997 FD&C Act Amendment (Automatic Class III Designation)	Section 515 of the FD&C Act
Purpose	Demonstrate substantial equivalence to an existing marketed device	Reclassify novel but low-risk devices into Class I/II	Demonstrate safety and effectiveness through valid scientific evidence
Evidence Requirements	Bench and analytical testing; limited human data if necessary	More data than 510(k), possibly limited clinical data	Comprehensive preclinical and clinical studies (IDE trials)
Regulatory Review Intensity	Moderate; performance and equivalence-based	Moderate; risk-based review of novel technology	Highest; includes clinical and manufacturing inspection
Regulatory Outcome	FDA Clearance	FDA Grant (new classification and product code)	FDA Approval
Typical Review Time	~90 days	~120 days	≥180 days (often >1 year)
Post-market Controls	General and special controls	General and special controls	General/special controls + post-approval studies
Representative Devices	Glucose meter, blood pressure monitor, standard ELISA kits.	Guardant SHIELD™ (ctDNA-based early cancer detection).	FoundationOne CDx (tumor genomic profiling), Cobas EGFR Mutation Test.

Section 510(k) refers to a specific provision of U.S. law governing medical devices. Sec-tion 510(k) provides a less burdensome regulatory route compared to the full Pre-Market Approval (PMA) pathway, making it the primary path for many moder-ate-risk (Class II) devices. Section 510(k) is often relevant for diagnostics or biosensing technologies (such as those for aging or cancer biomarkers) that build on prior cleared devices—assuming a suitable predicate exists [829]. Section 515 is titled “Premarket Approval” (PMA) for medical devices. The PMA sub-mission must include full reports of all information (published or known) regarding investigations that show whether the device is safe and effective. If the device fails to meet the requirements under Section 515, it is considered adulterated and may not be legally marketed [830].

**Table 14:** Regulatory Status of Representative Cancer and Aging Biomarkers and Diagnostic Platforms.

Assay/ Platform	Analyte/ Biomarker(s)	Specimen Type	Regulatory Pathway/Region	Indication/ Context of Use	Evidence Level/ Validation Stage
Epi proColon^®^ (Epigenomics AG)	Methylated SEPT9 gene (cfDNA)	Plasma (blood)	FDA PMA (2016, U.S.); CE-IVD (EU)	CRC screening for average-risk adults ≥ 50 years	Prospective multi-center clinical trial (N ≈ 7900); post-market surveillance
Cologuard^®^ (mt-sDNA) (Exact Sciences)	Methylated NDRG4, BMP3, mutant K-ras, hemoglobin (Hb)	Stool	FDA PMA (2014, U.S.)—Class III device	Non-invasive CRC screening	Large pivotal trial > 10,000 participants; CMS-covered benefit
CellSearch^®^ (Menarini Silicon Biosystems)	CTC (EpCAM^+^/CK^+^/DAPI^+^/CD45^−^)	Whole blood	FDA 510(k) clearance (2004); CE-IVD	Prognosis and therapy monitoring in metastatic breast, prostate, CRC	Multicenter prospective validation; CLIA-certified deployment
FoundationOne CDx (Foundation Medicine Inc.)	300+ gene NGS panel—somatic mutations, CNAs, MSI, TMB	FFPE tissue	FDA PMA (2017, U.S.); EMA CE-IVD	Comprehensive genomic profiling/companion diagnostic for targeted therapies	Clinical bridging to multiple drug labels (≥30 oncology indications)
Guardant360 CDx (Guardant Health Inc.)	cfDNA mutations (>70 genes including EGFR, KRAS)	Plasma	FDA PMA (2020, U.S.)—liquid biopsy NGS panel	Genomic profiling for advanced solid tumors/therapy selection	Prospective and retrospective studies (N ≈ 5000); analytical cross-validation vs. tissue
Lumipulse G β-Amyloid 1-42/1-40 (Fujirebio)	Aβ1-42/Aβ1-40 ratio in CSF	CSF	FDA De Novo (2022, U.S.); CE-IVD	Aid in diagnosis of AD	Clinical performance ≥94% AUC vs. PET; multi-center validation
Lumipulse G p-tau217/Aβ1-42 (plasma) (Fujirebio)	Phosphorylated tau 217 and Aβ1-42	Plasma	FDA 510(k) clearance (May 2025, U.S.)	Blood-based aid to diagnose AD in symptomatic adults	Prospective cohort (N ≈ 1200); bridging to CSF/PET standards
Grail Galleri™ (MCED)	cfDNA methylation patterns (>100,000 CpG sites)	Plasma	CLIA LDT (2021, U.S.); FDA IDE trial ongoing	Multi-cancer early detection (screening)	Large case–control (>15,000)/PATHFINDER prospective study (NCT04241796)
Olink Explore 3072 Proteomics	3072 proteins (panel)	Plasma/ Serum	Research-use-only (RUO); under clinical validation	Aging and multi-disease risk stratification (‘Inflammaging’)	Longitudinal population cohorts (SCANDAT, UK Biobank); pre-regulatory stage

Abbreviations: cfDNA; cell-free DNA, NGS; next-generation sequencing, CNA; copy-number alteration, MSI; microsatellite instability, TMB; tumor mutational burden, Aβ; amyloid-β, AD; Alzheimer’s disease, RUO; research use only, CSF; Cerebrospinal fluid, CLIA; Clinical Laboratory Improvement Amendments, IDE; Investigational Device Exemption.

**Table 16:** Improved Technologies for Early Diagnosis.

Previous Approach	Improved Technology	Key Advancements	Impact	Ref.
Conventional imaging (mammography, CT, MRI, PET) interpreted manually by radiologists	AI-augmented imaging using CNN and LSTM	Automatically extracts hierarchical image features; reduced subjectivity and fatigue-related errors	Higher diagnostic accuracy and efficiency; enables early detection of subtle lesions missed by humans	[553,554,555,569,585,592,683]
Single-biomarker tests (e.g., protein-based tumor markers)	Liquid biopsy using ctDNA, CTCs, EVs	Detects multiple tumor-derived analytes in blood ono-invasively	Enables earlier, minimally invasive cancer detection and real-time treatment monitoring	[409,464,465]
Tissue biopsy for molecular profiling	Multi-omics integration (genomics, proteomics, metabolomics) with AI	Combines large-scale molecular datasets; distinguishes normal aging vs. pathology	Improves precision of diagnosis and risk stratification in both cancer and aging	[546,599,601,602]
Manual feature engineering in ML models	Automated feature extraction using DL	Learns complex, high-dimensional patterns from data (images, genomics)	Eliminates bias from manual selection; boosts accuracy and scalability	[612,677,714]
Chronological age-based risk assessment	BAG models from MRI	Estimates biological brain age to detect accelerated aging and cognitive decline risk	Allows earlier intervention before clinical symptoms appear	[258]

## Data Availability

The original contributions presented in this study are included in the article. Further inquiries can be directed to the corresponding author.

## Abbreviations

The following abbreviations are used in this manuscript:

Abbreviation	Full Term	Category/Context
AD	Alzheimer’s Disease	Neurodegenerative disorder/aging research
AFP	Alpha-Fetoprotein	Clinical protein biomarker (HCC, germ cell tumors)
AGEs	Advanced Glycation End Products	Metabolic/oxidative stress biomarkers
AI	Artificial Intelligence	Computational analysis/biosensing
ATM	Ataxia Telangiectasia Mutated	DNA damage response/telomere dysfunction
ATR	ATM and Rad3-Related	DNA damage response/telomere dysfunction
AUC	Area Under the Curve	Diagnostic performance metric
AuNPs	Gold Nanoparticles	Probe for biosensing
Aβ	Amyloid Beta Protein	Neurodegeneration/Alzheimer’s biomarker
BAG	Brain Age Gap	Neuroimaging biomarker for biological aging
BAP	Brain Age Prediction	AI-based neuroaging estimation
BC	Breast Cancer	Solid tumor type
BRCA	Breast Cancer Gene (BRCA1/2)	Tumor suppressor genes
CA125	Cancer Antigen 125	Clinical protein biomarker (Ovarian cancer)
CA19-9	Carbohydrate Antigen 19-9	Clinical protein biomarker (Pancreatic cancer)
CAD	Computer-Aided Detection	AI-assisted image analysis
CD4	Cluster of Differentiation 4	Immune cell surface marker (T-helper cells)
CD8	Cluster of Differentiation 8	Cytotoxic T-cell surface glycoprotein
CDK	Cyclin-Dependent Kinase	Cell cycle regulation
CCL	Chemokine (C–C Motif) Ligand	Inflammatory/immune signaling
CEA	Carcinoembryonic Antigen	Clinical tumor biomarker (CRC, BC)
cfDNA	Cell-Free DNA	Liquid biopsy/genetic biomarker
cfRNA	Cell-Free RNA	Liquid biopsy/transcriptional biomarker
CLIA	Clinical Laboratory Improvement Amendments	a U.S. law that sets quality standards
ChIP-seq	Chromatin immunoprecipitation sequencing	Analysis of Protein-DNA interactions
CK	Cytokeratin	Structural epithelial protein
CKD	Chronic Kidney Disease	Metabolic/renal disorder
CNN	Convolutional Neural Network	Deep learning model architecture
CRC	Colorectal Cancer	Solid tumor type
CRP	C-Reactive Protein	Inflammatory/cardiovascular biomarker
CT	Computed Tomography	Diagnostic imaging modality
CTCs	Circulating Tumor Cells	Liquid biopsy/cancer detection
ctDNA	Circulating Tumor DNA	Liquid biopsy/genetic biomarker
cTnI	Cardiac Troponin I	Cardiac injury biomarker
CVD	Cardiovascular Disease	Chronic disease/heart pathology
CXCL	Chemokine (C–X–C Motif) Ligand	Immune/inflammatory signaling
CXCR	Chemokine (C–X–C Motif) Receptor	Immune/inflammatory signaling
ddPCR	Droplet Digital Polymerase Chain Reaction	Genomic detection platform
DL	Deep Learning	Subfield of AI for data modeling
DNAm	DNA Methylation	Epigenetic biomarker
DNMT3A	DNA (Cytosine-5)-Methyltransferase 3A	Epigenetic modification enzyme
ECM	Extracellular Matrix	Tumor microenvironment structure
EGFR	Epidermal Growth Factor Receptor	Oncogenic driver/therapeutic target
ELISA	Enzyme-Linked Immunosorbent Assay	Protein quantification assay
EMT	Epithelial-to-Mesenchymal Transition	Cancer invasion/metastasis mechanism
EpCAM	Epithelial Cell Adhesion Molecule	Diagnostic and prognostic marker
ER	Estrogen Receptor	Hormone signaling/BC subtype
ERCC1	Excision Repair Cross-Complementation Group 1	DNA repair enzyme
EVs	Extracellular Vesicles	Intercellular communication/biomarker source
FDA	U.S. Food and Drug Administration	Regulatory agency
FET	Field-Effect Transistor	Electronic biosensing platform
GFAP	Glial Fibrillary Acidic Protein	Neurodegeneration biomarker
GDF15	Growth Differentiation Factor 15	Aging/metabolic biomarker
HbA1c	Hemoglobin A1c	Glycemic control/diabetes biomarker
HCC	Hepatocellular Carcinoma	Solid tumor type
HER2	Human Epidermal Growth Factor Receptor 2	Breast/gastric cancer biomarker
HK2	Hexokinase 2	Glycolytic enzyme/metabolic marker
HPV	Human Papillomavirus	Viral oncogenic infection
HSCs	Hematopoietic Stem Cells	Aging and regeneration biology
IDO	Indoleamine 2,3-Dioxygenase	Immune-metabolic enzyme
IFN	Interferon	Immune signaling cytokine
IGF	Insulin-Like Growth Factor	Growth/metabolic regulation
IL	Interleukin	Inflammatory cytokine family
IVD	In Vitro Diagnostic	Regulatory classification
KRAS	Kirsten Rat Sarcoma Viral Oncogene Homolog	Oncogenic mutation marker
LAMP	Loop-Mediated Isothermal Amplification	Rapid nucleic acid detection
LC	Lung Cancer	Solid tumor type
LC–MS/MS	Liquid Chromatography–Tandem Mass Spectrometry	Proteomic/metabolomic detection
LDHA	Lactate Dehydrogenase A	Metabolic enzyme/hypoxia biomarker
LDTs	Laboratory-developed tests	Diagnostic tests
lncRNAs	long non-coding RNA	Non coding RNA
LoD	Limit of Detection	Analytical sensitivity parameter
MAE	Mean Absolute Error	Statistical model evaluation metric
MAPK	Mitogen-Activated Protein Kinase	Signal transduction pathway
MCED	Multi-Cancer Early Detection	Blood-based screening assay
MD	Microfluidics Device	Technology used in POCT
miRNA	MicroRNA	Post-transcriptional regulatory molecule
ML	Machine Learning	AI method for pattern recognition
MMP	Matrix Metalloproteinase	Proteolytic/tumor invasion enzyme
MRI	Magnetic Resonance Imaging	Diagnostic imaging technique
MRS	Magnetic Resonance Spectroscopy	Detecting tool
mTOR	Mechanistic Target of Rapamycin	Nutrient-sensing/autophagy regulation
mtDNA	Mitochondrial DNA	Oxidative stress/aging biomarker
NF-κB	Nuclear Factor Kappa-light-chain-enhancer of Activated B Cells	Inflammatory transcription factor
NGS	Next-Generation Sequencing	Genomic profiling platform
NSCLC	Non-Small Cell Lung Cancer	Solid tumor type
NT-proBNP	N-terminal Pro-B-type Natriuretic Peptide	Heart failure biomarker
OC	Ovarian Cancer	Gynecologic malignancy
PC	Pancreatic Cancer	Solid tumor type
PCa	Prostate Cancer	Solid tumor type/biomarker target
PDAC	Pancreatic Ductal Adenocarcinoma	Pancreatic cancer subtype
PD-L1	Programmed Death Ligand-1	Immune checkpoint biomarker
PET	Positron Emission Tomography	Functional imaging technique
PI3K	Phosphoinositide 3-Kinase	Cell survival/growth signaling
PMA	Premarket Approval	FDA regulatory pathway
POC	Point-of-Care	Decentralized diagnostic setting
POCT	Point-of-Care Testing	Decentralized diagnostic setting
PSA	Prostate-Specific Antigen	FDA-approved diagnostic biomarker
PTEN	Phosphatase and Tensin Homolog	Tumor suppressor gene
qRT-PCR	Quantitative Real-Time Polymerase Chain Reaction	Gene expression quantification
RAGE	Receptor for Advanced Glycation End Products	Oxidative stress/aging biomarker
RB	Retinoblastoma Protein	Tumor suppressor/cell cycle control
SASP	Senescence-Associated Secretory Phenotype	Aging/inflammatory response
SCL	Smart Contact Lens	Glucose sensing
SCLC	Small Cell Lung Cancer	Pulmonary malignancy subtype
SCs	Stem Cells	Regenerative medicine context
SERS	Surface-Enhanced Raman Spectroscopy	Plasmonic biosensing method
SN	Sensitivity	Diagnostic performance metric
SP	Specificity	Diagnostic performance metric
SPR	Surface Plasmon Resonance	Optical biosensing platform
STAT	Signal Transducer and Activator of Transcription	Cytokine/growth factor signaling
T2D	Type 2 Diabetes	Metabolic disease
TCGA	The Cancer Genome Atlas	Genomic data resource
TERT	Telomerase Reverse Transcriptase	Telomere maintenance enzyme
TF	Transcription Factor	Gene expression regulator
TG	Tear Glucose	Glucose sensing
TME	Tumor Microenvironment	Cancer biology/pathology
TNF-α	Tumor Necrosis Factor Alpha	Inflammatory cytokine
VEGF	Vascular Endothelial Growth Factor	Angiogenesis/vascular signaling
WGS	Whole Genome Sequencing	Genomic profiling platform
